# Neutron Diffraction and Spectroscopic Studies of Intramolecular Tetrel Bonds in Three Fluorinated Zinc Complexes: Significant Redshift in the sp^3^ C–H Stretch Confirmed by Experiments and Theory

**DOI:** 10.1021/jacs.5c15040

**Published:** 2025-11-26

**Authors:** Norman Lu, Gurumallappa Gurumallappa, Pin-Yu Liu, Ka-Long Chan, Yu-Cheng Huang, Yu-Ching Lin, Yun-Ting Hsieh, Pin-Xiang Zeng, Yashwanth Gowda, Meng-Hsun Tsai, Eskedar Tessema, Huan-Cheng Chang, Joseph S. Francisco

**Affiliations:** 1 Institute of Organic and Polymeric Materials, 34877 National Taipei University of Technology, Taipei 106, Taiwan (ROC); 2 Graduate Institute of Energy and Optoelectronic Materials, 34877 National Taipei University of Technology, Taipei 106, Taiwan ROC; 3 Institute of Atomic and Molecular Sciences, 38017 Academia Sinica, Taipei 106, Taiwan (ROC); 4 Department of Earth and Environmental Science and Department of Chemistry, 6572 University of Pennsylvania, Philadelphia, Pennsylvania 19104-6316, United States

## Abstract

Three fluorinated Zn complexes were synthesized and characterized. Their single crystals were grown and studied via X-ray and neutron diffraction methods. These fluorinated metal complexes tend to form two special noncovalent interactions: an intramolecular tetrel bond (TB) and an intramolecular blueshifting hydrogen bond (HB). In 4FH-ZnCl_2_ [2,6-(HCF_2_CF_2_CH_2_OCH_2_)_2_-py-ZnCl_2_], an intramolecular TB induces significant elongation of a C–H bond in a methylene group, H1A–C1–H1B, to 1.108(2) Å, whereas the intramolecular C1–H1A···F2 blueshifting HB shortens the C1–H1A bond to 1.092(1) Å. Thus, C1–H1B is longer than C1–H1A by 16(2) mÅ such that either one of the C1–H1 stretches becomes a methine-like stretch with a peak appearing at 3020 or 2688 cm^–1^. A peak corresponding to a lengthened sp^3^ C1–H1B bond is greatly redshifted and appears at 2688 cm^–1^. When this 2688 cm^–1^ peak is compared with its other methylene C–H peak (3020 cm^–1^), their C–H vibrational stretches then differ by 332 cm^–1^, which is likely the largest ever sp^3^ C–H bond stretching difference reported between two methylene C–H bonds. Additionally, two C–D bonds of deuterated 4FH-ZnCl_2_, which has an intramolecular C···F TB that induces very different lengths of the two C–D bonds in a D–C–D group, become a local mode, as proven by Hooke’s law. Thus, Badge’s rule has been extended by using both neutron and spectroscopic studies.

## Graphical abstract

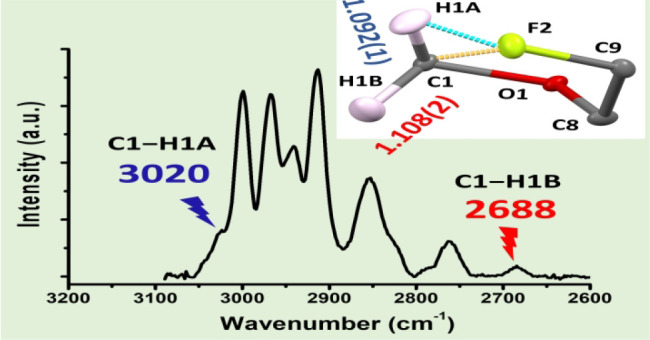

## Introduction

Noncovalent interactions (NCIs) are known to play a very important role in many fields of science, and their influence can change the physical properties of a substance^1^ and the biological functions of organisms.^2^ These interactions can be used to design molecules,^3^ bind small molecules with big macromolecules^4^ and understand many reaction pathways.^5^ Two kinds of NCIs are mainly discussed in this study: a tetrel bond (TB)^6^ and a hydrogen bond (HB).^7^ A TB is a type of NCI that arises from the electrostatic interaction between a Lewis base and a Lewis acid (Group 14 element). The term ″tetrel″ is derived from the Latin word ″tetra,″ which means four, referring to group 14 elements. The most commonly studied TBs involve C, Si, and Ge as Lewis acids. TBs are known to play a crucial role in catalysis,^8^ crystal engineering,^9^ supramolecular chemistry,^10^ biochemistry,^11^ SN2 mechanism,^5^ the C–H bond strength,^13,14^ etc. The updated TB review article by Scheiner et al. has also been published recently.^12^ A HB, which is mainly the blue-shifting HB being discussed in this study, is one of the most studied interactions in this field,^15^ but researchers have also shown interest in other weak interactions such as σ-hole, π-hole, anion-π and cation-π interactions.^16^ It is the blue-shifting HBs that is the focus of this study. This nonclassical HBs which are hydrophobic are very different in nature from the classical HBs. The impact of a TB on the C–H bond length,^17^ which is the opposite of the impact caused by improper hydrogen bonding,^18^ elongates the C–H bond according to Bent’s rule.^19^ To understand how the elongation induced by a TB affects the sp^3^ C–H bond, the number of C–H bonds present at the same carbon must be determined. If the factor is >1, then the elongation of each C–H bond needs to be equally factored by being divided by the number of C–H bonds present. For example, in a methylene group, the elongation of each C–H bond in the H–C–H moiety is determined as a factor of 2, as this moiety has two C–H bonds, as recently reported in a 2023 paper.^17^ For HBs, the concept of C-based H-bonds focuses on improper nonclassical HBs, that is, C–H···**A**, where **A** is usually an electronegative atom, which is also known as an improper HB^20–23^ (Scheme S1 in the SI). The fluorinated Zn complexes in this study tend to easily form large and stable single crystals due to the presence of some F-related NCIs, e.g., intramolecular TBs, blueshifting C–H···F HBs, and blueshifting C–H···X–M (metal halide). Here, the study of the neutron structures of three new **fluorinated** Zn complexes, 2,6-(**R**_**f**_CH_2_OCH_2_)_2_-py-ZnX_2_, where R_f_= a short-fluorinated chain and X = Cl or I [abbreviated as **4FH-ZnCl**_**2**_(**I**), **4FCl-ZnI**_**2**_(**II**) and **2FCl-ZnI**_**2**_(**III**)], is the main theme herein. It is found that the normal Badger-type bond length–bond strength (BLBS) relation is observed for both HB and TB NCIs.^24^ The intrinsic strength of each C–H bond investigated herein has been determined by the local stretching force constant which can usually be obtained by using PyMOL calculations.^25–27^ In addition, TB-induced changes in the lengths of the two C–H bonds in the H–C–H group are revealed in detail. A recent neutron paper is believed to prove Badger’s rule, which says that^28^ ″the strength of a bond (in terms of the force constant) correlates with the bond length”.^29^ In particular, in biological chemistry and protein-related areas, Badger’s rule which in 2010 was further extended by KLC to the polyatomic system based on the adiabatic internal coordinate modes (AlCoMs) method,^30^ has been largely used to calculate the metal and O distances by using the reduced mass in Hooke’s law for metalloenzyme systems containing metals (e.g., Fe, and Cu) and oxo bonds.^31^

## Experimental Section

### General Procedures

Gas chromatographic/mass spectrometric data were obtained using an Agilent 6890 Series gas chromatograph with a series 5973 mass-selective detector. The reactions were monitored with an HP 6890 GC using a 30-m HP-1 capillary column with a 0.25 mm stationary phase film thickness. The flow rate was 1 mL/min, with splitless injection. Infrared spectra were obtained on a PerkinElmer RX I FT-IR spectrophotometer. NMR spectra were recorded on Bruker AM 500 and 300 spectrometers using 5–mm o.d. sample tubes. D_2_O, CDCl_3_, and DMSO–*d*_*6*_ were used as references for both the ^1^H and ^13^C NMR spectra, whereas refrigerant–11 (CFCl_3_) was used as a reference for the ^19^F NMR spectra. The chemicals, reagents, and solvents employed were commercially available, purchased from either Aldrich (Taipei, Taiwan) or SynQuest (FL, USA) and used as received.

### Synthesis of the 4FH-ZnCl_2_(I) Complex [2,6-(HCF_2_CF_2_CH_2_OCH_2_)_2_-py-ZnCl_2_]

#### [2,6-(HCF_2_CF_2_CH_2_OCH_2_)_2_-py-ZnCl_2_]

A 25 mL single-necked round-bottomed flask was equipped with a magnetic stirrer and then sequentially filled with 2,6-(HCF_2_CF_2_CH_2_OCH_2_)_2_-py (7.2 mmol, 1605 mg), ZnCl_2_ (7.2 mmol, 1605 mg) and dichloromethane (DCM, ca. 5 mL). Under a N_2_ atmosphere, this mixture was then stirred overnight with the round-bottom flask immersed in an oil bath at room temperature. At the end of the reaction, the solvent was removed by a vacuum system such that a white powder remained as a crude product.^17^ The solid product was collected and dried in an oven at 60 °C to obtain the **4FH-ZnCl**_**2**_(**I**) complex in 90% yield (779 mg). Samples of the **4FH-ZnCl**_**2**_(**I**) complex (50 mg) were separately dissolved in DCM/hexane (1:7 v/v) for crystallization.

Note: Synthesis^15b,17^ of the **4FCl-ZnI**_**2**_(**II**) complex [2,6-(ClCF_2_CF_2_CH_2_OCH_2_)_2_-py-ZnI_2_] and 2FCl-ZnI_2_(**III**) complex [2,6-(ClCF_2_CH_2_OCH_2_)_2_-py-ZnI_2_] & their deuterated species. The synthetic procedure was almost identical to that for the **4FH-ZnCl**_**2**_(**I**) complex.

#### Analytical Data for 4FH-ZnCl_2_(I)

^**1**^ **H NMR** (400 MHz, DMSO-*d*_6_, ppm): δ= 7.89 (1H, t,^3^ *J*_HH_= 7.8 Hz, H4), 7.40 (2H, d,^3^ *J*_HH_= 7.8 Hz, H3/5), 6.59 (2H, tt,^3^ *J*_HF_= 52 Hz,^4^ *J*_HF_= 4 Hz, C_2_F_4_H), 4.71 (4H, s, py-CH_2_), 4.07 (4H, t,^3^ *J*_HF_= 13.9 Hz, OCH_2_). ^**13**^ **C NMR** (125 MHz, CDCl_3_, ppm) δ= 153.7, 141.3, 121.8 (5C, s, py), 117.2 ∼ 106.6 (4C, OCH_2_CF_2_CF_2_H), 71.6 (2C, s, py-CH_2_), 68.2, 68.0, 67.8 (2C, t, OCH_2_CF_2_CF_2_H). ^**19**^ **F NMR** (500 MHz, CDCl_3_, ppm) δ = −122.32 (4F, s, OCH_2_CF_2_CF_2_H), −137.96 (4F, s, OCH_2_CF_2_CF_2_H). **FT-IR** (ATR, cm^–1^) υ= 3104, 3086, 3053 (sp^2^ C–H), 3008 (C–H; – CF_2_H), 2948, 2923, 2858 (sp^2^ C–H), 1614 (C = C; – py), 1584 (C = N; – py), 1105, 1090, 1063, 1029, 1014 (C–F). **HR-FAB** (M-Cl, *m*/*z*=): *m*/*z* calcd for C_13_H_13_^35^ClF_8_NO_2_Zn 465.9799, found 465.9790; *m*/*z* calcd for C_13_H_13_^37^ClF_8_NO_2_Zn 467.9767, found 467.9765. Yield = 90%; m.p = 108–110 °C.

#### Analytical Data for 4FCl-ZnI_2_(II)

^**1**^ **H NMR** (400 MHz, DMSO-*d*_6_, ppm) δ= 7.88 (1H, t,^3^ *J*_HH_= 7.8 Hz, H4), 7.35 (2H, d, ^3^ *J*_HH_= 7.8 Hz, H3/5), 4.73 (4H, s, py-CH_2_), 4.30 (4H, t, ^3^ *J*_HF_ = 12.3 Hz, OCH_2_CF_2_CF_2_Cl). ^**13**^ **C NMR** (150 MHz, CDCl_3_, ppm) δ=153.9 ∼ 122.4 (5C, s, py), 114.6, 112.9 (4C, t, OCH_2_CF_2_CF_2_Cl), 71.0 (2C, s, py-CH_2_), 67.4, 67.3, 67.1 (2C, t, OCH_2_CF_2_CF_2_Cl). ^**19**^ **F NMR** (564.7 MHz, CDCl_3_) −70.3 ppm (4F, s, OCH_2_CF_2_CF_2_Cl), −116.3 ppm (4F, s, OCH_2_CF_2_CF_2_Cl); **FT-IR** (ATR, cm^–1^) ν = 3077, 3051 (sp^2^ C–H), 2977, 2936, 2859 (sp^3^ C–H),1613 (C = C; – py), 1581 (C = N; – py), 1143, 1088 (C–F). **HR-FAB** (M-I, *m*/*z*=): *m*/*z* calcd for C_13_H_11_^35^ClF_5_INO_2_Zn 533.8735, found *m*/*z*: 533.8647; *m*/*z* calcd for C_13_H_11_^37^ClF_5_INO_2_Zn 535.8674, found *m*/*z*: 535.8634. Yield = 92%. m.p = 124 °C.

#### Analytical Data for 2FCl-ZnI_2_(III)

^**1**^ **H NMR** (400 MHz, CDCl_3_, ppm) δ = 8.04 (1H, t,^3^ *J*_HH_= 7.8 Hz, H4), 7.44 (2H, d, ^3^ *J*_HH_= 7.8 Hz, H3/5), 5.15 (4H, s, py-CH_2_), 4.45 (4H, t, ^3^ *J*_HF_= 27.8 Hz, OCH_2_CF_2_Cl). ^**13**^ **C NMR** (150.9 MHz, CDCl_3_, ppm) δ = 153.9, 141.4, 122.4 (5C, s, py), 128.2, 126.3, 124.3,122.4 (2C, t, OCH_2_CF_2_Cl), 73.1 (2C, s, py-CH_2_), 67.4, 67.3, 67.1 (2C, t, OCH_2_CF_2_Cl). ^**19**^ **F NMR** (564.7 MHz, CDCl_3_, ppm): δ= −59.2 ppm (4F, s, OCH_2_CF_2_Cl); **FT-IR** (ATR, cm^–1^) ν = 3102, 3083, 3056 (sp^2^ C–H), 2986, 2937, 2876, 28612852 (sp^3^ C–H), 1617 (C = C; – py), 1586 (C = N; – py), 1110, 1090, 1070, 1033 (C–F); **HR-FAB** (M-I, *m*/*z*=): *m*/*z* calcd for C_11_ H_11_^35^Cl_2_F_4_N_1_O_2_Zn_1_I_1_ 525.8439, found *m*/*z*:525.8502; *m*/*z* calcd for C_11_H_11_^35^Cl ^37^ClF_4_N_1_O_2_Zn_1_I_1_ 527.8410, found *m*/*z*: 527.8505; *m*/*z* calcd for C_11_H_11_^37^Cl_2_F_4_N_1_O_2_Zn_1_I_1_ 529.8417, found *m*/*z*: 529.8396. Yield = 91%. m.p = 189.8 °C.

## Results and Discussion

### A Experimental Studies

#### A1 Structural Ways

The fluorinated complexes give rise to some improper HBs in their single-crystal neutron structures. Recently, fluorinated Zn complexes were shown to be able to bring two fluorinated side chains close to a metal core, ZnX_2_, to facilitate the formation of intermolecular and intramolecular TBs. The intermolecular C···F TB, the former, has been recently reported, and the latter (intramolecular bond) appears in all three fluorinated Zn complexes reported herein, causing significant elongation of combined methylene C–H bonds. For two of the three Zn complexes, the combined elongations of their C–H bonds are approximately 16 mÅ in **I** and **II**, and two methylene C–H bonds from the same C differ in the bond length by >13 mÅ according to neutron studies. Two recent neutron findings^17^ are as follows: 1. The magnitude of the intermolecular TB-induced combined C–H bond elongation is ∼ 10 mÅ. 2. The unusual C–H bond change is related to two F-based NCIs, i.e., i. a C···F TB (inducing C–H bond elongation) and ii. a C–H···F blueshifting HB (causing C–H bond shortening). The rare intramolecular TB, as a good model, present in these 3 new fluorinated ZnX_2_ complexes [**I–III** (X = Cl, I)] can be schematically studied, so the magnitude of its influence on the C–H bond length can be well determined and unprecedentedly studied through the high-resolution neutron diffraction method. This type of intramolecular TB involves F, which induces a weak interaction with the central C, and its two methylene C–H bond lengths are significantly changed. For example, one C1–H1 bond is significantly elongated due to intramolecular TB formation. The other C1–H1 bond is then significantly shortened, mainly due to the combination of intramolecular and intermolecular C–H bonds related to improper HBs. There are two side chains of the Zn complex with two fused 5-membered rings of the O–N–O moiety. As shown in Figure 1, the right C(1)­H_2_O­(1)­C­(8)­H_2_C­(9)­F_2_–CF_2_H and the left C(7)­H_2_O­(2)­C­(11)­H_2_C­(12)­F_2_–CF_2_H chains have cis and trans conformations with respect to the C1–O1 and C7–O2 single bonds, respectively. Thus, on its right side, **H1A–C1–O1–C8–C9–F2** forms a six-membered chair form with a very short H1A···F2 distance of 2.323(2) Å. Interestingly, in this 6-membered chair form (see Scheme S1), the very rare intramolecular TB is detected for the first time. Owing to this special intramolecular TB interaction with a very short C1···F2 distance of 2.922(1) Å measured by neutron diffraction, the intramolecular TB length is 2.922(1) Å, which is ∼ 0.22 Å shorter than that reported in a recent 2023 paper.^17^ Owing to this unusual intramolecular C1···F2 TB, the combined elongation of the two methylene C–H bonds induced by the TB is estimated to be ∼ 16(2) mÅ. The other C1–H1A length, which is also supposed to lengthen by the same extent to 1.108(2) Å, is further offset to 1.092(1) Å due to several combined improper HBs (e.g., C1–H1A···F2, C1–H1A^···^F8 and C3–H3···H1A–C1). Thus, the free C1–H1B bond in **4FH-ZnCl**_**2**_(**I**) is elongated by 8(2) mÅ, so the TB-induced combined elongation of two methylene C1–H1 bonds is estimated to be 16 mÅ (=2 × 8) when the offset from the aforementioned improper HBs is not considered (or present). Thus, the C1–H1B and C1–H1A bonds from a H–C1–H moiety in **4FH-ZnCl**_**2**_(**I**) are 1.108(2) and 1.092(1) Å in Figure 1, respectively. Thus, two methylene C–H bonds become a local mode via their C–H stretches owing to the significant difference in the two C–H bond lengths. Based on Badger’s rule,^28^ the vibrational local mode is experimentally observed for the first time, which is due to a significant difference in the methylene C–H bond lengths (i.e., force constants), as described by Hooke’s law (in the SI). In other words, the C–H bond local mode is made possible by the large difference in the numerator (i.e., force constant) instead of in the denominator (i.e., the reduced mass) in Hooke’s law. Additionally, as shown in Figure 2, the neutron structure of **4FCl-ZnI**_**2**_(**II**) also shows a 6-membered ring with a H1A–C1–O1–C8–C9–F2 orientation and a similar intramolecular TB bond. The length of this intramolecular C1···F2 TB is 2.956(1) Å. This TB bond induces the C1–H1B bond to elongate to 1.108(2) Å, and C1–H1A is also elongated to the same extent; then, this elongation is mainly followed by offsetting by the intramolecular C1–H1A···F2 blueshifting HB such that the other methylene C1–H1A bond length becomes 1.095(4) Å. Again, this effective intramolecular C1···F2 TB results in the combined elongation of two methylene C–H bonds by 16 mÅ. The left C7–O2–C11–C12–C13–Cl2 chain adopts a trans conformation, so no intramolecular 6-membered ring is formed on the left side. Thus, the C7–H7B bond length is 1.101(3) Å, and the C7–H7A bond length is 1.099(3) Å, i.e., the length of either free C–H bond in this H–C7–H moiety is ∼ 1.100 Å while no TB is present. The caption in Figure 2 shows the geometric information regarding this intramolecular TB. For example, the intramolecular blueshifting HB of C1–H1A···F2 with an H1A and F2 distance of 2.351(3) Å is shown in Figure 2. Thus, this type of fluorinated pincered Zn complexes tends to form an intramolecular 6-membered ring in the H1–C1–O1–C–C–F2 chain.

**Figure 1:**
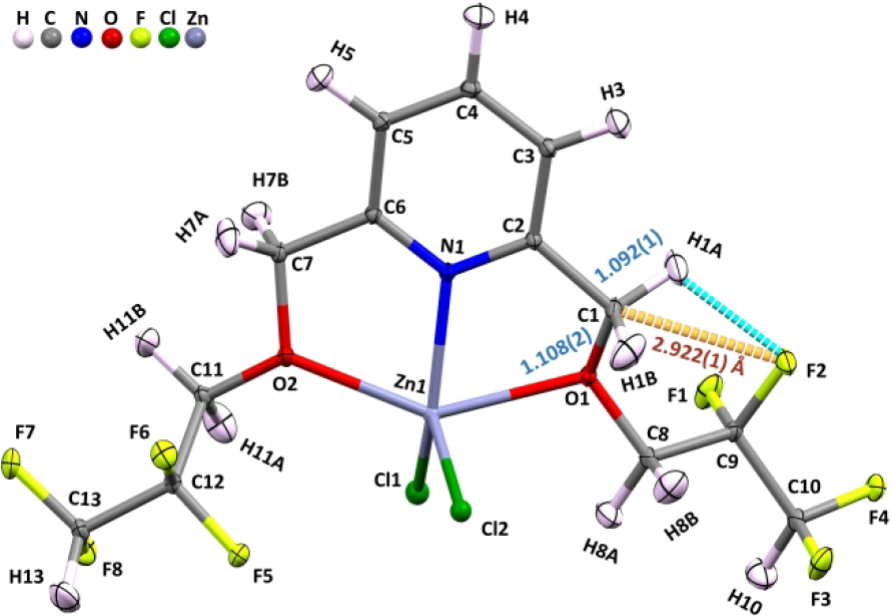
ORTEP diagram of the **4FH-ZnCl**_**2**_(**I**) neutron structure with an intramolecular TB depicted. [d­(C···F)=2.922(1) Å; C1–H1B = 1.108(2) and C1–H1A = 1.092(1) Å]. For other selective C–H bond data, see Table S5.

**Figure 2:**
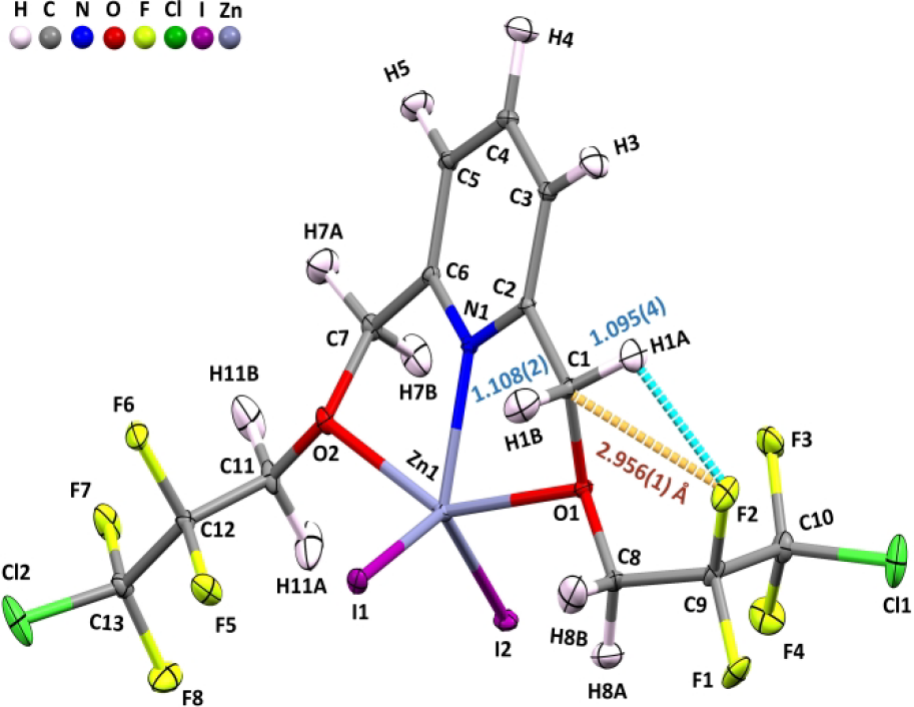
ORTEP diagram of the **4FCl-ZnI**_**2**_ **(II)** neutron structure with an intramolecular TB depicted. [d­(C···F)=2.956(1) Å; C1–H1B = 1.108(2) and C1–H1A = 1.095(4) Å]. For other selective C–H bond data, see Table S8.

As shown in Figure 3, this complex **III** not only has a 6-membered chair form but also forms an intramolecular C1···F1 TB. **2FCl-ZnI**_**2**_(**III**) also has this type of intramolecular TB in its right chain. Figure 3 also reveals a 6-membered form similar to those reported in Figures 1 and 2. The length of the C1···F1 TB in Figure 3 is ∼ 3.120 Å, which is close to the 3.142 Å value recently reported.^17^ In a 2023 study,^17^ an intermolecular TB with a length of ∼ 3.120 Å was found to induce the combined elongation of two C–H bond(s) by ∼ 10 mÅ. Similarly, the C1···F1 TB present in complex **III** also results in the combined elongation of two methylene C–H bonds by ∼ 10 mÅ. The elongation of the C1–H1A bond of **2FCl-ZnI**_**2**_ measured as 1.103(2) Å, as shown in Figure 3, is mainly due to the moderate C1^···^F1 TB-induced C1–H1A bond elongation. The C1–H1B bond is first elongated to the same amount [1.103(2) Å] due to the TB but is then shortened, mainly due to the significant intramolecular C1–H1B···F1 blueshifting HB and other intermolecular NCIs, to 1.092(1) Å. The special structural features among the complexes (**I–III**) are the intramolecular TB and blueshifting HBs, which make the two C–H bonds in the H–C–H group in the 6-membered ring (on the right side) significantly differ in the C–H bond lengths. These special features, which have never been previously reported, have a great impact on the local mode vibration and Badger’s rule. Thus, in this section, these special intramolecular TB-induced methylene groups among complexes **I–III** are vigorously investigated. Taking **4FH-ZnCl**_**2**_(**I**) as an example, the difference between the two methylene C–H bond lengths of 16 mÅ [= 1.108(2)-1.092(1)] is believed to be the largest difference in two (sp^3^) C–H bond lengths for the same C atom. This C–H bond length difference in the germinal C (in the H–C–H group) clearly satisfies the 3-sigma rule. This TB-induced combined elongation of two methylene C–H bonds by 16 mÅ is likely to be unprecedented, to our knowledge.

**Figure 3:**
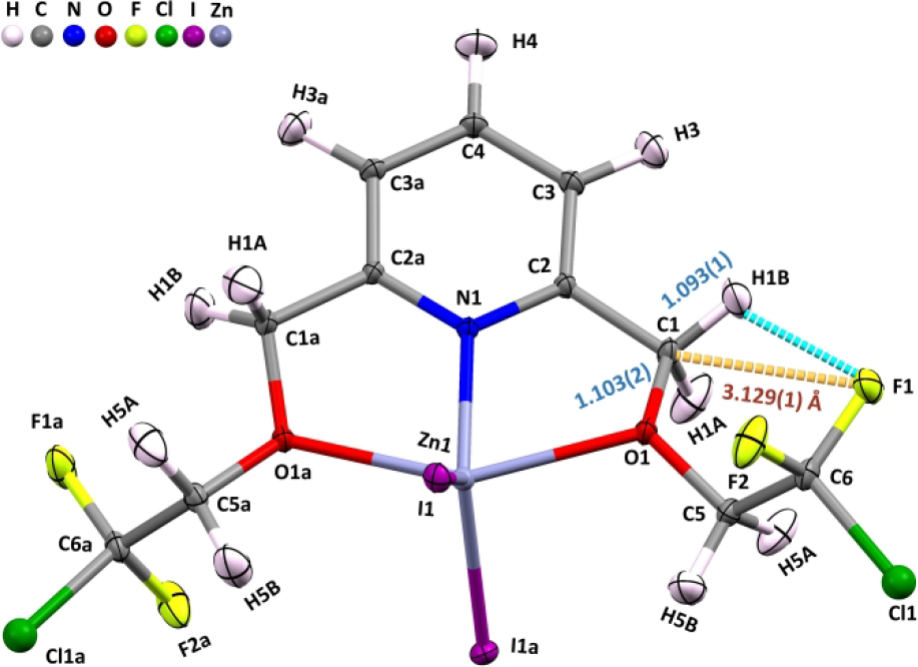
ORTEP diagram of the **2FCl-ZnI**_**2**_(**III**) neutron structure with the intramolecular TB indicated [d­(C···F)=3.129(1) Å; C1–H1B = 1.093(1) and C1–H1A = 1.103(2) Å]. For other C–H bond data, see Table S13.

In this study, this H–C–H group with a local mode is shown by experimentally structural studies (which described above) and spectroscopical investigations which are discussed below.

#### A2 Spectroscopies

[In addition to the neutron data shown in Figure.s 1–3,] The C–H vibrational data were also experimentally studied via vibrational spectroscopies (both FT-IR and Raman). As shown in Figure 4, the greatly red-shifted peak of the C1–H1B stretch, due to a significant C1^···^F2 TB interaction, then appears at 2688 cm^–1^, with increasing intensity in its red-shifted IR peak. Additionally, the vibrational (FT-IR and Raman) spectra of the (elongated) referenced (pivotal) lengths of C1–H1B from **4FCl-ZnI**_**2**_ and C1–H1A from **2FCl-ZnI**_**2**_ were also carefully assigned based on FT-IR data and confirmed by Raman spectroscopy data, as shown in Figure 6 & Figure S4, respectively. The C1–H1B stretch from **II** appears at 2702 cm^–1^, and the C1–H1A stretch from **III** occurs at 2817 cm^–1^ (see Figure 4b,c). These two vibrational values were also used to theoretically calculate the other C–H stretching peak(s) shown in the theory section of Table 1. Thus, the methylene C1–H1 bonds in complexes **I–III** with two C–H bonds differing in length by ≥ 10 mA can be clearly confirmed to satisfy the local mode of the methylene C–H bonds.

**Figure 4:**
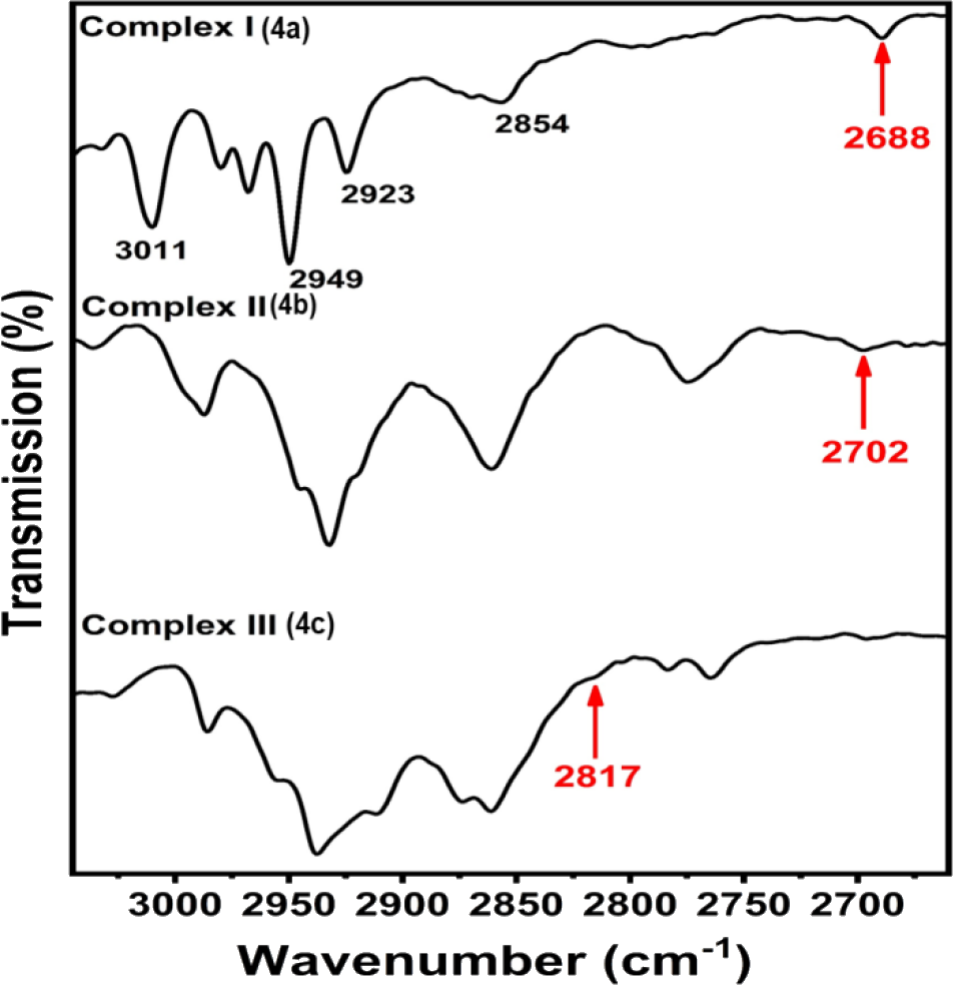
Vibration spectrum of complexes (**I–III**); a) **4FH-ZnCl**_**2**_(**I**). The FT-IR spectrum shows that the C1–H1B vibrational peak is greatly red-shifted by ∼ 280 cm^–1^ to 2688 cm^–1^; b) **4FCl-ZnI**_**2**_(**II**) vibration and c) **2FCl-ZnI**_**2**_(**III**) vibration. Notes: 1. **II** has a strongly red-shifted sp^3^ C1–H1B bond (= 1.108(2) Å) with IR peak occurring at 2702 cm^–1^. 2. **III** has a red-shifted sp^3^ C1–H1A bond (1.103(2) Å) with IR peak at 2817 cm^–1^.

**Table 1:** Local Mode Studies of **I–III** Based on Experimental Neutron Structural Data, Force Constant, and Gaussian Calculations^a^

		**length (calc.)**	**stretch (cm** ^**–1**^ **)**	**using the neutron length** ^b^	**stretch (cm** ^**–1**^ **)**	**(calculated) local mode force constants** **(mdyn** */* **Å)**
**(I)**	C–H bond in H–C–H					
	elongated C–H bond	1.1068		**1.1080**		5.083
	red-shifted stretch		3041		**2688**	
	shortened C–H bond	1.0989		1.1001		5.363
	blueshifted stretch		3156		2861^c^	
**(II)**	**C–H bond in H–C–H**					
	elongated C–H bond	1.1059		**1.1080**		5.117
	red-shifted stretch		3050		**2702**	
	shortened C–H bond	1.0986		1.1002		5.374
	blueshifted stretch		3161		2872	
**(III)**	**C–H bond in H–C–H**					
	elongated C–H bond	1.1066		**1.1030**		5.089
	red-shifted stretch		3040		**2817**	
	shortened C–H bond	1.0991		1.0955		5.359
	blueshifted stretch		3154		2998	

a DFT is a very functional theory that cannot predict the C–H bond elongation well for complexes **I–III**, so only the MP2 results are reported (see SI).

b Using the (elongated) neutron bond length as **a pivotal point**.

c^32^ The factor (=0.975) of anharmonic to harmonic stretch is used to calculate this blue-shift peak.

Three complexes in Figure 4 are investigated by vibrations (FT-IR and Raman). In complex **I**, its H1B–C1 bond elongation, with no other NCI interactions, is solely induced by a significant C1^···^F2 TB. However, the H1A–C1 bond length is reported to be shorter than 1.1001 Å (see Table 1) by ∼ 8 mÅ, i.e., 1.092(1) Å. As shown in Figure 1 and its neutron data, this result is obtained because in the solid state, the H1A–C1 bond has other intermolecular blueshifting HBs that make it even shorter in addition to the significant intramolecular improper C1–H1A^···^F2 HB causing C1–H1A bond shortening. In the solid state, both the C1–H1A and C1–H1B bonds in **4FH-ZnCl**_**2**_ have even larger differences in force constants such that the stretching C1–H1A and C1–H1B bonds certainly vibrate in a local mode. Thus, independent C1–H1A and C1–H1B stretches appear at 3020 and 2688 cm^–1^ in Figure 5a, respectively. Both **II** and **III** also have short intramolecular TBs being 2.956(1) and 3.129(1) Å, respectively. Thus, their C1–H1-related vibrations also show the local modes. As shown in Figure 6, two independent C1–H1A and C1–H1B stretches appear at 2702 and 2949 cm^–1^, respectively. Similarly, complex **III** also has two independent methylene C–H stretches appearing at 2817 and 2962 cm^–1^ which are from C1–H1B and C1–H1A vibrations (see SI), respectively.

**Figure 5:**
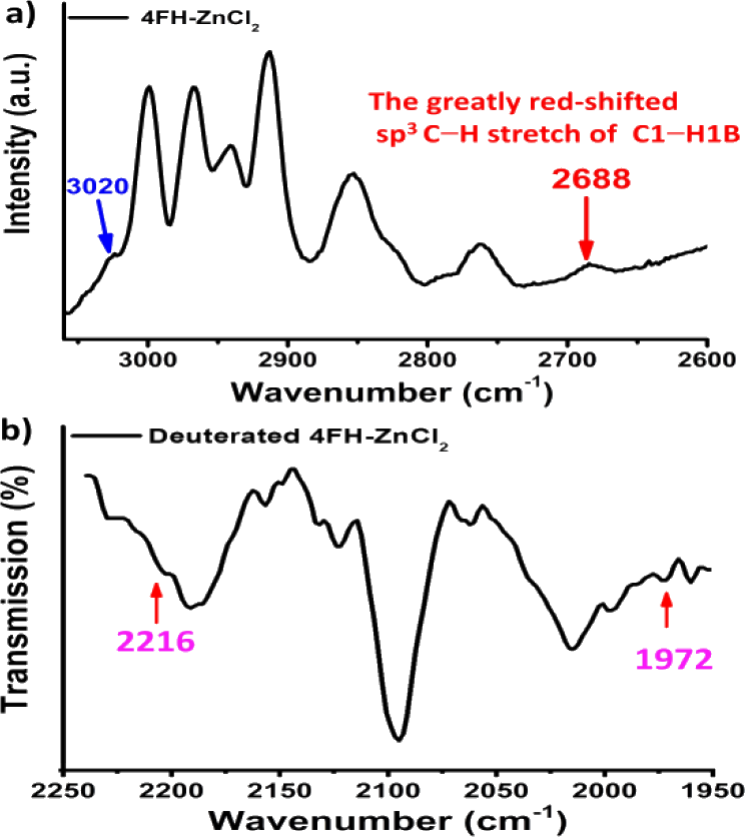
Vibrational spectra of **4FH-ZnCl**_**2**_ **(I)** and its deuterated analogue. a) Raman spectrum (the IR spectrum is shown in Figure 4a), and b) FT-IR spectrum of the deuterated species.

**Figure 6:**
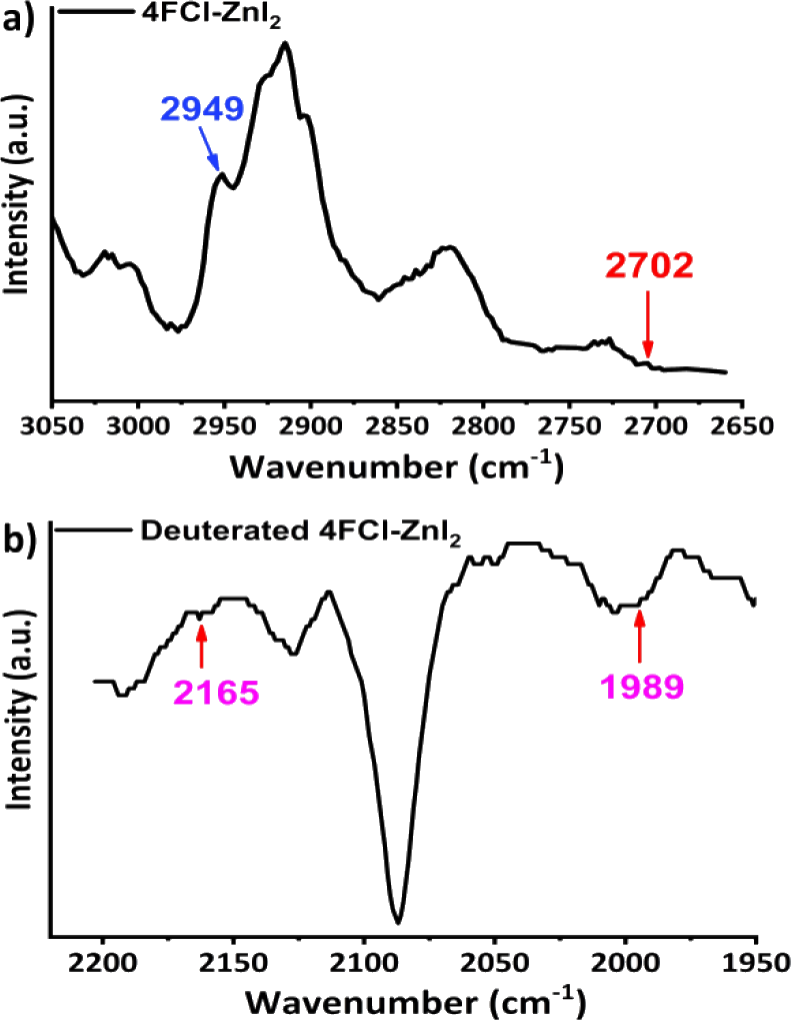
Vibrational spectra of **4FCl-ZnI**_**2**_ **(II)** and its deuterated analogue. a) Raman spectrum (the IR spectrum is shown in Figure 4b), and b) FT-IR spectrum of the deuterated **II** species.

In addition, all three deuterated complexes **I–III** have also successfully prepared. Take the deuterated **4FH-ZnCl**_**2**_(**I**) complex for example, its isotope studies of two C–H bonds in the C1–H_2_ group could be readily carried out for deuterated complex **I**, which can further confirm the correct assignments, to verify the local modes of two C–H bonds of this specific methylene group. The vibrational spectra of **4FH-ZnCl**_**2**_(**I**) and its deuterated analogue in the solid state are induced by a significant C1^···^F2 TB. However, the H1A–C1 bond length is reported to be shorter than 1.1001 Å (see Table 1) by ∼ 8 mÅ, i.e., 1.092(1) Å. As shown in Figure 1 and its neutron data, this result is obtained because in the solid state, the H1A–C1 bond has other intermolecular blueshifting HBs that make it even shorter in addition to the significant intramolecular improper C1–H1A^···^F2 HB causing C1–H1A bond shortening in the solid state.

The Raman spectrum of the **4FH-ZnCl**_**2**_(**I**) complex in the solid state is shown in Figure 5a. As shown in Figure 1, the neutron ORTEP diagram of **4FH-ZnCl**_**2**_(**I**) in the solid state shows that the bond lengths of the H1B–C1 and H1A–C1 bonds are 1.108(2) and 1.092(1) Å, respectively. The elongated H1B–C1 bond data [=1.108(2) Å] are the same as those reported in Figure 5 (& Table 1). In the solid state, both the C1–H1A and C1–H1B in **4FH-ZnCl**_**2**_ have even larger differences in force constants such that the stretching C1–H1A and C1–H1B bonds certainly vibrate in a local mode. Thus, independent C1–H1A and C1–H1B stretches appear at 3020 and 2688 cm^–1^ in Figure 5, respectively. Additionally, two IR independent vibrational peaks of deuterated **4FH-ZnCl**_**2**_ in the solid state appear at 2216 and 1972 cm^–1^ in Figure 5b, respectively. If the respective bond force constants of the H1A–C1 and C1–H1B bonds in **I** are k1 and k2, then k1 and k2 should have the same values before and after deuteration of the specific C1-based methylene group in **4FH-ZnCl**_**2**_(**I**). In fact, after deuteration of **4FH-ZnCl**_**2**_, the factors of 3020/2216 and 2688/1972 are the same [= 1.36, which is the square root of {(12 + 1)­x2/(12 + 2)­x1}] when Hooke’s law is used for calculation (see Scheme S3). In other words, a local mode of the methylene group in **I** is well proven both experimentally and theoretically. Furthermore, Badge’s rule can then be further confirmed using the neutron and spectroscopic studies for this CH_2_ system (i.e., triatomic system).

Additionally, vibrational comparisons of complexes **II** and its deuterated analogue are shown in Figure 6. Like those shown in Figure 5, the related (local mode) two methylene C–H bonds have been replaced with two C–D bonds in the deuterated **4FCl-ZnI**_**2**_ **(II)**. And two new respective methylene C–D peaks appeared at 2165 and 1989 cm^–1^ in the FT-IR spectrum (in Figure 6b). In the **III** case, the comparisons of their vibrational spectra shown in Figure S4 in SI are also consistent with those described for complexes **I** & **II**.

Accordingly, as shown in Figure 2, the neutron ORTEP diagram of **4FCl-ZnI**_**2**_(**II**) in the solid state shows that the bond lengths of the H1B–C1 and H1A–C1 bonds are 1.108(2) and 1.095(4) Å, respectively. The elongated H1B–C1 bond data [=1.108(2) Å] are the same as those reported in both Figure 2 and Table 1, and this H1B–C1 bond elongation, with no other NCI interactions, is solely induced by a significant C1^···^F2 TB. However, the H1A–C1 bond length is reported to be shorter than 1.1002 Å (in Table 1) by ∼ 7 mÅ. As shown in Tables 1, 2, this result is obtained because the H1A–C1 bond in the solid state has other intermolecular blueshifting HBs which make it even shorter in addition to the significant intramolecular improper C1–H1A^···^F2 HB causing C–H bond shortening. In the solid state, both the C1–H1A and C1–H1B bonds in **4FCl-ZnI**_**2**_(**II**) have even larger differences in force constants such that the stretching C1–H1A and C1–H1B bonds also vibrate in a local mode. Therefore, independent C1–H1A and C1–H1B vibrations occur at 2949 and 2702 cm^–1^ in Figure 6, respectively. Additionally, the vibrational FT-IR spectrum of deuterated **4FCl-ZnI**_**2**_(**II**) in the solid state is shown in Figure 6b. The independent C1–D1A and C1–D1B stretches then occur at 2165 and 1989 cm^–1^, respectively. In a similar case to complex **I**, the respective bond force constants of the H1A–C1 and C1–H1B bonds in **II** are k1 and k2, then both k1 and k2 should be the same before and after deuteration for the specific C1-based methylene group in **4FCl-ZnI**_**2**_(**II**). Again, in the deuterated **4FCl-ZnI**_**2**_(**II**), the factors of 2949/2165 and 2702/1989 are identical [= 1.36, which is the square root of the reduced mass of H/D and C atoms when Hooke’s law is used for calculation. In other words, like the case of complex **I**, a local mode of the methylene group in **II** is well proven herein by experimentally spectroscopic studies. Thus, in the case of complex **III,** the local mode of H–C–H group can also be easily calculated (see Figure S4) accordingly. Thus, as shown in Figure S4, k1 (=2962/2177) and k2 (=2817/2070) have the same value of 1.36. In other words, a local mode of the CH_2_ group for complexes **I–III** has been unambiguously confirmed.

**Table 2:** Calculated C–H Bond Lengths of Monomeric **I–III** and Elongated C–H Bond Lengths from the Experimental Neutron Data of **I–III** ^a^ ^,^ ^b^ ^,^ ^c^

#	C–H bond length	**MP2 (monomer)**	**neutron data (Å)**	**comments**	
1	**I**	C1–H1A	1.1011	<1.1011^d^	with C1–H1A···F8 & C1–1A···F2 intermolecular HBs
2		C1–H1B	(1.1080)	1.108(2)	
3	**II**	C1–H1A	1.1001	<1.100^d^	with C1–H1···I2 & C1–H1A···F3 intermolecular HBs
4		C1–H1B	(1.1080)	1.108(2)	
5	**III**	C1–H1A	(1.1030)	1.103(2)	
6		C1–H1B	1.0955	<1.0955^d^	with C1–H1B···I1 intermolecular HB

a C1–H1A in **I** and **II** and C1–H1B in **III** are shortened (see SI).

b Two methylene C–H bond lengths are affected mainly by the intramolecular TB and improper HBs, so the shortened C–H bond discussed here is mainly considered to involve significant intramolecular interactions.

c The MP2 data reported here are factored data. Before factoring, the calculated values are (1.1068, 1.0989), (1.1059, 1.0986) and (1.1066, 1.0991) Å for **I–III**, listed in the calc. length column in Table 1.

d When one or more improper HB is added,^17^ the C1–H bond becomes even shorter.

Based on both experimental neutron structure data and vibrational spectroscopic studies of complexes **I–III**. Thus, the relationship of vibrational C–H frequency vs the C–H bond length can be plotted under the local mode. Either the C1–H1A or C1–H1B stretching vibration in this study can be simply predicted by Hooke’s law. The wavenumber vs bond length plot (whose table is provided in Table S14 the SI) is shown in Figure 7. Its derived equation is Y= −5.1991 × 10^–5^ X + 1.2483 (eq 1), with R^2^= 98.6%. This is the first experimental plot of the C–H bond length vs frequency mainly based on the C–H bond neutron length under local mode. Because now all complexes **I–III** have also been deuterated, the plot of the wavenumber vs C–D bond length (which is supposed to be the C–H bond length and its table is provided in Table S15 the SI) of all deuterated complexes is also shown in Figure 8 [the equation is Y= −7.1357 × 10^–5^ X + 1.2496 (eq 2); R^2^= 98.6%.]

**Figure 7:**
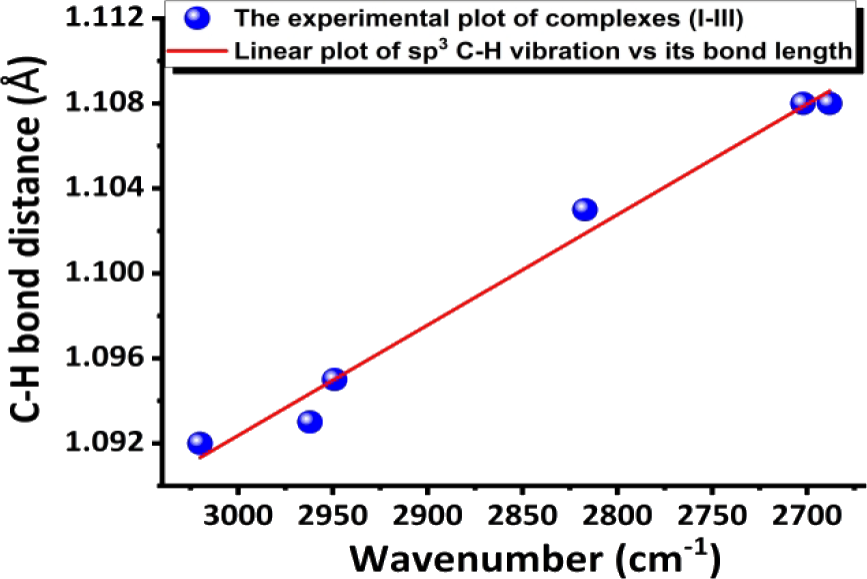
Experimental plot of the wavenumber vs neutron C–H bond length; the equation is Y= −5.1991 × 10^–5^ X + 1.2483 (with R^2^= 98.6%) (eq 1).

**Figure 8:**
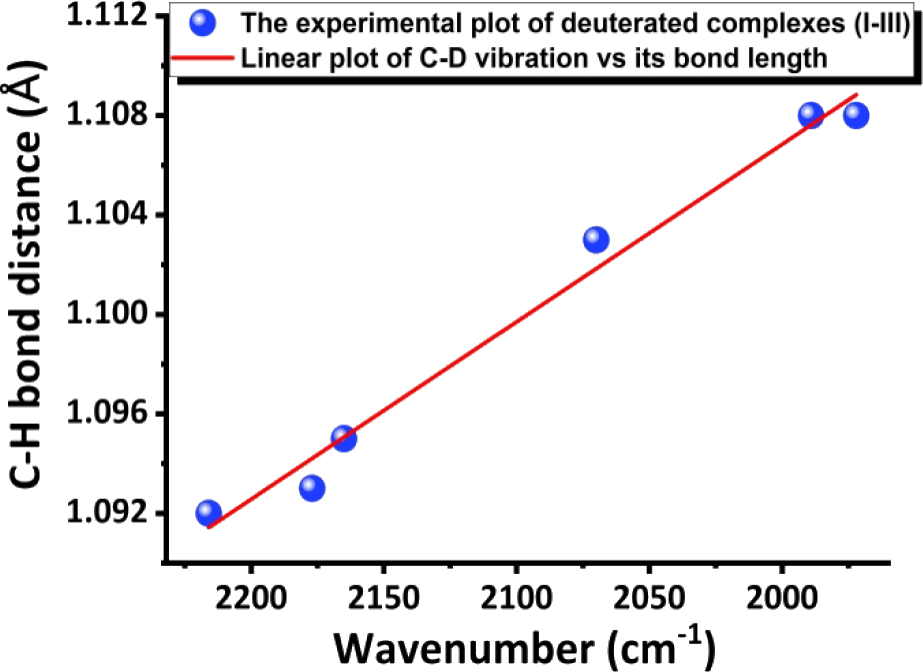
Experimental plot of the wavenumber vs C–D bond length; the equation is Y= −7.1357 × 10^–5^ X + 1.2496; R^2^= 98.6%.

This plot of three deuterated species is certainly to further confirm the local mode data of methylene C–H bonds from complexes **I–III**. In addition to their experimental studies by neutron structures and vibrational spectroscopies, the theoretical studies are also carried out below.

### B Theory

#### B1 Local Mode Plots (by Structurally Deuterated & MP2 Calculations for Vibrations)

In this study, this H–C–H group with a local mode is experimentally shown above and theoretically verified below (also see mainly the MP2 method in computational section B1 in SI). As shown in Figure 9, three Raman spectra of theoretically deuterated **4FH-ZnCl**_**2**_(**I**) in the gas phase, which contains the intramolecular C1^···^F2 TB with a specific C1-based methylene group, were well studied, with D–C1–H, H–C1–D and H–C1–H as three cases, as shown in Figure 9 (right side). The calculated vibrational (e.g., Raman) spectra of these three possible C1-based methylene groups are shown in the top, middle and bottom panels of Figure 9. Owing to one deuterium (D) substitution in the C(1)­H_2_ group, the other C1–H bond in this C1-based methylene group then vibrates independently without coupling to its geminal C1–D bond. In other words, the blue C–H stretching peak (top) and the red C–H peak (middle), which act like methine C1–H bond(s), are simple C–H stretching peaks arising from the so-called “local mode”. The remaining H atoms in complex **I** were also replaced with deuterium (D) atoms. Figure 9 shows the calculated Raman spectra of these three possibilities, with the one blue peak, one red peak and two black peaks in the top, middle and bottom spectra, respectively, corresponding to the C–H stretching peaks of **4FH-ZnCl**_**2**_(**I**). For both the D–C1–H and H–C1–D cases in **4FH-ZnCl**_**2**_(**I**), which has an intramolecular TB, owing to one deuterium (D) substitution, the C1–H bond in this C1-based methylene group then vibrates independently. Therefore, based on a local mode, the elongated C1–H1B stretching peak (as a pivotal point) appears at **2688** cm^–1^, and the peak of the other C1–H1A bond, which has only one intramolecular C1–H1A^···^F interaction according to the gas phase calculation that shortens this C1–H1A bond, appears at 2861 cm^–1^ in the H–C–H case (in the black trace at the bottom of Figure 9) of complex **I**.

**Figure 9:**
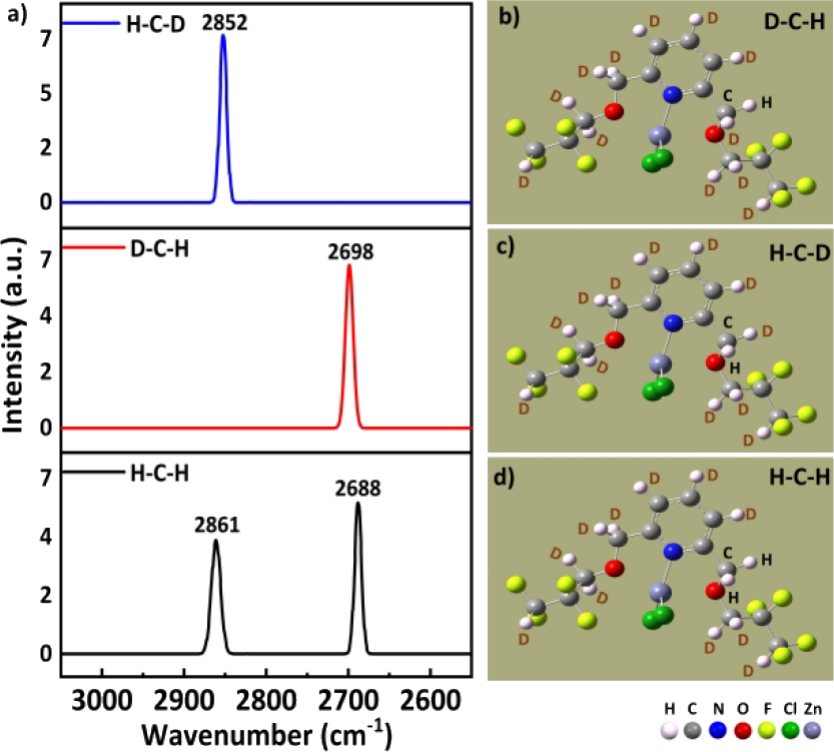
a) Raman spectra of the deuterated complexes, with the blue, red and black curves in the top, middle and bottom panels showing the spectra of **4FH-ZnCl**_**2**_ **(I)**, respectively; three molecular images of **4FH-ZnCl**_**2**_ **(I)** for the b) D–C–H, c) H–C–D and d) H–C–H cases.

Furthermore, its left C–H stretching peak (=2852 cm^–1^; top blue trace) and right C–H stretching peak (=2698 cm^–1^; middle red trace) correspond well in wavenumbers to the left (=2861 cm^–1^) and right (=2688 cm^–1^) C–H stretching peaks (bottom trace) in Figure 9, respectively. Thus, these results imply that the two C–H bonds in the H–C1–H case of **4FH-ZnCl**_**2**_ **(I)** (in the gas phase) can vibrate independently via a local mode because this C1 atom mainly has a short intramolecular C1^···^F2 TB of 2.922 Å and a shortened C1–H1A length due to the intramolecular C1–H1A^···^F2 blueshifting HB making its two methylene C–H bonds significantly differ in bond length.

Complexes **II** and **III**, both with short C1^···^F intramolecular TB interactions, also have the intramolecular TB and blueshifting HB influences. Thus, with one D atom substitution in the H–C1–H unit, the other C1–H bond in this C1-based methylene group also vibrates independently without coupling to its other C1–D bond. Thus, via a local mode, the elongated C1–H1B stretching peak, which acts as a pivotal point, appears at **2702** cm^–1^, and the other C1–H1A peak occurs at 2872 cm^–1^ in the H–C1–H case (in the black trace at the bottom of Figure 10) of complex **II**. Additionally, its left C–H stretching peak (=2859 cm^–1^; top blue trace) and right C–H stretching peak (=2714 cm^–1^; middle red trace) correspond well in wavenumbers to the left (=2872 cm^–1^) and right (= 2702 cm^–1^) C–H vibrational peaks (bottom trace) in Figure 10, respectively.

**Figure 10:**
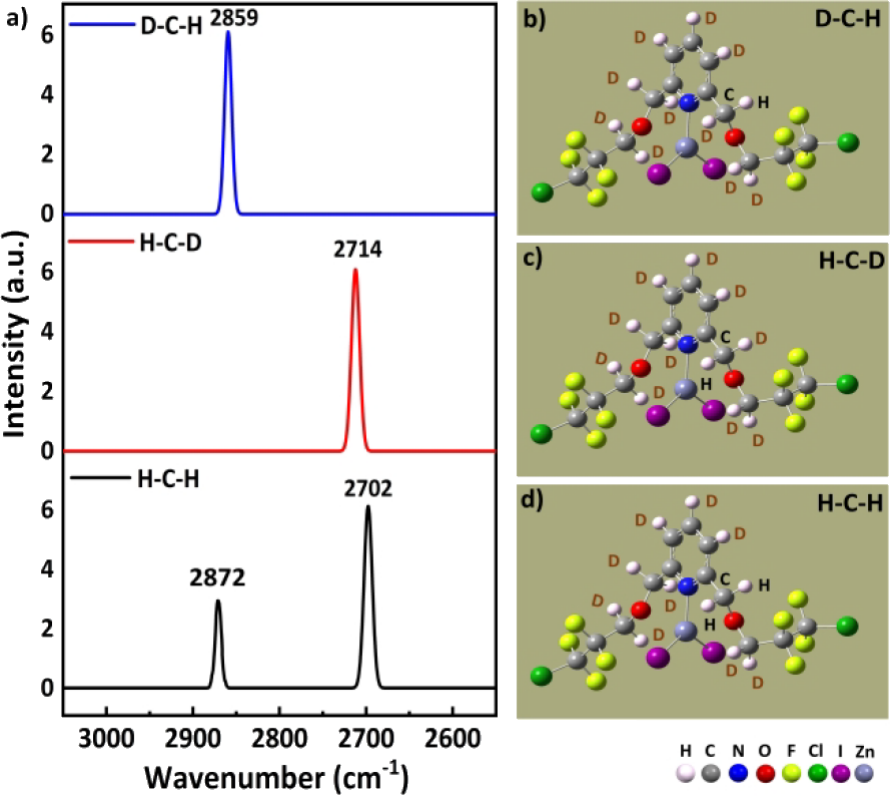
a) Raman spectra of the deuterated complexes, with the blue, red and black curves in the top, middle and bottom panels showing the spectra of **4FCl-ZnI**_**2**_, respectively; molecular images of **4FCl-ZnI**_**2**_ for the b) D–C–H, c) H–C–D and d) H–C–H cases.

For complex **III**, the simulation of three Raman spectra of deuterated **2FCl-ZnI**_**2**_ with a specific C1-based methylene group, for three cases of D–C1–H, H–C1–D and H–C1–H, was also successfully carried out, as shown in Figure 11. Similarly, its left C–H stretching peak (=2990 cm^–1^; top blue trace) and right C–H stretching peak (=2835 cm^–1^; middle red trace) correspond well in wavenumbers to the respective left (=2998 cm^–1^) and right (= 2817 cm^–1^) C–H vibrational peaks in the bottom trace of Figure 11. The results of these extended studies based on both experimental neutron data and MP2 calculations performed on H–C1–H cases of complexes **I–III** in the gas phase, which help to demonstrate that complexes **I–III** with intramolecular TBs within H–C1–H groups can show local modes for these specific methylene C–H bonds, are summarized in Table 1 above.

**Figure 11:**
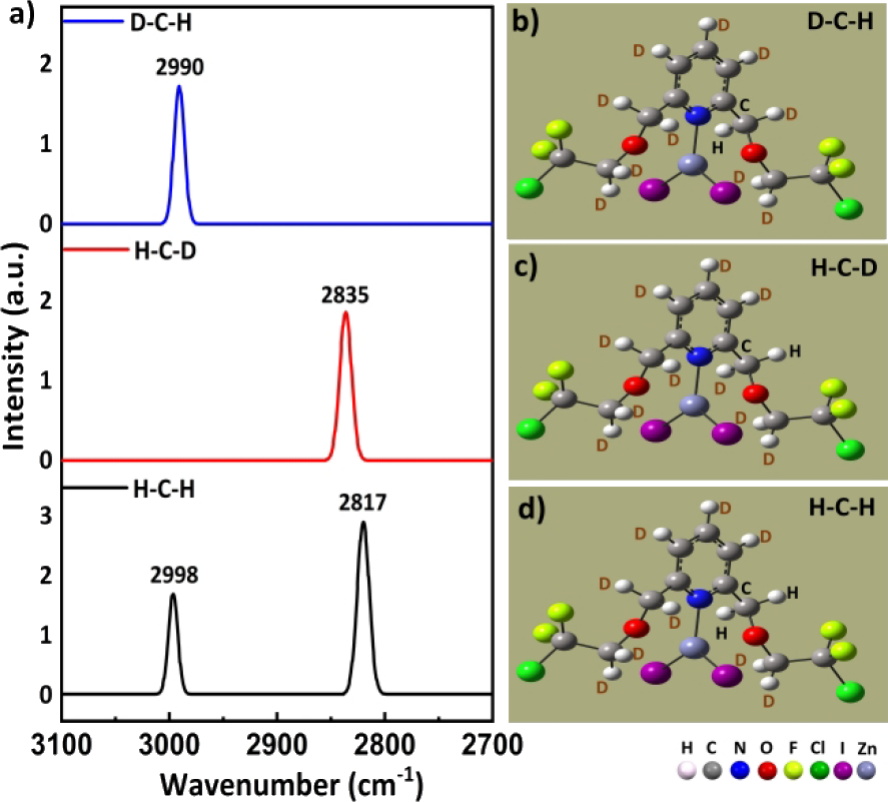
a) Raman spectra of the deuterated complexes, with the blue, red and black curves in the top, middle and bottom panels showing the spectra of **2FCl-ZnI**_**2**_(**III**), respectively; molecular images of **2FCl-ZnI**_**2**_ for the b) D–C–H, c) H–C–D and d) H–C–H cases.

In other words, the results of the plots of the CH_2_ group vibrations corresponding to a local mode shown in Figure.s 9–11 combined with the neutron data are summarized in Table 1. Taking the complex **I** (in the gas phase) for example, the calculated C1-based CH_2_ group has C1–H1A and C1–H1B bond lengths of 1.0989 and 1.1068 Å, respectively, and the calculated C1–H1A and C1–H1B C–H vibration wavenumbers are 3156 and 3041 cm^–1^, respectively. The elongated C1–H1B bond, whose length was obtained via high-resolution neutron analysis and was used as a reference length in this theoretical study, is usually a free C–H bond (with no other weak interactions). Thus, the two C–H bond lengths in **I** are determined to become **1.108** (elon- gated) and 1.1001 (shortened) Å, as shown in Table 1. In addition, their calculated vibration wavenumbers after calibration are 2688 and 2861 cm^–1^, respectively. Similarly, the calculated vibration results of the two other complexes (**II** and **III**) were similarly obtained. (Note: For example, the elongated C–H bond lengths of the two complexes (**II** and **III**) are 1.1080 Å in **II** and 1.1030 Å in **III**, as shown in Table 1.) Their shortened C–H bonds (presented in Table 1), considering only one intramolecular C1–H^···^F improper HB in monomeric **II** and **III**, are then determined to become 1.1002 and 1.0955 Å, respectively. Thus, the calculated vibrational results of the two shortened C–H bonds for the respective **II** and **III** monomers are then calibrated to be 2872 and 2998 cm^–1^. Then, there is a linear relationship between the C–H bond length and frequency of a H–C–H group for complexes **I–III** (in gas phase) such that the linear plot in Figure 12 can be obtained. In other words, in this study, we can experimentally and theoretically reach the local mode of a C–H bond vibration beyond McKean’s way of using the D substitution method to change the reduced mass calculation in Hooke’s law. Thus, the linear plot of these neutron C–H bond lengths and frequencies (in cm^–1^) can then be well derived by using neutron C–H bond data and vibrational C–H stretching data from **I–III** in Table 1. Interestingly, for the first time, a theoretical equation Y= −4.2096 × 10^–5^X + 1.2213 (eq 3) is obtained, with R^2^= 99.2% (shown in Figure 12). Another linear equation of Y= −3.5129 × 10^–5^X + 1.2008 (eq 4), which excludes the three referenced C–H lengths (as pivotal points), is also plotted (with R^2^= 99.5%) and shown in Figure S5 in the SI. (Note: The experimentally derived equation, eq (1), which is based on neutron data is shown in Figure 7, is in a good agreement with this theoretically derived eq (3). Therefore, these results clearly show that two C–H bonds from three specific C1-based CH_2_ groups (HCH) of **I–III** have so-called “local mode” vibrational behavior. Their C–H vibrations well follow Badger’s rule, so their C–H bond length is well proportional to their C–H stretch wavenumber. Thus, in this study, we combine the experimental data from neutron diffraction data and the vibrational spectroscopies and computations to study the C–H bond local mode by varying the force constants in Hooke’s law. Reported in in the last column of Table 1 is the calculated force constant data whose linear plot with calculated distance for **I–III** is shown in Figure S6A in SI will be discussed in detail in Section B2.

**Figure 12:**
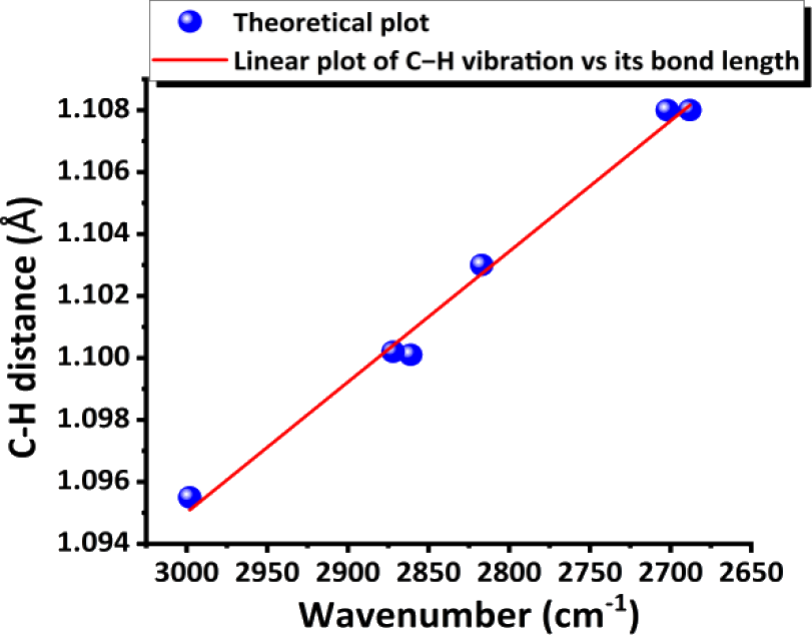
Theoretical linear plot of the C–H vibration wavenumber vs bond length for a local mode of complexes **I–III**; the equation is Y= −4.2096 × 10^–5^X + 1.2213 (eq 3). (Note: in **I**, when Y= 1.108 Å, X = 2691 cm^–1^.)

Furthermore, the C1-based neutron C–H bond lengths and MP2 computational data of **I–III** are summarized in Table 2. Taking entry 2 as an example, owing to the effective intramolecular TB, the combined elongation of two methylene C–H bonds is ≥ 16 mÅ. Thus, C1–H1B is elongated to 1.108(2) Å. The Gaussian computations confirm the elongated C1–H1A bond of 1.1068 Å obtained by MP2 calculation, which is then factored in, and the computed C1–H1A bond length becomes 1.1001 Å (entry 3). When other intermolecular HBs are considered, C1–H1A can further be shortened.^17^ Accordingly, the methylene C1–H1B bond in **II** is theoretically elongated to 1.1059 Å and determined to be ∼ 1.1080 Å. Additionally, the methylene C1–H1A bond in **III** (whose side-chain conformation is slightly different from that in **I** or **II**) is elongated and determined to be 1.1030 Å in length, and its C1–H1B bond length is calculated to be 1.0955 Å, which is known to become even shorter [to 1.093(1) Å shown in Figure 3] if the extra intermolecular blue-shifting HB of C1–H1B^···^I1 interaction is considered. In addition to the neutron structural data shown in Table 2, the C–H vibrational data were experimentally studied via vibrational spectroscopies (both FT-IR and Raman). As shown in Figure 5, the greatly red-shifted peak of the C1–H1B stretch in **I**, due to a significant C1^···^F2 TB interaction, then appears at 2688 cm^–1^, with increasing intensity in its red-shifted IR peak. Additionally, the vibrational (FT-IR and Raman) spectra of the (elongated) referenced (pivotal) lengths of C1–H1B from **4FCl-ZnI**_**2**_ **(II)** and C1–H1A from **2FCl-ZnI**_**2**_ **(III)** were also carefully assigned based on FT-IR data and confirmed by Raman spectroscopy data, as shown in Figure 6 and Figure S4, respectively.

#### B2 Local Vibrational Mode theory

Besides the local mode plots (Figures 9–11 and Tables 1&2), we also used local vibrational mode theory, which was developed by Kraka et al., to theoretically verify our results.^25–27^ A PyMOL software,^26^ which was developed by Kraka et al. via CNM (composition of normal mode) method^27^ to facilitate the local vibrational mode calculations, is then used to do the related calculations.^25–27^ By using PyMOL and based on local vibrational mode theory, the force constants for the specific CH_2_ group of all 3 complexes have been calculated and their data are shown in Table 3 which is a basically simplified table with the calculated force constant factored from Table 1 for easy side by side comparison and other related discussions in Section B3. It is found that all the calculated force constants are quite large values (>5 mdyn*/*Å). The relationship of C–H bond distance and the calculated force constant is then linear and can be plotted by using PyMOL software as Y = −34.7643·X + 43.5700 (eq 5) with R^2^= 99.7%. This linear plot based on the local vibrational mode theory and CNM method is shown in Figure 13. This is where the local force constant plays a crucial role. The relationship between the local force constant and the bond distance in Figure 13 suggests that the intrinsic strength of C–H bonds follows the normal correlation with the C–H bond distance in solid state. Thus, what we have observed the plot in Figure 13 is the normal relationship of BLBS.^24^ In other words, the more elongated the C–H bond, the weaker the C–H bond. Therefore, this plot with very good linearity clearly confirms the C–H bonds from three methylene C–H groups showing their C–H bond vibrational local mode theoretically.

**Table 3:** Calculated Force Constants for the Methylene C–H Bonds from Complexes **I–III**

**item no.**	**complexes**	C–H distance (Å)	**calculated force constant** (mdyn/Å)
1	**I**	1.1080	5.045
2	**I**	1.1001	5.317
3	**II**	1.1080	5.063
4	**II**	1.1002	5.314
5	**III**	1.0955	5.496
6	**III**	1.1030	5.226

**Figure 13:**
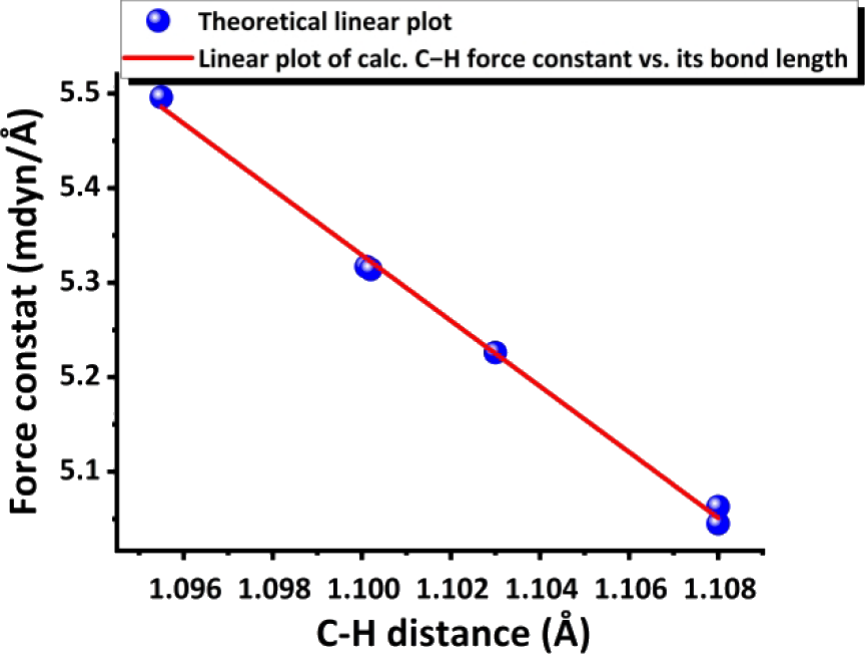
Linear plot of calculated sp^3^ C–H bond force constant vs its bond distance based on the local mode vibrational theory. Note: This plot along with experimental plot of Figure 7 is together plotted in Figure S6B in SI.

The CNM method is also used to dissect the normal modes of the methylene groups of complexes **I–III** into the local mode. Besides the normal DFT calculations, the DFT calculations (e.g., ωB97X-D/aug-cc-pVDZ) are also used for the suggested calculations.^27^ Take **I** for example, the plot of the (dissected) local mode vs normal mode of complex **I** is shown in Figure 14. The plots for **I–III** are all shown in Figure S7 which are also shown to be consistent with descriptions in Kraka’s paper.^27^ Based on the CNM method,^27e^ it clearly shows that the linear plots of C1–H1A and C1–H1B bonds in **I** show the local mode vibrational properties. As shown in Figure 14, the local mode of two C–H stretching frequencies with big difference in stretches is clearly seen from the left frequencies; and at the right side the normal mode region the vibrations of H1B–C1 and H1A-C1 are even farther apart due to the anharmonic factor (=0.975). The MP2 (MP2/LANL2DZ) method is used for the frequency calculations. The calculations of stretching frequencies using CNM approach from DFT method (ωB97X-D/aug-cc-pVDZ) have also been used. Both MP2 and DFT (ωB97X-D) methods show the same trend and similar results shown in Figure S7. The comparisons of CNM approach in studying the local mode stretching frequencies of complexes **I–III** (Figure S8) are shown in Figure S7 of SI.

**Figure 14:**
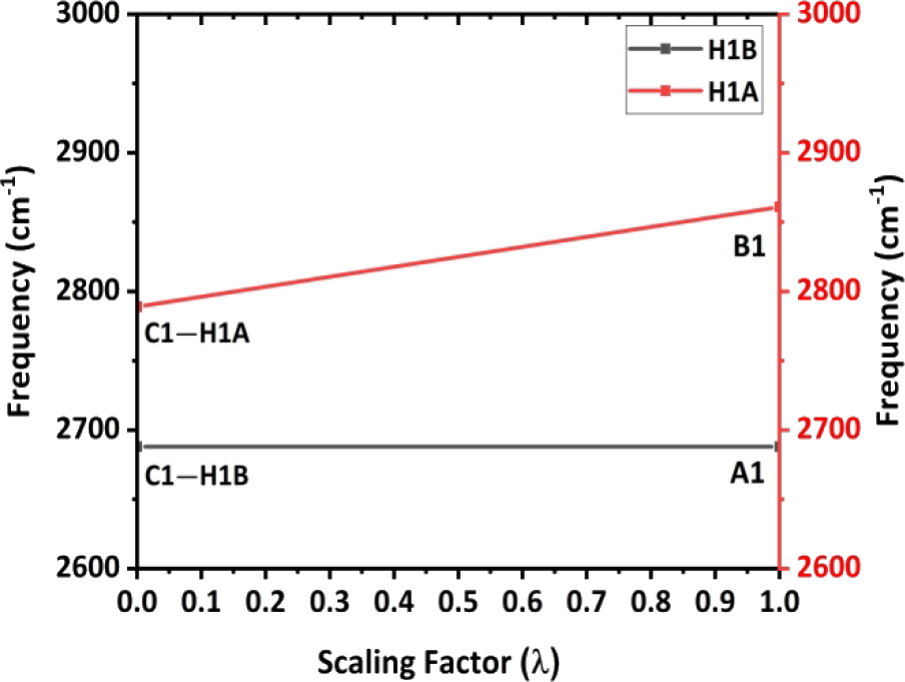
Using the CNM method (by MP2 method), the plot of the local mode vs the normal mode of complex **I**.

#### B3 Cremer-Kraka Criterion

As for complexes **II, III**, the same local mode trends have been also observed. Their plots are shown in Figure S7 in SI. In 2010, Kraka, Larsson, and Cremer (KLC)^31^ succeeded in generalizing the Badger’s rule from diatomic to polyatomic molecules utilizing the adiabatic internal coordinate mode (AICoMs) method to calculate local mode force constants. Using the Cremer-Kraka criterion which states that bond formation is associated with a gain in molecular energy. This gain is a result of a complex interplay of changes in the potential and kinetic energy. Thus, we also apply the local mode-based Cremer-Kraka criterion to study the property of blue-shifting HB. Based on the studies from Cremer^31^ et al., it is then found that *k*a values are shown in dependence of *R* (distance). Thus, the large *k*a values (>3 mdyn/Å) are found to be typical of covalent D-H bonds. [Note: D means a donor atom.]

In this study, by using PyMOL software, all the calculated local mode force constants are indeed >3 mdyn/Å (see Table 3), so three (item no. 2, 4, 5) intramolecular C–H^···^F blue-shifting (nonclassical) HBs from complexes (**I–III**) are then considered as the covalent HB (instead of electrostatic HB which is for classical HB). The blue-shifting HBs (e.g., C–H^···^F in **D**-H^···^ **A** HB; i.e., C and F are the donor and the acceptor atoms, respectively.) discussed in this paper is then the covalent HBs based on Cremer-Kraka criterion. As for TB, there are only limited data set (3 TBs). By using the suggested software, the calculated data, which are 0.160, 0.139, 0.178 mdyn/Å, seem to show these three TBs likely to be electrostatic nature.

#### B4. Other calculations

##### Gaussian Computations: NCI & NBO calculations

In addition to the above local mode calculations, Figure 15 shows that the noncovalent interaction (NCI) and natural bond orbital (NBO) analyses have been performed for the three Zn complexes (**I–III**) to further elucidate the presence of intramolecular TBs. The computational methods and basis sets are detailed in the SI. As shown in Figure 15a–c, the NCI plots clearly reveal the presence of TB interactions in complexes **I–III**. These interactions are highlighted by the red-circled green iso-surfaces located between C1···F2 in complexes **I** and **II**, and C1···F1 in complex **III** (in Figure 15a–c), unambiguously confirming the existence of intramolecular TBs. Furthermore, the NBO analyses shown in Figure 15d–f further affirms these findings, where the specific HOMO is mainly localized on the fluorine atom; and the LUMO is on the adjacent carbon atom, indicating the orbital overlapping which is consistent with TB interactions for complexes **I–III,** respectively.

**Figure 15:**
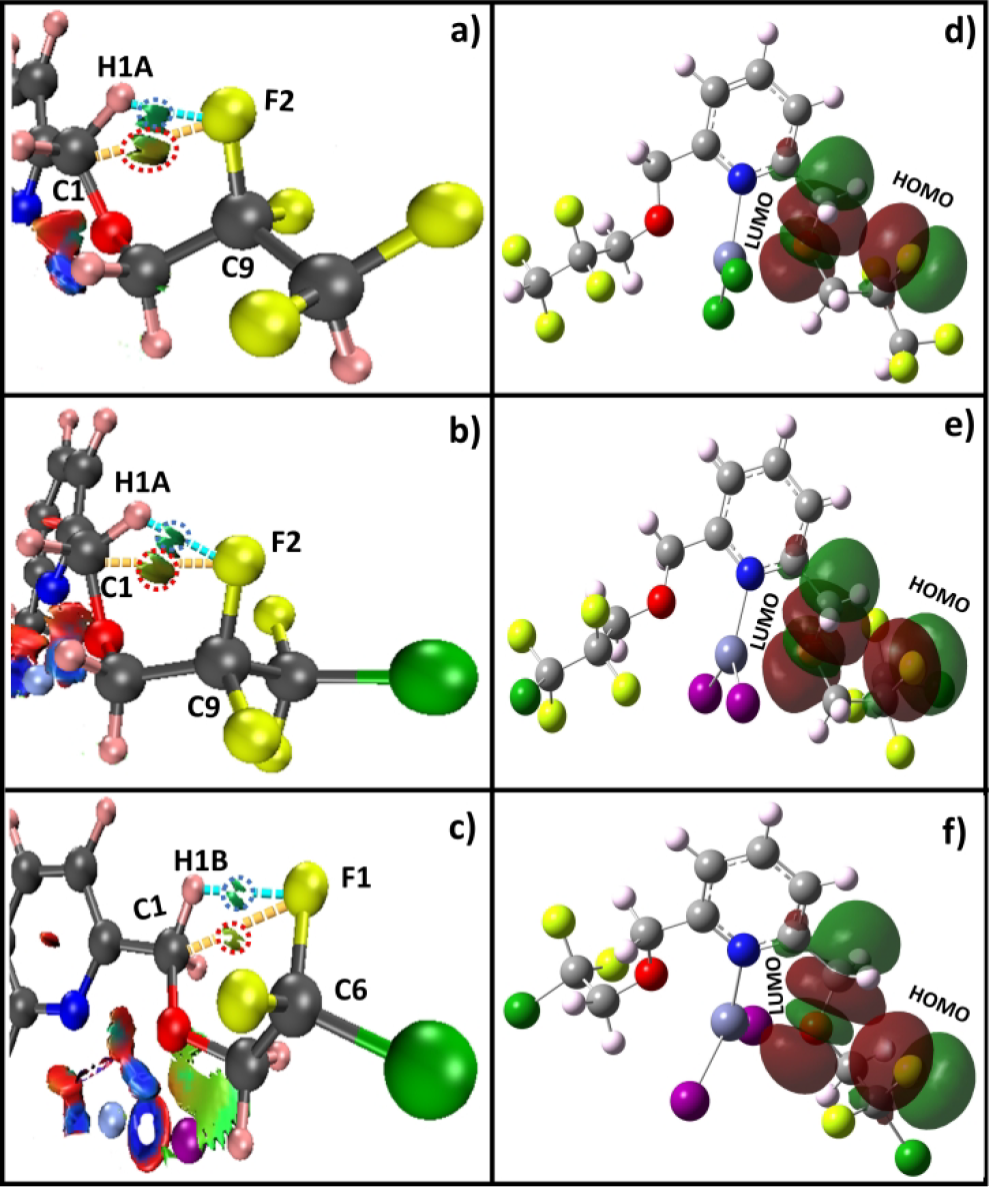
a-c) NCI analyses and d-f) NBO analyses of complexes (**I–III**) showing the presences of the intramolecular TBs, respectively.

## Conclusions

In summary, three pincered Zn complexes were successfully synthesized and characterized, and their neutron structures were determined. This type of pincered complex shows a unique 6-membered cyclohexane-like form with a model with which an intramolecular TB and improper HBs can be systematically studied. This model features a change in the C–H bond length, which can be well studied by using high-resolution neutron diffraction. The intramolecular TB lengths in **I** and **II** are ≤ 2.956 Å, and those in **III** are ∼ 3.120 Å. Thus, the intramolecular TB-induced combined C–H bond elongations in **I** and **II** and in **III** are ∼ 16 and 10 mÅ, respectively. The combined C1–H1 bond elongation in **I** and **II** of ∼ 16 mÅ is unprecedented, and the C1–H1B bond in the methylene group of **I** being 16(2.4) mÅ longer than its C1–H1A bond is astonishing. In contrast, in the intermolecular case of a recent paper,^17^ two C–H bonds differed in length by only 9(4.2) mÅ. Thus, when C1–H1A and C1–H1B from **I–III** differ in bond length by ≥ 10 mÅ, two methylene C1–H1 stretches from **I–III** vibrate via a local mode (also see SI A) and obey Hooke’s law.^15^ This neutron-based study is believed to prove Badger’s rule, which is known to describe the (nonlinear) dependence of the harmonic bond force constant on the bond length. For **4FH-ZnCl**_**2**_(**I**) in particular, the C1–H1A and C1–H1B bonds differ in length by 16 mÅ such that either the C1–H1A or C1–H1B bond behaves like a specifically independent methine C–H group whose vibration can be simply predicted by Hooke’s law. For the first time, the peak of the sp^3^ C–H bond (i.e., C1–H1B), whose length is significantly elongated due to an intramolecular TB, is greatly red-shifted to 2688 cm^–1^. This 2688 cm^–1^ peak is significantly more red-shifted than its other geminal C–H peak (= 3020 cm^–1^). Thus, the most important findings in this study are as follows: 1. Two C–H bond lengths of **I–III** have been found to well correlate with their respective bond force constants via a local mode. 2. Based on Hooke’s law, for the first time, a local mode has been achieved simply by tuning, in a methylene group, two C–H bond lengths that are well proportional to the bond force constants. 3. The intramolecular TB, within a 6-membered chair form, easily induces significant elongation of one C1–H1 bond in the H–C–H group of **4FH-ZnCl**_**2**_ by 8 mÅ such that the two methylene C–H bonds greatly differ in length by 16 mÅ. 4. Owing to the local mode, the C–H stretch of an elongated sp^3^ C1–H1B bond in **I** is so red-shifted that it appears at **2688** cm^–1^, which makes C1–H1B bond being 332 cm^–1^ more red-shifted in the stretch than the geminal C1–H1A bond, is unprecedented. Furthermore, before this study, scientists mainly used the reduced mass difference to reach a local mode of C–H bonding (via deuteration). However, the reduced mass (μ) is almost unrelated to the force constant in Hooke’s law. To our knowledge, this is the only report to use accurate high-resolution neutron C–H bond length data obtained by varying the C–H bond lengths in a specific CH_2_ group via an intramolecular TB and intramolecular blueshifting HB(s). Thus, for the first time, experimental and theoretical tuning of the k (force constant) in the numerator in Hooke’s law has been correctly achieved and simply predicted by varying two methylene C–H bond lengths to reach a local mode. Finally, Badger’s rule, has been experimentally verified via neutron studies, deuterations and vibrational spectroscopic studies of all three complexes.

The fundamental vibrations of molecules are normally delocalized over whole molecules rather than only being localized at specific chemical bonds because of both electronic and mass-based couplings. McKean could isolate some C–H (or O–H) stretching vibrations via isotopic substitution by varying the mass-related terms in the denominator of Hooke’s law. As reported here, we can experimentally and theoretically vary the bond strengths (or bond force constants) in the numerator of Hooke’s law to reach a local mode. In other words, Badger’s rule, which was extended by KLC in 2010, can also be extended by using neutron and spectroscopic studies and reconfirmed for even in a H–C–H triatomic hydrocarbon system.

## Supplementary Material



## Notes

SI A contains synthesis details, characterization data, single-crystal X-ray/neutron methods, other crystallographic data, other related data and computational information (PDF)

## ABBREVIATIONS

HB: hydrogen bond
vdW: van der Waals
TBs: tetrel bonds
NCIs: noncovalent interactions.
