# Correction to “Neutron Diffraction and Spectroscopic Studies of Intramolecular Tetrel Bonds in Three Fluorinated Zinc Complexes: Significant Redshift in the sp^3^ C–H Stretch Confirmed by Experiments and Theory”

**DOI:** 10.1021/jacs.5c22553

**Published:** 2026-01-14

**Authors:** Norman Lu, Gurumallappa Gurumallappa, Pin-Yu Liu, Ka-Long Chan, Yu-Cheng Huang, Yu-Ching Lin, Yun-Ting Hsieh, Pin-Xiang Zeng, Yashwanth Gowda, Meng-Hsun Tsai, Eskedar Tessema, Huan-Cheng Chang, Joseph S. Francisco

In Table 2, the column headings in the top row were misaligned and have been corrected by moving the wordings one column to the right.

**Table 2:** Calculated C–H Bond Lengths of Monomeric **I–III** and Elongated C–H Bond Lengths from the Experimental Neutron Data of **I–III** ^a^ ^,^ ^b^ ^,^ ^c^

**#**		**C–H bond length**	**MP2 (monomer) (Å)**	**neutron data (Å)**	**comments**
1	**I**	C1–H1A	1.1011	<1.1011^d^	with C1–H1A···F8 & C1–1A···F2 intermolecular HBs
2		C1–H1B	(1.1080)	1.108(2)	
3	**II**	C1–H1A	1.1001	<1.100^d^	with C1–H1···I2 & C1–H1A···F3 intermolecular HBs
4		C1–H1B	(1.1080)	1.108(2)	
5	**III**	C1–H1A	(1.1030)	1.103(2)	
6		C1–H1B	1.0955	<1.0955^d^	with C1–H1B···I1 intermolecular HB

a C1–H1A in **I** and **II** and C1–H1B in **III** are shortened (see SI).

b Two methylene C–H bond lengths are affected mainly by the intramolecular TB and improper HBs, so the shortened C–H bond discussed here is mainly considered to involve significant intramolecular interactions.

c The MP2 data reported here are factored data. Before factoring, the calculated values are (1.1068, 1.0989), (1.1059, 1.0986) and (1.1066, 1.0991) Å for **I–III**, listed in the calc. length column in Table 1.

d When one or more improper HB is added,^17^ the C1–H bond becomes even shorter.

The Supporting Information has been replaced with a corrected version. The findings of the manuscript are not affected by the correction changes.

## Supplementary Material



## Notes

Synthesis details, characterization data, single-crystal X-ray and neutron diffraction methods, crystallographic data, other deuterated data, and computational details of sections B1–B4, including (i) quantum-chemical calculations performed using MP2 method by the Gaussian 16 program, (ii) PyMOL software & CNM approach, (iii) Cremer-Kraka criterion, and (iv) NCI and NBO analyses, respectively (PDF)

