# Supplementary material for: Neutron Diffraction and Spectroscopic Studies of Intramolecular Tetrel Bonds in Three Fluorinated Zinc Complexes: Significant Redshift in the sp3 C–H Stretch Confirmed by Experiments and Theory
Source: J Am Chem Soc. 2025 Nov 26;147(49):45270–82. doi: 10.1021/jacs.5c15040 (PMC12703683; doi:10.1021/jacs.5c15040)
Supplement: Supplementary file 1 [file ja5c15040_si_001.pdf]

# Neutron Diffraction and Spectroscopic Studies of Intramolecular Tetrel Bonds in Three Fluorinated Zinc Complexes: Significant Redshift in the sp$^3$ C–H Stretch Confirmed by Experiments and Theory

Norman Lu,[1,2]* Gurumallappa Gurumallappa,[1,2] Pin-Yu Liu,[1] Ka-Long Chan,[1] Yu-Cheng Huang,[1] Yu-Ching Lin,[1] Yung-Ting Hsieh,[1] Pin-Xiang Zeng,[1] Yashwanth Gowda,[1] Meng-Hsun Tsai, Eskedar Tessema,[1] Huan-Cheng Chang,[3] Joseph S. Francisco[4]*

Institute of Organic and Polymeric Materials, National Taipei University of Technology, Taipei 106, Taiwan (ROC). E-mail: normanlu@mail.ntut.edu.tw

Graduate Institute of Energy and Optoelectronic Materials, National Taipei University of Technology, Taipei 106, Taiwan (ROC).

Institute of Atomic and Molecular Sciences, Academia Sinica, Taipei 106, Taiwan (ROC).

Department of Earth and Environmental Science and Department of Chemistry, University of Pennsylvania, Philadelphia, Pennsylvania 19104-6316, United States. E-mail: frjoseph@sas.upenn.edu

## Table of Contents

### I. Experimental procedures

Single crystal X-ray diffraction studies ..... SI 4
Single crystal neutron diffraction studies ..... SI 4
Computational methods ..... SI 5

### II. Supporting scheme

Scheme S1. Schematic representation of tetrel bond and C-H···F improper H-bond ..... SI 6
Scheme S2. The image of a cyclohexane-like chair form ..... SI 6
Scheme S3. The Schematic representation of Hooke's Law ..... SI 7

### III. Supporting figures of (A) neutron structures and (B) the vibrational spectra of two methylene C-H bonds under local mode

Figure S1. ORTEP diagram of Neutron structure of complex **4FH-ZnCl$_2$(I)** ..... SI 8
Figure S2. FT-IR spectrum of **4FH-ZnCl$_2$(I)** in the region of [3100, 2600] cm$^{-1}$ ..... SI 9
Figure S3. ORTEP diagram of Neutron structure of complex **4FCl-ZnI$_2$(II)** ..... SI 9
Figure S4. ORTEP diagram of Neutron structure of complex **2FCl-ZnI$_2$(III)** ..... SI 10
Figure S5. The linear plot of C-H vibration vs its bond length **without pivotal points** for complexes (**I-III**) ..... SI 11

### IV. Structural table section

Table S1. Crystallographic data refinement parameters table for **4FH-ZnCl$_2$(I)** ..... SI 12
Table S2. Weak interactions (w/o libration correction) in **4FH-ZnCl$_2$(I)** ..... SI 13
Table S3. Weak interactions (with libration correction) in **4FH-ZnCl$_2$(I)** ..... SI 13
Table S4. Selected bond lengths and angles (w/o libration correction) for **4FH-ZnCl$_2$(I)** ..... SI 14
Table S5. Selected bond lengths and angles (with libration correction) for **4FH-ZnCl$_2$(I)** ..... SI 15
Table S6. Crystallographic data refinement parameters table for **4FCl-ZnI$_2$(II)** ..... SI 16
Table S7. Weak interactions of tetrel bond and improper HB in **4FCl-ZnI$_2$(II)** ..... SI 17
Table S8. Selected bond lengths and angles for **4FCl-ZnI$_2$(II)** ..... SI 18
Table S9. Crystallographic data refinement parameters table for **2FCl-ZnI$_2$(III)** ..... SI 19
Table S10. Weak interactions (w/o libration correction) in **2FCl-ZnI$_2$(III)** ..... SI 20
Table S11. Weak interactions of (with libration correction) in **2FCl-ZnI$_2$(III)** ..... SI 20
Table S12. Selected bond lengths and angles (w/o libration correction) for **2FCl-ZnI$_2$(III)** ..... SI 21
Table S13. Selected bond lengths and angles (with libration correction) for **2FCl-ZnI$_2$(III)** ..... SI 21

Table S14. Experimental data of the wavenumber vs. neutron C–H bond length ........ SI 22
Table S15-23. Atomic coordinates of complexes (**I-III**) ........ SI 23

**Other related experimental data**

**A.** Synthesis of deuterated **4FH-ZnCl$_2$** and related compounds ........ SI 32

**V. NMR spectra of complexes (I-III)** ........ SI 34

**VI. References** ........ SI 39

## I. Experimental procedures

### Single Crystal X-ray Diffraction Studies

Data were collected using a Rigaku XtaLAB Synergy DW single crystal diffractometer equipped with a HyPix-Arc 150° curved Hybrid Photon Counting X-ray detector and MicroMax-007 HF microfocus rotating anode with dual wavelength (Cu and Mo). Data processing was carried out using the Bruker software package, and structure solution and refinement were carried out with the SHELXS routines. All H atoms were calculated and treated with a riding model. The H atom isotropic displacement parameters were defined as 1.2 Ueq of the adjacent atom.

### Single Crystal Neutron Diffraction Studies of Complex (I), (III).

Single crystal neutron diffraction data for fluorinated [(**4FH-ZnCl$_2$**(**I**) and **2FCl-ZnI$_2$**(**III**) metal complexes were determined on the TOPAZ single−crystal time−of−flight (TOF) Laue diffractometer at the Spallation Neutron Source, Oak Ridge National Laboratory (Tennessee, USA).[1-3] The diffractometer had 18 detectors installed, each with an active area of 15 cm x 15 cm, and they were arranged on a near−spherical detector array tank. The initial moderator−to−sample flight path was 18 m, and the sample−to−detector distances varied in the range 39−46 cm. The total path length of 18.4 m and the SNS pulse rate of 60 Hz provided a wavelength bandwidth of 3.6 Å. Each crystal of complexes (**I, III**), with the dimension, [2.40×1.80×1.60 (**I**), 2.25 × 1.55 × 1.50 (**III**) specified in its neutron cif, was mounted on the tip of a MiTeGen loop using Super Glue and cooled to 100 K for data collection. Total of 40 crystal orientations optimized with CrystalPlan[4] software were used to ensure better than 95% coverage of a hemisphere of reciprocal space. Each orientation was measured for 6 − 7 coulombs of proton−charge for approximately 1.5 h with the SNS operated at 1.4 MW. The integrated raw Bragg intensities were obtained using 3−D ellipsoidal Q−space integration in accordance with previously reported methods.[5] Data reduction, including the neutron TOF spectrum, Lorentz and detector efficiency corrections, was carried out with the ANVRED3 program.[6] A Gaussian numerical absorption correction was applied with $\mu = 0.11327 + 0.07311\lambda$ mm$^{-1}$. The reduced data were saved in SHELX HKLF2 format, where the wavelength was recorded separately for each individual reflection and not merged. Non-hydrogen atom positions from the X−ray structure were used as the starting model for the refinement of the neutron structure. Refinement was performed using the SHELXL−2018/3 program interfaced with ShelXle.[6,7] Hydrogen atoms were located from the difference Fourier map and refined anisotropically to convergence.

### Single Crystal Neutron Diffraction Studies of Complex (II).

Single crystal neutron diffraction data for fluorinated **4FCl-ZnI$_2$**(**II**) metal complex was taken using SENJU single−crystal time−of−flight (TOF) Laue neutron diffractometer[8] installed at BL18 at the Materials and Life Science Experimental Facility (MLF) of Japan Proton Accelerator Research Complex (J-PARC) Japan. The wavelength range of incident neutrons was 0.4 to 4.4 Å. A block−shaped crystal of complex (**II**), with dimensions of 2.50 mm × 2.50 mm × 1.00 mm, was mounted on the top of an aluminum pin using an epoxy glue and attached to a fixed-$\chi$ type two-axes ($\omega$, $\varphi$) goniometer. Then, the diffraction data was acquired at 40 K under vacuum conditions.

Intensities of Bragg peaks were collected using 41 two-dimensional scintillation detectors with 24 crystal orientations. The exposure time for one crystal orientation was 3 hours. The accelerator power was 800 kW. Data reduction was performed using the STARGazer[9,10] to obtain hkl indexes and the corresponding integrated intensities of reflections corrected for the detector efficiency, Lorentz factor, and scaling factor for each crystal orientation. A spherical absorption correction was applied with $\mu = 0.198 + 0.00874\lambda$ $mm^{-1}$. The reduced data were saved in SHELX HKLF2 format, where the wavelength was recorded separately for each individual reflection. Non-hydrogen atom positions from the X–ray structure were used as the starting model for the refinement of the neutron structure. Refinement was performed using the SHELXL–2018/3 program interfaced with ShelXle.[6,7] Hydrogen atoms were located from the difference Fourier map and refined anisotropically to convergence.

**Notes**

Deposition numbers 2351846 [4FH-$ZnCl_2$(**I**)], 2351847 [4FCl-$ZnI_2$(**II**)] and 2351848 [2FCl-$ZnI_2$(**III**)] contain the supplementary crystallographic x-ray data for this paper. The neutron diffraction data (without libration correction) for complexes (**I**), (**II**) and (**III**), are provided as 2352173, 2352174 and 2352175, respectively. The neutron diffraction data (with libration correction) for complexes (**I**) and (**III**) are provided as 2352217 and 2352218, respectively. These data are provided free of charge by the joint Cambridge Crystallographic Data Center.

## Computation Method

The effects of libration on the molecular geometry of the complexes (**I**) and (**III**) were corrected using the THMA14c program[11-13] in WinGX.[14] The neutron diffraction data obtained at 40 K for complex (**II**) has been directly for further analysis. All quantum-chemical calculations were performed using MP2[15] level of theory by the Gaussian 16[16] program and output files are visualized by its GaussView 6.0.16 program.[17] Basis sets of LANL2DZ[18] and 6–31G(d,p)[,19] were utilized to describe I atom and main group elements (such as H, C, N, O, F, Cl, and Zn atoms), respectively. Some calculated C1-H1 bond distances by MP2 are slightly factored.

Note 1. Natural bond orbital (NBO) and noncovalent interaction (NCI) calculations are performed using the Gaussian 16 program and the results are provided in the main content.

Note 2. In addition to the MP2/6-31G(d,p) level of theory, the vibrational calculations for complex (I) are performed using the MP2/LANL2DZ method. The results are consistent with the data obtained from the MP2/6-31G(d,p) calculations.

Note 3. All local mode calculations for complexes (**I–III**) were verified by analyzing their vibrational spectra using Gaussian 16 to validate the computational model. The simulated IR spectra align with experimental data, and all optimized structures exhibit only positive vibrational frequencies, confirming their stability. Additionally, the calculated IR frequencies closely match the experimental values.

## II. Supporting scheme

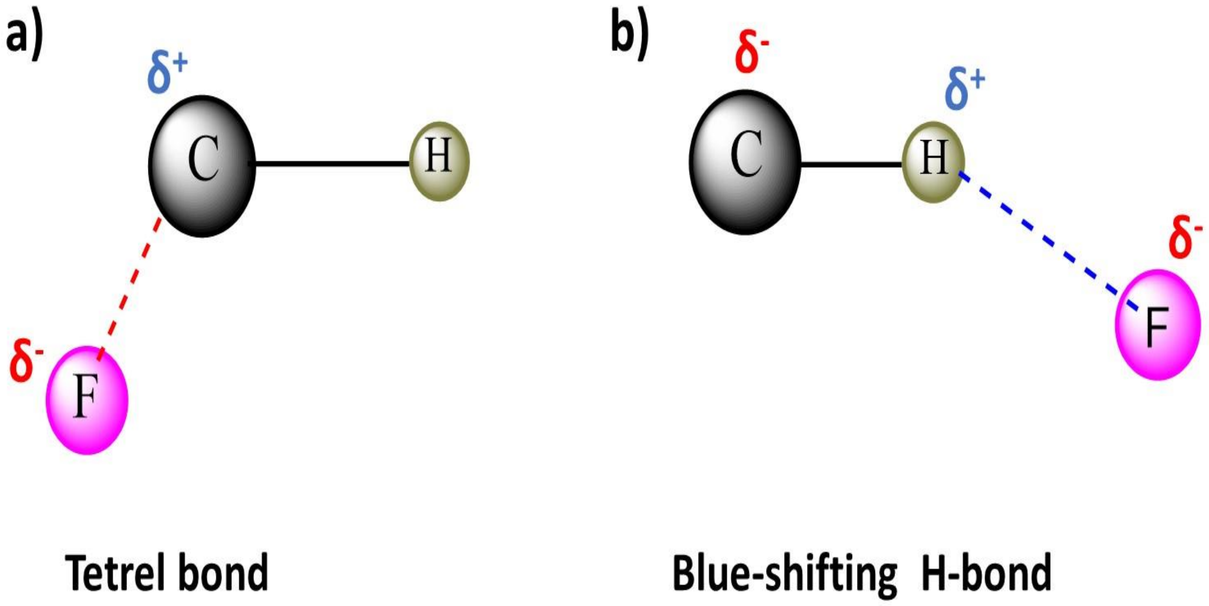


**Scheme S1.** Schematic representation of a) tetrel bond (TB) and b) C-H⋯F improper H-bond (HB).

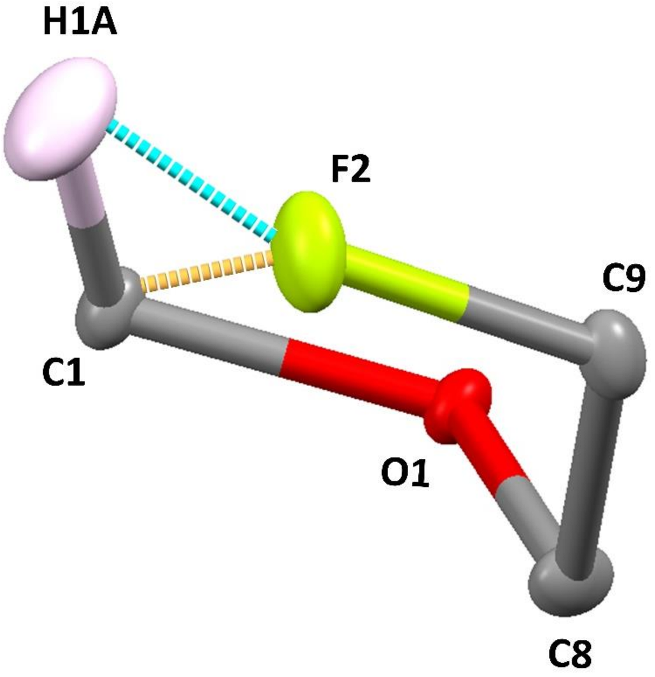


**Scheme S2.** The image of a cyclohexane-like chair form.

[**Note.** The intramolecular (C1⋯F2) TB, within a 6-membered chair form.]

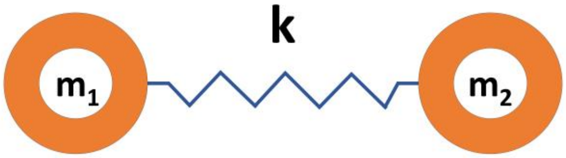


$$\overline{\nu} = \frac{1}{2\pi c}\sqrt{\frac{k}{\mu}} \quad \text{..... eq (s1)}$$

$$\mu = \frac{m_1 \, . \, m_1}{m_1 + m_1} \quad \text{..... eq (s2)}$$

**Scheme S3.** The Schematic representation of Hooke's Law.

(Note. The wavenumber equation and reduced mass expression are shown.)

### III. A. Supporting figures of neutron structure

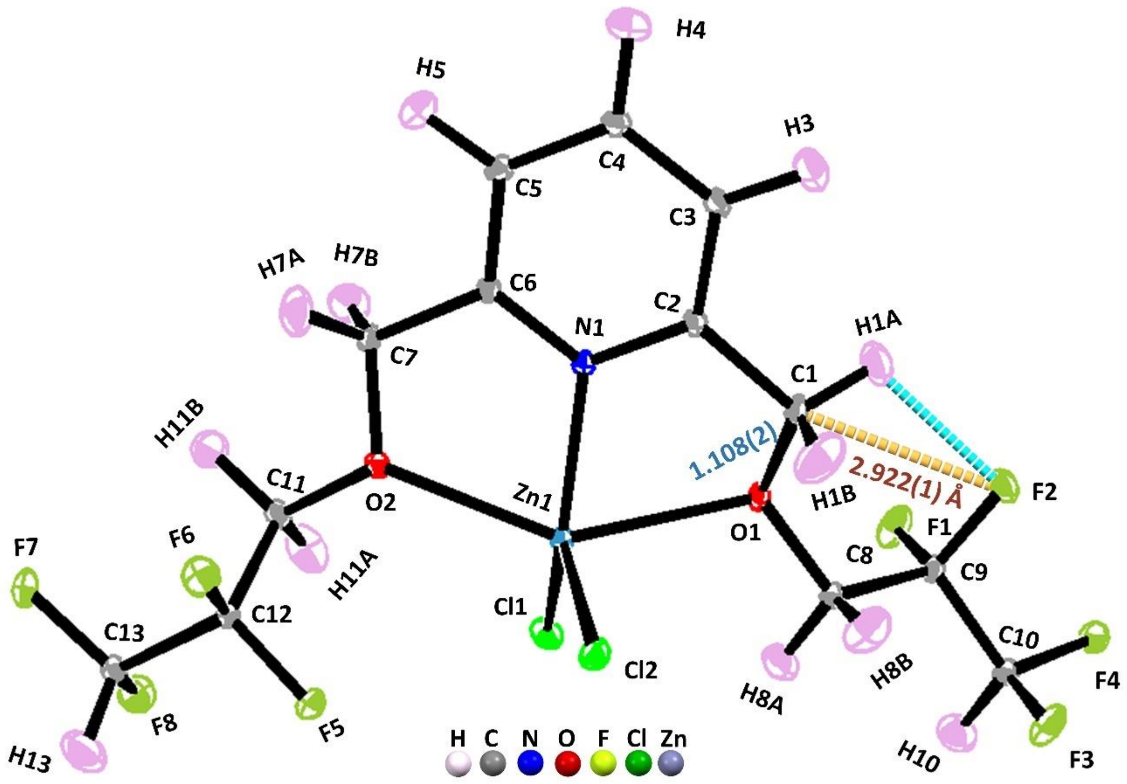


**Figure S1.** ORTEP of **4FH-ZnCl$_2$(I)** neutron structure and its drawing shows the intramolecular TB. [d(C⋯F)=2.922(1) Å, angle:150.5°; C1-H1B=1.108(2) and C1-H1A=1.092(1) Å].

## B. Supporting vibrational spectrum

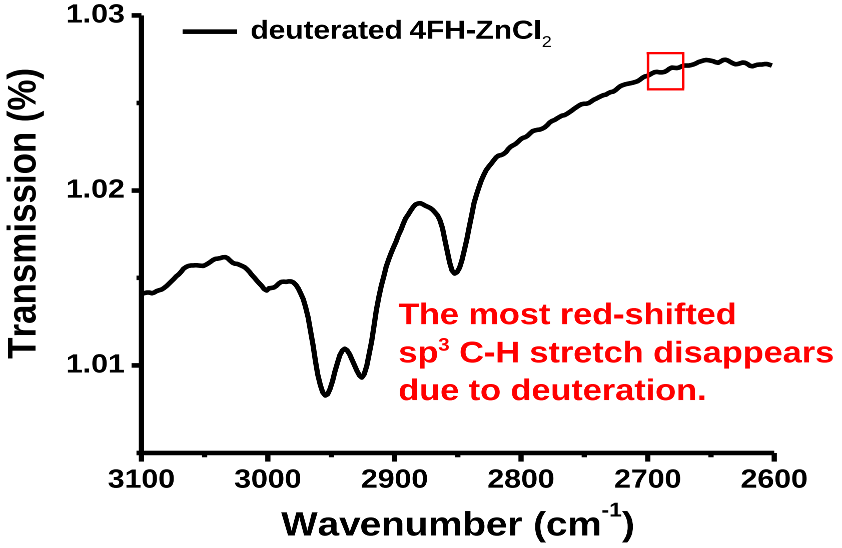


**Figure S2.** FT-IR spectrum of **4FH-ZnCl**$_2$(**I**), which shows the most red-shifted *sp*$^3$ C1-H1B bond of 1.108(2) Å; and its IR appears at 2688 cm$^{-1}$.

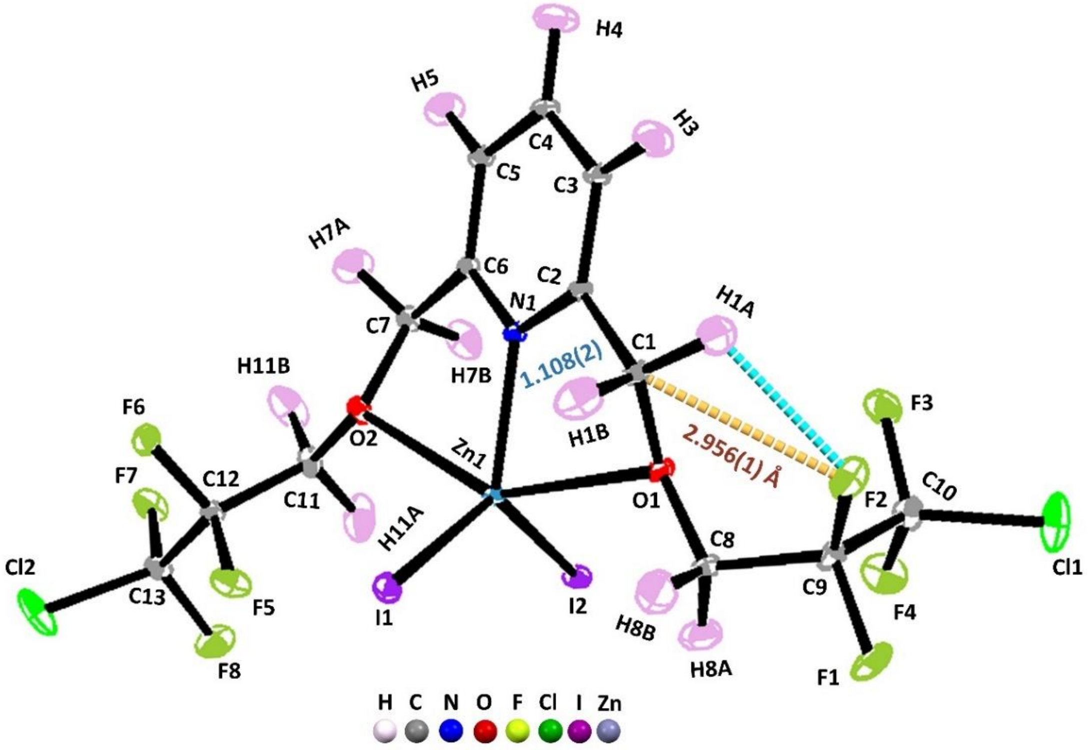


**Figure S3.** ORTEP of **4FCl-ZnI**$_2$(**II**) neutron structure and its drawing shows the intramolecular TB. [d(C···F)=2.956(1) Å, angle:153.2°; C1-H1B=1.108(2) and C1-H1A=1.095(4) Å]. [Note. This structure was measured in J-PARC at 40 K in Japan.]

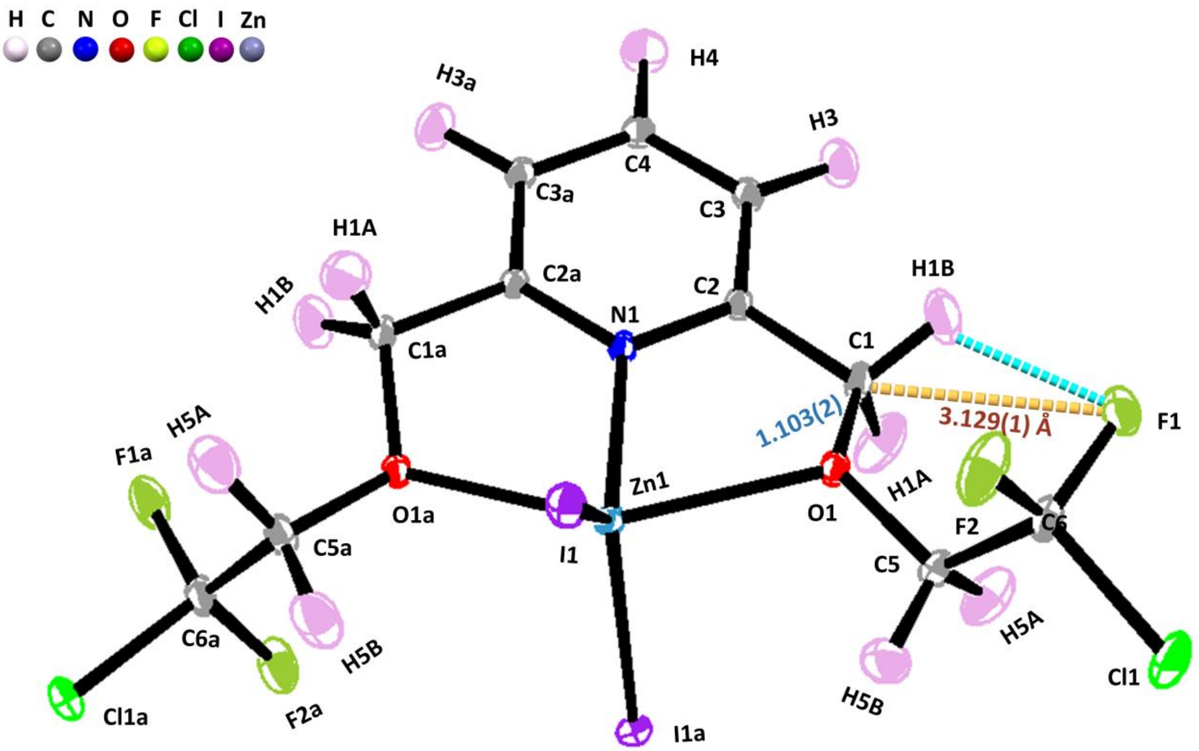


**Figure S4.** ORTEP of **2FCl-ZnI$_2$(III)** neutron structure with the intramolecular TB indicated. Note. The drawing of **2FCl-ZnI$_2$(III)** shows the intramolecular TB, [d(C···F)=3.129(1) Å, angle:143.8°; C1-H1A=1.103(2) and C1-H1B=1.093(1) Å].

**Local mode of 2FCl-$ZnI_2$(III) complex**

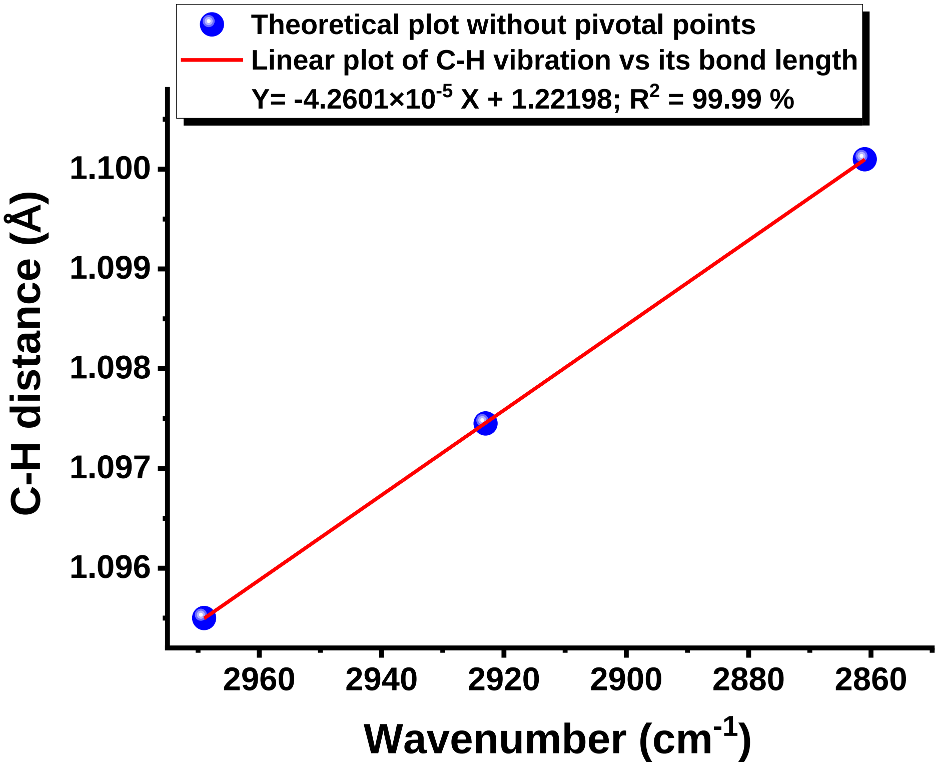


**Figure S5.** The theoretical linear plot of C-H vibration vs its bond length without pivotal points for complexes **I-III**.

## IV. Structural table section

**Table S1**. Crystallographic data and refinement parameters for **4FH-ZnCl₂(I)** complex.

| | **X-ray diffraction CCDC ID: 2351846** | **Neutron diffraction CCDC ID: 235221** |
|---|---|---|
| Crystal data | | |
| Chemical formula | $C_{13}H_{13}Cl_2F_8NO_2Zn$ | $C_{13}H_{13}Cl_2F_8NO_2Zn$ |
| $M_r$ | 503.51 | 503.51 |
| Crystal system, space group | Triclinic, $P\bar{1}$ | Triclinic, $P\bar{1}$ |
| Temperature (K) | 150 | 100 |
| *a*, *b*, *c* (Å) | 8.1118 (5), 11.1532 (6), 11.7262 (7) | 8.0922 (3), 11.1333 (3), 11.6641 (3) |
| α, β, γ (°) | 113.180 (1), 107.810 (2), 98.124 (2) | 113.041 (2), 107.686 (3), 98.282 (3) |
| $V$ (Å$^3$) | 886.03 (9) | 878.90 (5) |
| $Z$ | 2 | 2 |
| Radiation type | Mo *K*a | Neutron, l = 0.800 Å |
| m (mm$^{-1}$) | 1.78 | 0.1622 + 0.07163λ |
| Crystal size (mm) | 0.75 × 0.63 × 0.51 | 2.40 × 1.80 × 1.60 |
| | | |
| Data collection | | |
| Diffractometer | XtaLAB Synergy R, DW system, HyPix-Arc 150 | TOPAZ |
| Absorption correction | Multi-scan[a] | Gaussian[b] |
| $T_{min}$, $T_{max}$ | 0.349, 0.464 | 0.505, 0.771 |
| No. of measured, independent and observed [$I$ > 2s($I$)] reflections | 24880, 3164, 3036 | 34267, 8113, 7369 |
| $R_{int}$ | 0.042 | 0.076 |
| (sin q/l)$_{max}$ (Å$^{-1}$) | 0.601 | 1.225 |
| | | |
| Refinement | | |
| $R[F^2 > 2s(F^2)]$, $wR(F^2)$, $S$ | 0.024, 0.061, 1.08 | 0.029, 0.065, 1.01 |
| No. of reflections | 1769 | 8113 |
| No. of parameters | 107 | 362 |
| H-atom treatment | H-atom parameters constrained | All H-atom parameters refined |
| $\Delta\rho_{max}$, $\Delta\rho_{min}$ | 0.64, -0.81(e Å$^{-3}$) | 0.90, -0.91(fm Å$^{-3}$) |

a) Bruker SADABS, 1996. (b) P. Coppens, L. Leiserowitz, D. Rabinovich, Acta Cryst. 18, 1035-1038 (1965).

**Table S2.** Weak interactions of tetrel bond and improper HB in **4FH-$ZnCl_2$(I)** complex [Å and °] (without libration correction).

| Item no | a) Tetrel bond (O−C···F−C) in 4FH-$ZnCl_2$(I) | | | | |
|---|---|---|---|---|---|
| | **H/C—C···F—C** | **H/C—C** | **C···F** | **H/C···F or C···C** | **∠CFC** |
| 1 | C2−C1···F2−C9 | C2−C1= 1.506(1)<br>F2−C9 = 1.354(1) | C1···F2=2.922(1) | C2···F2= 4.297(1)<br>C1···C9= 3.135(1) | ∠C2C1F2= 150.5(0)<br>∠C9F2C1= 86.1(0) |
| | **b) Improper hydrogen bonds (C−H···*A*)** | | | | |
| | ***D*—H···*A*** | ***D*—H** | **H···*A*** | ***D*···*A*** | **∠*D*H*A*** |
| 2 | C1—H1A···F2 | 1.091(1) | 2.323(2) | 2.922(1) | 112.6(1) |

**Table S3.** Weak interactions of tetrel bond and improper HB in libration-corrected **4FH-$ZnCl_2$(I)** complex [Å and °].

| Item no | a) Tetrel bond (O−C···F−C) in 4FH-$ZnCl_2$(I) | | | | |
|---|---|---|---|---|---|
| | **H/C—C···F—C** | **H/C—C** | **C···F** | **H/C···F or C···C** | **∠CFC** |
| 1 | C2−C1···F2−C9 | C2−C1= 1.507(1)<br>F2−C9 = 1.355(1) | C1···F2=2.922(1) | C2···F2= 4.298(1)<br>C1···C9= 3.135(1) | ∠C2C1F2= 150.5(0)<br>∠C9F2C1= 86.1(0) |
| | **b) Improper hydrogen bonds (C−H···*A*)** | | | | |
| | ***D*—H···*A*** | ***D*—H** | **H···*A*** | ***D*···*A*** | **∠*D*H*A*** |
| 2 | C1—H1A···F2 | 1.092(1) | 2.323(2) | 2.922(1) | 112.6(1) |

**Table S4.** Selected bond lengths (Å ) and bond angles (°) for monomeric **4FH-$ZnCl_2$(I)** complex (without libration correction).

| **Bond length (Å)** | | | |
|---|---|---|---|
| **Bond** | **Bond length (Å)** | **Bond** | **Bond length (Å)** |
| F5—C12 | 1.363(1) | C8—H8A | 1.098(1) |
| F6—C12 | 1.354(1) | C3—H3 | 1.085(1) |
| F4—C10 | 1.346(1) | C7—H7A | 1.093(1) |
| F8—C13 | 1.351(1) | C7—H7B | 1.095(1) |
| F2—C9 | 1.354(1) | C1—H1A | 1.091(1) |
| F7—C13 | 1.356(1) | C1—H1B | 1.106(1) |
| F1—C9 | 1.356(1) | C10—H10 | 1.094(1) |
| F3—C10 | 1.347(1) | C11—H11A | 1.095(1) |
| C4—H4 | 1.089(1) | C11—H11B | 1.098(1) |
| C5—H5 | 1.090(1) | C13—H13 | 1.092(1) |
| C8—H8B | 1.095(1) | | |
| **Bond angle (°)** | | | |
| **Bond** | **Bond angle (°)** | **Bond** | **Bond angle (°)** |
| H3—C3—C2 | 120.1(1) | H8A—C8—O1 | 107.0(1) |
| H3—C3—C4 | 121.2(1) | H8B—C8—C9 | 108.8(1) |
| H7A—C7—H7B | 108.4(1) | H8A—C8—C9 | 108.4(1) |
| H7A—C7—O2 | 110.3(1) | H1A—C1—C2 | 109.7(1) |
| H7B—C7—O2 | 109.6(1) | H1B—C1—C2 | 109.8(1) |
| H7A—C7—C6 | 110.2(1) | H10—C10—F4 | 109.9(1) |
| H7B—C7—C6 | 109.6(1) | H10—C10—F3 | 109.0(1) |
| H1A—C1—H1B | 108.6(1) | H10—C10—C9 | 111.9(1) |
| H1A—C1—O1 | 111.4(1) | H11A—C11—H11B | 109.8(1) |
| H1B—C1—O1 | 109.2(1) | H11A—C11—O2 | 109.6(1) |
| H4—C4—C5 | 119.8(1) | H11B—C11—O2 | 110.8(1) |
| H4—C4—C3 | 120.8(1) | H11A—C11—C12 | 109.9(1) |
| H5—C5—C4 | 121.4(1) | H11B—C11—C12 | 108.6(1) |
| H5—C5—C6 | 120.0(1) | H13—C13—F8 | 110.2(1) |
| H8B—C8—H8A | 109.6(1) | H13—C13—F7 | 109.6(1) |
| H8B—C8—O1 | 111.2(1) | H13—C13—C12 | 112.5(1) |

**Table S5.** Selected bond lengths (Å ) and bond angles (°) for libration corrected monomeric **4FH-ZnCl$_2$(I)** complex.

| **Bond length (Å)** | | | |
|---|---|---|---|
| **Bond** | **Bond length (Å)** | **Bond** | **Bond length (Å)** |
| F1—C9 | 1.357(1) | C4—H4 | 1.090(1) |
| F2—C9 | 1.355(1) | C5—H5 | 1.090(1) |
| F3—C10 | 1.348(1) | C7—H7A | 1.094(1) |
| F4—C10 | 1.347(1) | C7—H7B | 1.096(1) |
| F5—C12 | 1.364(1) | C8—H8A | 1.099(1) |
| F6—C12 | 1.354(1) | C8—H8B | 1.095(2) |
| F7—C13 | 1.358(1) | C10—H10 | 1.095(2) |
| F8—C13 | 1.352(1) | C11—H11A | 1.095(2) |
| C1—H1A | 1.092(1) | C11—H11B | 1.098(1) |
| C1—H1B | 1.108(2) | C13—H13 | 1.092(2) |
| C3—H3 | 1.085(1) | | |

| **Bond angle (°)** | | | |
|---|---|---|---|
| **Bond** | **Bond angle (°)** | **Bond** | **Bond angle (°)** |
| O1—C1—H1A | 111.4(1) | O1—C8—H8A | 107.0(1) |
| O1—C1—H1B | 109.2(2) | O1—C8—H8B | 111.3(1) |
| C2—C1—H1A | 109.8(1) | C9—C8—H8A | 108.4(1) |
| C2—C1—H1B | 109.8(1) | C9—C8—H8B | 108.8(1) |
| H1A—C1—H1B | 108.6(1) | H8A—C8—H8B | 109.6(1) |
| C2—C3—H3 | 120.1(1) | F3—C10—H10 | 109.0(1) |
| C4—C3—H3 | 121.2(1) | F4—C10—H10 | 109.9(1) |
| C3—C4—H4 | 120.9(1) | C9—C10—H10 | 111.9(1) |
| C5—C4—H4 | 119.8(1) | O2—C11—H11A | 109.6(1) |
| C4—C5—H5 | 121.4(1) | O2—C11—H11B | 110.8(1) |
| C6—C5—H5 | 120.0(1) | C12—C11—H11A | 109.9(1) |
| O2—C7—H7A | 110.3(1) | C12—C11—H11B | 108.6(1) |
| O2—C7—H7B | 109.6(1) | H11A—C11—H11B | 109.8(1) |
| C6—C7—H7A | 110.1(1) | F7—C13—H13 | 109.6(1) |
| C6—C7—H7B | 109.6(1) | F8—C13—H13 | 110.2(1) |
| H7A—C7—H7B | 108.4(1) | C12—C13—H13 | 112.5(1) |

**Table S6.** Crystallographic data and refinement parameters for **4FCl-ZnI$_2$(II)** complex.

| | **X-ray diffraction CCDC ID: 2351847** | **Neutron diffraction CCDC ID: 2352174** |
|---|---|---|
| Crystal data | | |
| Chemical formula | $C_{13}H_{11}Cl_2F_8I_2NO_2Zn$ | $C_{13}H_{11}Cl_2F_8I_2NO_2Zn$ |
| $M_r$ | 755.30 | 755.30 |
| Crystal system, space group | Monoclinic, $C2/c$ | Monoclinic, $C2/c$ |
| Temperature (K) | 100 | 40 |
| $a$, $b$, $c$ (Å) | 22.8928 (3), 13.9616 (2), 17.3746 (2) | 22.9805 (1), 13.9724 (1), 17.4000 (1) |
| α, β, γ (°) | 129.863 (1) | 90, 129.9252(3), 90 |
| $V$ (Å$^3$) | 4262.57 (11) | 4284.58 (5) |
| $Z$ | 8 | 8 |
| Radiation type | Cu $K$a | Neutrons, l = 1.000 Å |
| m (mm$^{-1}$) | 27.42 | 0.21 |
| Crystal size (mm) | 0.21 × 0.17 × 0.09 | 2.50 × 2.50 × 1.0 |
| Data collection | | |
| Diffractometer | XtaLAB Synergy R, DW system, HyPix-Arc 150 | Single-crystal diffractometer SENJU at the BL18 in the MLF of the J-PARC |
| Absorption correction | Multi-scan[a] | For a sphere[b] |
| $T_{min}$, $T_{max}$ | 0.574, 1.000 | 0.704, 0.748 |
| No. of measured, independent and observed [$I$ > 2s($I$)] reflections | 16390, 4107, 3916 | 73070, 13313, 9208 |
| $R_{int}$ | 0.039 | 0.214 |
| (sin q/l)$_{max}$ (Å$^{-1}$) | 0.622 | 0.995 |
| Refinement | | |
| $R[F^2 > 2s(F^2)]$, $wR(F^2)$, $S$ | 0.028, 0.071, 1.06 | 0.072, 0.185, 1.03 |
| No. of reflections | 4107 | 13313 |
| No. of parameters | 263 | 362 |
| H-atom treatment | H-atom parameters constrained | All H-atom parameters refined |
| Δρ$_{max}$, Δρ$_{min}$ | 1.16, -0.76(e Å$^{-3}$) | 1.91, -2.76(fm Å$^{-3}$) |

(a) Bruker SADABS, 1996. (b) Tibballs, J. E., Acta Cryst. A38, 161-163, 1982.

**Table S7.** Weak interactions of tetrel bond and improper HB in **4FCl-ZnI$_2$(II)** complex [ Å and °].

| **Item no** | **a) Tetrel bond (O−C···F−C) in 4FCl-ZnI$_2$(II)** | | | | |
|---|---|---|---|---|---|
| | **H/C—C···F—C** | **H/C—C** | **C···F** | **H/C···F or C···C** | **∠CFC** |
| 1 | C2−C1···F2−C9 | C2−C1= 1.502(1)<br>F2−C9 = 1.352(3) | C1···F2=2.956(1) | C2···F2= 4.350(1)<br>C1···C9= 3.098(1) | ∠C2C1F2= 153.2(1)<br>∠C9F2C1= 83.1(1) |
| | **b) Improper hydrogen bonds (C−H···*A*)** | | | | |
| | ***D*—H···*A*** | ***D*—H** | **H···*A*** | ***D*···*A*** | **∠*D*H*A*** |
| 2 | C1—H1A···F2 | 1.095(4) | 2.351(3) | 2.956(1) | 113.0(2) |

Note: neutron structure of **4FCl-ZnI$_2$** was colloected at 40K, so the libration correction was not necessary.

**Table S8.** Selected bond lengths (Å ) and bond angles (°) for monomeric **4FCl-ZnI₂(II)** complex (at 40K).

| Bond length (Å) | | | |
|---|---|---|---|
| **Bond** | **Bond length (Å)** | **Bond** | **Bond length (Å)** |
| F1—C9 | 1.348(2) | C1—H1B | 1.108(2) |
| F2—C9 | 1.352(2) | C3—H3 | 1.082(2) |
| F3—C10 | 1.345(2) | C4—H4 | 1.094(2) |
| F4—C10 | 1.333(2) | C5—H5 | 1.091(2) |
| F5—C12 | 1.342(2) | C7—H7A | 1.098(3) |
| F6—C12 | 1.350(2) | C7—H7B | 1.101(3) |
| F7—C13 | 1.337(2) | C8—H8A | 1.098(3) |
| F8—C13 | 1.329(2) | C8—H8B | 1.100(3) |
| C1—H1A | 1.095(4) | C11—H11A | 1.093(3) |
| | | C11—H11B | 1.101(3) |

| Bond angle (°) | | | |
|---|---|---|---|
| **Bond** | **Bond angle (°)** | **Bond** | **Bond angle (°)** |
| H1A—C1—H1B | 108.5(2) | H7B—C7—O2 | 109.4(2) |
| H1A—C1—O1 | 111.5(2) | H7A—C7—C6 | 110.3(2) |
| H1B—C1—O1 | 109.4(2) | H7B—C7—C6 | 108.8(2) |
| H1A—C1—C2 | 109.9(2) | H8A—C8—H8B | 108.8(3) |
| H1B—C1—C2 | 110.7(2) | H8A—C8—O1 | 107.2(2) |
| H3—C3—C4 | 121.6(2) | H8B—C8—O1 | 111.7(2) |
| H3—C3—C2 | 119.7(2) | H8A—C8—C9 | 107.8(2) |
| H4—C4—C5 | 120.2(2) | H8B—C8—C9 | 107.3(2) |
| H4—C4—C3 | 120.5(2) | H11A—C11—H11B | 110.1(3) |
| H5—C5—C4 | 120.5(2) | H11A—C11—O2 | 110.6(2) |
| H5—C5—C6 | 120.7(2) | H11B—C11—O2 | 110.2(2) |
| H7A—C7—H7B | 108.8(3) | H11A—C11—C12 | 109.9(2) |
| H7A—C7—O2 | 110.5(2) | H11B—C11—C12 | 107.5(2) |

Note: neutron structure of **4FCl-ZnI₂** was colloected at 40K, so the libration correction was not necessary.

**Table S9**. Crystallographic data and refinement parameters for **2FCl-ZnI$_2$(III)** complex.

| | **X-ray diffraction CCDC ID: 2351848** | **Neutron diffraction CCDC ID: 2352218** |
|---|---|---|
| Crystal data | | |
| Chemical formula | $C_{11}H_{11}Cl_2F_4I_2NO_2Zn$ | $C_{11}H_{11}Cl_2F_4I_2NO_2Zn$ |
| $M_r$ | 655.28 | 655.28 |
| Crystal system, space group | Monoclinic, $C2/c$ | Monoclinic, $C2/c$ |
| Temperature (K) | 100 | 100 |
| $a$, $b$, $c$ (Å) | 8.2500 (1), 17.5030 (3), 12.8686 (2) | 8.2682 (3), 17.5189 (6), 12.9122 (5) |
| α, β, γ (°) | 90, 91.650 (2), 90 | 90, 91.699 (3), 90 |
| $V$ (Å$^3$) | 1857.45 (5) | 1869.51 (12) |
| $Z$ | 4 | 4 |
| Radiation type | Cu $K$a | Neutrons, l = 0.700 Å |
| m (mm$^{-1}$) | 31.00 | 0.0674 + 0.07163λ |
| Crystal size (mm) | 0.29 × 0.18 × 0.09 | 2.25 × 1.55 × 1.50 |
| | | |
| Data collection | | |
| Diffractometer | XtaLAB Synergy R, DW system, HyPix-Arc 150 | TOPAZ |
| Absorption correction | Multi-scan[a] | Gaussian[b] |
| $T_{min}$, $T_{max}$ | 0.574, 1.000 | 0.807, 0.927 |
| No. of measured, independent and observed [$I$ > 2s($I$)] reflections | 4868, 1769, 1701 | 24644, 4535, 3820 |
| $R_{int}$ | 0.029 | 0.095 |
| (sin q/l)$_{max}$ (Å$^{-1}$) | 0.627 | 1.399 |
| | | |
| Refinement | | |
| $R[F^2 > 2s(F^2)]$, $wR(F^2)$, $S$ | 0.022, 0.053, 1.06 | 0.034, 0.079, 1.19 |
| No. of reflections | 3164 | 4535 |
| No. of parameters | 245 | 157 |
| H-atom treatment | H-atom parameters constrained | All H-atom parameters refined |
| $\Delta\rho_{max}$, $\Delta\rho_{min}$ | 0.30, -0.28(e Å$^{-3}$) | 0.71, -0.68 (fm Å$^{-3}$) |

(a) Bruker SADABS, 1996. (b) P. Coppens, L. Leiserowitz, D. Rabinovich, Acta Cryst. 18, 1035-1038 (1965).

**Table S10.** Weak interactions of tetrel bond and improper HB in **2FCl-ZnI$_2$(III)** complex [Å and °].(without libration correction).

| Item no | a) Tetrel bond (O–C···F–C) in 2FCl-ZnI$_2$(III) | | | | |
|---|---|---|---|---|---|
| | **H/C—C···F—C** | **H/C—C** | **C···F** | **H/C···F or C···C** | **∠CFC** |
| 1 | C2–C1···F1–C6 | C2–C1= 1.506(1)<br>F1–C6 = 1.347(1) | C1···F1=3.127(1) | C2···F1= 4.432(1)<br>C1···C6= 3.235(1) | ∠C2C1F1= 143.8(0)<br>∠C6F1C1= 86.3(0) |
| | **b) Improper hydrogen bonds (C–H···*A*)** | | | | |
| | ***D*—H···*A*** | ***D*—H** | **H···*A*** | ***D*···*A*** | **∠*D*H*A*** |
| 2 | C1—H1B···F1 | 1.093(1) | 2.534(2) | 2.235(1) | 112.9(1) |

**Table S11.** Weak interactions of tetrel bond and improper HB in libration-corrected **2FCl-ZnI$_2$(III)** complex [Å and °].

| Item no | a) Tetrel bond (O–C···F–C) in 2FCl-ZnI$_2$(II) | | | | |
|---|---|---|---|---|---|
| | **H/C—C···F—C** | **H/C—C** | **C···F** | **H/C···F or C···C** | **∠CFC** |
| 1 | C2–C1···F1–C6 | C2–C1= 1.508(1)<br>F1–C6 = 1.348(1) | C1···F1=3.129(1) | C2···F1= 4.435(1)<br>C1···C6= 3.237(1) | ∠C2C1F1= 143.8(0)<br>∠C6F1C1= 82.3(0) |
| | **b) Improper hydrogen bonds (C–H···*A*)** | | | | |
| | ***D*—H···*A*** | ***D*—H** | **H···*A*** | ***D*···*A*** | **∠*D*H*A*** |
| 2 | C1—H1B···F1 | 1.093(1) | 2.536(2) | 3.129(1) | 112.9(1) |

**Table S12.** Selected bond lengths (Å ) and bond angles (°) for monomeric **2FCl-ZnI$_2$(III)** complex (without libration correction).

| **Bond length (Å)** | | | |
|---|---|---|---|
| **Bond** | **Bond length (Å)** | **Bond** | **Bond length (Å)** |
| F1—C6 | 1.347(1) | C5—H5B | 1.091(1) |
| F2—C6 | 1.332(1) | C5—H5A | 1.099(1) |
| C3—H3 | 1.086(1) | C1—H1B | 1.093(1) |
| C4—H4 | 1.087(2) | C1—H1A | 1.102(1) |
| **Bond angle (°)** | | | |
| **Bond** | **Bond angle (°)** | **Bond** | **Bond angle (°)** |
| H3—C3—C2 | 120.3(1) | H5B—C5—C6 | 108.1(1) |
| H3—C3—C4 | 121.1(1) | H5A—C5—C6 | 109.0(1) |
| H4—C4—C3 | 120.2(0) | H1B—C1—H1A | 109.1(1) |
| H4—C4—C3$^{i}$ | 120.2(0) | H1B—C1—O1 | 110.8(1) |
| H5B—C5—H5A | 109.0(1) | H1A—C1—O1 | 109.2(1) |
| H5B—C5—O1 | 107.7(1) | H1B—C1—C2 | 109.8(1) |
| H5A—C5—O1 | 112.5(1) | H1A—C1—C2 | 109.5(1) |

Symmetry code: (i) -*x*+1, *y*, -*z*+1/2.

**Table S13.** Selected bond lengths (Å ) and bond angles (°) for libration corrected monomeric **2FCl-ZnI$_2$(III)** complex.

| **Bond length (Å)** | | | |
|---|---|---|---|
| **Bond** | **Bond length (Å)** | **Bond** | **Bond length (Å)** |
| F1—C6 | 1.348(1) | C3—H3 | 1.086(1) |
| F2—C6 | 1.333(1) | C4—H4 | 1.088(2) |
| C1—H1A | 1.103(1) | C5—H5A | 1.100(1) |
| C1—H1B | 1.093(1) | C5—H5B | 1.092(1) |
| **Bond angle (°)** | | | |
| **Bond** | **Bond angle (°)** | **Bond** | **Bond angle (°)** |
| O1—C1—H1A | 109.2(1) | C3—C4—H4 | 120.2(0) |
| O1—C1—H1B | 110.8(1) | C3$^{i}$—C4—H4 | 120.2(0) |
| C2—C1—H1A | 109.5(1) | O1—C5—H5A | 112.5(1) |
| C2—C1—H1B | 109.8(1) | O1—C5—H5B | 107.7(1) |
| H1A—C1—H1B | 109.1(1) | C6—C5—H5A | 109.0(1) |
| C2—C3—H3 | 120.3(1) | C6—C5—H5B | 108.1(1) |
| C4—C3—H3 | 121.1(1) | H5A—C5—H5B | 109.0(1) |

Symmetry code: (i) -*x*+1, *y*, -*z*+1/2

**Table S14.** Experimental data of the wavenumber vs. neutron C−H bond length

| Item | C−H vibration ($cm^{-1}$) | C−H Bond length (Å) | Comments |
|---|---|---|---|
| 1 | 2688 | 1.108 (2) | Complex **I** |
| 2 | 3020 | 1.092 (1) | |
| 3 | 2700 | 1.1075(0)[a] | Complex **II** |
| 4 | 2946 | 1.095 (4) | |
| 5 | 2788 | 1.103(2) | Complex **III** |
| 6 | 2985 | 1.093(1) | |

**Note:** [a] The C1−H1B bond length in **II** is based on both neutron and IR data (=2700 $cm^{-1}$) of this elongated C1−H1B bond.

## Atomic coordinates of complexes (I-III)

**Table S15.** Atomic coordinates of optimized **4FH-ZnCl$_2$(I)** monomer using MP2 theoretical calculations (H-C-H).

| Atom | X | Y | Z |
|---|---|---|---|
| Zn | 0.03072 | -0.45566 | -0.17701 |
| H | -0.24305 | -1.25396 | -1.48199 |
| Cl | 0.40628 | -1.13106 | 1.91671 |
| F | 4.66308 | 0.18813 | -1.52882 |
| F | 5.03598 | 0.45804 | 0.63153 |
| F | -6.64586 | -1.46636 | 0.07856 |
| O | -2.18814 | 0.24622 | 0.15869 |
| O | 2.22826 | 0.3028 | -0.35547 |
| F | 5.09973 | -2.48212 | -1.28937 |
| F | -4.95209 | 0.23546 | -0.9608 |
| F | 5.38092 | -2.24692 | 0.89573 |
| F | -4.64549 | 0.0923 | 1.22191 |
| F | -5.06978 | -2.56929 | -1.02488 |
| N | 0.0011 | 1.61318 | -0.10862 |
| C | 1.11383 | 2.28724 | 0.22217 |
| C | -1.14543 | 2.27026 | -0.35315 |
| C | -0.06338 | 4.37167 | 0.04983 |
| H | -0.08775 | 5.45168 | 0.11145 |
| C | 1.12007 | 3.6793 | 0.30097 |
| H | 2.03094 | 4.20494 | 0.55485 |
| C | -3.00427 | -0.86478 | -0.19048 |
| H | -2.67622 | -1.67267 | 0.46238 |
| H | -2.85767 | -1.16383 | -1.22999 |
| C | -4.47274 | -0.57488 | 0.04197 |
| C | -1.21758 | 3.65894 | -0.27518 |
| H | -2.15458 | 4.16666 | -0.46103 |
| C | 4.5488 | -0.42273 | -0.31251 |
| C | 2.30722 | 1.42763 | 0.5308 |
| H | 3.23235 | 1.9825 | 0.37641 |
| H | 2.25976 | 1.0761 | 1.56672 |
| C | -2.30475 | 1.38128 | -0.70725 |
| H | -3.25312 | 1.89698 | -0.5569 |
| H | -2.24031 | 1.05721 | -1.75303 |
| C | -5.3369 | -1.82612 | 0.09147 |
| H | -5.13342 | -2.4232 | 0.97987 |
| C | 3.10672 | -0.77144 | -0.02293 |
| H | 2.81441 | -1.60035 | -0.66428 |
| H | 2.99452 | -1.06561 | 1.02064 |
| C | 5.48803 | -1.6182 | -0.30945 |
| H | 6.52099 | -1.31507 | -0.47642 |

Note: The calculations of complex (**I**) have been done by using MP2/ 6-31G(d,p) level of theory.

**Table S16.** Atomic coordinates of optimized **4FH-ZnCl$_2$(I)** monomer using MP2 theoretical calculations (D-C-H).

| Atom | X | Y | Z |
|---|---|---|---|
| Zn | 0.03077 | -0.4558 | -0.17627 |
| H | -0.24327 | -1.25602 | -1.47998 |
| Cl | 0.40739 | -1.12824 | 1.91834 |
| F | 4.66279 | 0.18632 | -1.53046 |
| F | 5.03639 | 0.45884 | 0.62943 |
| F | -6.64602 | -1.46667 | 0.07837 |
| O | -2.1883 | 0.24597 | 0.15876 |
| O | 2.22843 | 0.30277 | -0.35606 |
| F | 5.09937 | -2.48369 | -1.28789 |
| F | -4.95224 | 0.23503 | -0.96103 |
| F | 5.3813 | -2.24583 | 0.89683 |
| F | -4.64584 | 0.09204 | 1.22175 |
| F | -5.06988 | -2.56956 | -1.02508 |
| N | 0.00108 | 1.61304 | -0.10933 |
| C | 1.11377 | 2.2873 | 0.22121 |
| C | -1.14566 | 2.26997 | -0.35338 |
| C | -0.06394 | 4.37153 | 0.04965 |
| H | -0.08852 | 5.45152 | 0.11151 |
| C | 1.11971 | 3.67935 | 0.30028 |
| H | 2.03056 | 4.20514 | 0.55396 |
| C | -3.00445 | -0.86508 | -0.19035 |
| H | -2.67652 | -1.6729 | 0.46266 |
| H | -2.85762 | -1.16428 | -1.22977 |
| C | -4.47294 | -0.57521 | 0.04191 |
| C | -1.21809 | 3.65861 | -0.27512 |
| H | -2.15525 | 4.16618 | -0.46059 |
| C | 4.54886 | -0.42305 | -0.31338 |
| C | 2.30751 | 1.42808 | 0.52961 |
| H | 3.23242 | 1.9831 | 0.37442 |
| H | 2.26064 | 1.07708 | 1.56573 |
| C | -2.305 | 1.38094 | -0.70727 |
| H | -3.25334 | 1.89668 | -0.55678 |
| H | -2.24077 | 1.05679 | -1.75304 |
| C | -5.3371 | -1.8265 | 0.0913 |
| H | -5.13368 | -2.42366 | 0.97967 |
| C | 3.10685 | -0.77132 | -0.02293 |
| H | 2.81421 | -1.60077 | -0.66343 |
| H | 2.99501 | -1.06457 | 1.02094 |
| C | 5.48803 | -1.61859 | -0.30916 |
| H | 6.52094 | -1.31572 | -0.47684 |

**Table S17.** Atomic coordinates of optimized **4FH-ZnCl$_2$(I)** monomer using MP2 theoretical calculations (H-C-D).

| Atom | X | Y | Z |
|---|---|---|---|
| Zn | 0.03077 | -0.4558 | -0.17627 |
| H | -0.24327 | -1.25602 | -1.47998 |
| Cl | 0.40739 | -1.12824 | 1.91834 |
| F | 4.66279 | 0.18632 | -1.53046 |
| F | 5.03639 | 0.45884 | 0.62943 |
| F | -6.64602 | -1.46667 | 0.07837 |
| O | -2.1883 | 0.24597 | 0.15876 |
| O | 2.22843 | 0.30277 | -0.35606 |
| F | 5.09937 | -2.48369 | -1.28789 |
| F | -4.95224 | 0.23503 | -0.96103 |
| F | 5.3813 | -2.24583 | 0.89683 |
| F | -4.64584 | 0.09204 | 1.22175 |
| F | -5.06988 | -2.56956 | -1.02508 |
| N | 0.00108 | 1.61304 | -0.10933 |
| C | 1.11377 | 2.2873 | 0.22121 |
| C | -1.14566 | 2.26997 | -0.35338 |
| C | -0.06394 | 4.37153 | 0.04965 |
| H | -0.08852 | 5.45152 | 0.11151 |
| C | 1.11971 | 3.67935 | 0.30028 |
| H | 2.03056 | 4.20514 | 0.55396 |
| C | -3.00445 | -0.86508 | -0.19035 |
| H | -2.67652 | -1.6729 | 0.46266 |
| H | -2.85762 | -1.16428 | -1.22977 |
| C | -4.47294 | -0.57521 | 0.04191 |
| C | -1.21809 | 3.65861 | -0.27512 |
| H | -2.15525 | 4.16618 | -0.46059 |
| C | 4.54886 | -0.42305 | -0.31338 |
| C | 2.30751 | 1.42808 | 0.52961 |
| H | 3.23242 | 1.9831 | 0.37442 |
| H | 2.26064 | 1.07708 | 1.56573 |
| C | -2.305 | 1.38094 | -0.70727 |
| H | -3.25334 | 1.89668 | -0.55678 |
| H | -2.24077 | 1.05679 | -1.75304 |
| C | -5.3371 | -1.8265 | 0.0913 |
| H | -5.13368 | -2.42366 | 0.97967 |
| C | 3.10685 | -0.77132 | -0.02293 |
| H | 2.81421 | -1.60077 | -0.66343 |
| H | 2.99501 | -1.06457 | 1.02094 |
| C | 5.48803 | -1.61859 | -0.30916 |
| H | 6.52094 | -1.31572 | -0.47684 |

**Table S18.** Atomic coordinates of optimized **4FCl-ZnI$_2$(II)** monomer using MP2 theoretical calculations (H-C-H).

| Atom | X | Y | Z |
|---|---|---|---|
| I | -0.1578 | -1.05297 | 2.3422 |
| I | 0.34902 | -1.51417 | -2.34814 |
| Zn | -0.0504 | -0.17096 | -0.12973 |
| Cl | 7.27278 | 0.02831 | -0.15869 |
| Cl | -6.81763 | 0.0028 | 1.09989 |
| F | 4.78422 | 1.69706 | 0.6776 |
| F | 4.43044 | 0.59391 | -1.3024 |
| F | -5.28389 | -2.01209 | -0.55094 |
| F | -5.18348 | 0.00613 | -1.64903 |
| F | -4.32268 | -0.56293 | 1.85198 |
| F | -4.58953 | 1.37828 | 0.65804 |
| F | 5.70038 | -0.70388 | 1.8673 |
| F | 5.36628 | -1.83343 | -0.09381 |
| O | -2.29447 | 0.18349 | -0.53215 |
| O | 2.01553 | 0.80376 | 0.13461 |
| N | -0.31605 | 1.89342 | -0.35571 |
| C | 0.62396 | 2.75631 | 0.15484 |
| C | -1.43506 | 2.3655 | -1.00056 |
| C | 4.4566 | 0.40304 | 0.11121 |
| C | -1.6588 | 3.76016 | -1.14486 |
| H | -2.56083 | 4.12066 | -1.645 |
| C | -2.37315 | 1.29189 | -1.54217 |
| H | -3.40065 | 1.67388 | -1.6148 |
| H | -2.02758 | 0.89754 | -2.5159 |
| C | -4.61572 | -0.74226 | -0.54801 |
| C | -3.11685 | -1.01282 | -0.79923 |
| H | -2.76061 | -1.77246 | -0.09039 |
| H | -2.99883 | -1.36262 | -1.83845 |
| C | 5.65147 | -0.54647 | 0.44684 |
| C | -0.6912 | 4.66821 | -0.62839 |
| H | -0.83757 | 5.7459 | -0.73245 |
| C | -5.01625 | 0.01838 | 0.76047 |
| C | 3.08818 | -0.07407 | 0.64181 |
| H | 2.86405 | -1.07148 | 0.23904 |
| H | 3.09703 | -0.09799 | 1.74349 |
| C | 1.8022 | 2.09657 | 0.86663 |
| H | 2.7005 | 2.72559 | 0.79819 |
| H | 1.56142 | 1.86343 | 1.92122 |
| C | 0.46824 | 4.1623 | 0.02456 |
| H | 1.22969 | 4.83654 | 0.42406 |

**Table S19.** Atomic coordinates of optimized **4FCl-ZnI$_2$(II)** monomer using MP2 theoretical calculations (D-C-H).

| **Atom** | **X** | **Y** | **Z** |
|---|---|---|---|
| I | -0.1578 | -1.05297 | 2.3422 |
| I | 0.34902 | -1.51417 | -2.34814 |
| Zn | -0.0504 | -0.17096 | -0.12973 |
| Cl | 7.27278 | 0.02831 | -0.15869 |
| Cl | -6.81763 | 0.0028 | 1.09989 |
| F | 4.78422 | 1.69706 | 0.6776 |
| F | 4.43044 | 0.59391 | -1.3024 |
| F | -5.28389 | -2.01209 | -0.55094 |
| F | -5.18348 | 0.00613 | -1.64903 |
| F | -4.32268 | -0.56293 | 1.85198 |
| F | -4.58953 | 1.37828 | 0.65804 |
| F | 5.70038 | -0.70388 | 1.8673 |
| F | 5.36628 | -1.83343 | -0.09381 |
| O | -2.29447 | 0.18349 | -0.53215 |
| O | 2.01553 | 0.80376 | 0.13461 |
| N | -0.31605 | 1.89342 | -0.35571 |
| C | 0.62396 | 2.75631 | 0.15484 |
| C | -1.43506 | 2.3655 | -1.00056 |
| C | 4.4566 | 0.40304 | 0.11121 |
| C | -1.6588 | 3.76016 | -1.14486 |
| H | -2.56083 | 4.12066 | -1.645 |
| C | -2.37315 | 1.29189 | -1.54217 |
| H | -3.40065 | 1.67388 | -1.6148 |
| H | -2.02758 | 0.89754 | -2.5159 |
| C | -4.61572 | -0.74226 | -0.54801 |
| C | -3.11685 | -1.01282 | -0.79923 |
| H | -2.76061 | -1.77246 | -0.09039 |
| H | -2.99883 | -1.36262 | -1.83845 |
| C | 5.65147 | -0.54647 | 0.44684 |
| C | -0.6912 | 4.66821 | -0.62839 |
| H | -0.83757 | 5.7459 | -0.73245 |
| C | -5.01625 | 0.01838 | 0.76047 |
| C | 3.08818 | -0.07407 | 0.64181 |
| H | 2.86405 | -1.07148 | 0.23904 |
| H | 3.09703 | -0.09799 | 1.74349 |
| C | 1.8022 | 2.09657 | 0.86663 |
| H | 2.7005 | 2.72559 | 0.79819 |
| H | 1.56142 | 1.86343 | 1.92122 |
| C | 0.46824 | 4.1623 | 0.02456 |
| H | 1.22969 | 4.83654 | 0.42406 |

**Table S20.** Atomic coordinates of optimized **4FCl-ZnI$_2$(II)** monomer using MP2 theoretical calculations (H-C-D).

| Atom | X | Y | Z |
|---|---|---|---|
| I | -0.1578 | -1.05297 | 2.3422 |
| I | 0.34902 | -1.51417 | -2.34814 |
| Zn | -0.0504 | -0.17096 | -0.12973 |
| Cl | 7.27278 | 0.02831 | -0.15869 |
| Cl | -6.81763 | 0.0028 | 1.09989 |
| F | 4.78422 | 1.69706 | 0.6776 |
| F | 4.43044 | 0.59391 | -1.3024 |
| F | -5.28389 | -2.01209 | -0.55094 |
| F | -5.18348 | 0.00613 | -1.64903 |
| F | -4.32268 | -0.56293 | 1.85198 |
| F | -4.58953 | 1.37828 | 0.65804 |
| F | 5.70038 | -0.70388 | 1.8673 |
| F | 5.36628 | -1.83343 | -0.09381 |
| O | -2.29447 | 0.18349 | -0.53215 |
| O | 2.01553 | 0.80376 | 0.13461 |
| N | -0.31605 | 1.89342 | -0.35571 |
| C | 0.62396 | 2.75631 | 0.15484 |
| C | -1.43506 | 2.3655 | -1.00056 |
| C | 4.4566 | 0.40304 | 0.11121 |
| C | -1.6588 | 3.76016 | -1.14486 |
| H | -2.56083 | 4.12066 | -1.645 |
| C | -2.37315 | 1.29189 | -1.54217 |
| H | -3.40065 | 1.67388 | -1.6148 |
| H | -2.02758 | 0.89754 | -2.5159 |
| C | -4.61572 | -0.74226 | -0.54801 |
| C | -3.11685 | -1.01282 | -0.79923 |
| H | -2.76061 | -1.77246 | -0.09039 |
| H | -2.99883 | -1.36262 | -1.83845 |
| C | 5.65147 | -0.54647 | 0.44684 |
| C | -0.6912 | 4.66821 | -0.62839 |
| H | -0.83757 | 5.7459 | -0.73245 |
| C | -5.01625 | 0.01838 | 0.76047 |
| C | 3.08818 | -0.07407 | 0.64181 |
| H | 2.86405 | -1.07148 | 0.23904 |
| H | 3.09703 | -0.09799 | 1.74349 |
| C | 1.8022 | 2.09657 | 0.86663 |
| H | 2.7005 | 2.72559 | 0.79819 |
| H | 1.56142 | 1.86343 | 1.92122 |
| C | 0.46824 | 4.1623 | 0.02456 |
| H | 1.22969 | 4.83654 | 0.42406 |

**Table S21.** Atomic coordinates of optimized **2FCl-ZnI$_2$**(**III**) monomer using MP2 theoretical calculations (H-C-H).

| Atom | X | Y | Z |
|---|---|---|---|
| I | 0.00298 | 1.23535 | -2.36961 |
| Zn | 4E-6 | 0.09862 | 4.3E-5 |
| Cl | -5.7489 | 1.54646 | 0.05534 |
| F | -5.11299 | -1.02267 | 0.42827 |
| F | -4.47458 | -0.14182 | -1.57499 |
| O | -2.1961 | -0.56906 | 0.11878 |
| N | 1.86E-4 | -1.99533 | -4.71E-4 |
| C | -1.13495 | -4.09214 | 0.4729 |
| H | -2.0217 | -4.61706 | 0.83634 |
| C | -1.10051 | -2.67222 | 0.46882 |
| C | -4.55372 | 0.15692 | -0.1896 |
| C | 4.36E-4 | -4.8087 | -9.75E-4 |
| H | 5.31E-4 | -5.90126 | -0.00117 |
| C | -3.18043 | 0.4903 | 0.42011 |
| H | -3.28574 | 0.64013 | 1.50711 |
| H | -2.78668 | 1.3989 | -0.05534 |
| C | -2.23731 | -1.79375 | 0.98483 |
| H | -2.0682 | -1.48413 | 2.03373 |
| H | -3.20514 | -2.30329 | 0.87698 |
| Cl | 5.74918 | 1.54686 | -0.0548 |
| F | 5.11333 | -1.0221 | -0.42898 |
| F | 4.47507 | -0.1423 | 1.57478 |
| O | 2.19647 | -0.56878 | -0.11902 |
| C | 1.13569 | -4.09176 | -0.4746 |
| H | 2.02253 | -4.61639 | -0.83825 |
| C | 1.10101 | -2.67185 | -0.47002 |
| C | 4.55407 | 0.15715 | 0.18953 |
| C | 3.18071 | 0.49079 | -0.41988 |
| H | 3.28592 | 0.6412 | -1.50682 |
| H | 2.78697 | 1.39912 | 0.05607 |
| C | 2.23764 | -1.793 | -0.98576 |
| H | 2.06833 | -1.48282 | -2.03446 |
| H | 3.20553 | -2.3025 | -0.87833 |
| I | -0.00364 | 1.23414 | 2.37027 |

**Table S22.** Atomic coordinates of optimized **2FCl-ZnI$_2$(III)** monomer using MP2 theoretical calculations (D-C-H).

| Atom | X | Y | Z |
|---|---|---|---|
| I | 0.00298 | 1.23535 | -2.36961 |
| Zn | 4E-6 | 0.09862 | 4.3E-5 |
| Cl | -5.7489 | 1.54646 | 0.05534 |
| F | -5.11299 | -1.02267 | 0.42827 |
| F | -4.47458 | -0.14182 | -1.57499 |
| O | -2.1961 | -0.56906 | 0.11878 |
| N | 1.86E-4 | -1.99533 | -4.71E-4 |
| C | -1.13495 | -4.09214 | 0.4729 |
| H | -2.0217 | -4.61706 | 0.83634 |
| C | -1.10051 | -2.67222 | 0.46882 |
| C | -4.55372 | 0.15692 | -0.1896 |
| C | 4.36E-4 | -4.8087 | -9.75E-4 |
| H | 5.31E-4 | -5.90126 | -0.00117 |
| C | -3.18043 | 0.4903 | 0.42011 |
| H | -3.28574 | 0.64013 | 1.50711 |
| H | -2.78668 | 1.3989 | -0.05534 |
| C | -2.23731 | -1.79375 | 0.98483 |
| H | -2.0682 | -1.48413 | 2.03373 |
| H | -3.20514 | -2.30329 | 0.87698 |
| Cl | 5.74918 | 1.54686 | -0.0548 |
| F | 5.11333 | -1.0221 | -0.42898 |
| F | 4.47507 | -0.1423 | 1.57478 |
| O | 2.19647 | -0.56878 | -0.11902 |
| C | 1.13569 | -4.09176 | -0.4746 |
| H | 2.02253 | -4.61639 | -0.83825 |
| C | 1.10101 | -2.67185 | -0.47002 |
| C | 4.55407 | 0.15715 | 0.18953 |
| C | 3.18071 | 0.49079 | -0.41988 |
| H | 3.28592 | 0.6412 | -1.50682 |
| H | 2.78697 | 1.39912 | 0.05607 |
| C | 2.23764 | -1.793 | -0.98576 |
| H | 2.06833 | -1.48282 | -2.03446 |
| H | 3.20553 | -2.3025 | -0.87833 |
| I | -0.00364 | 1.23414 | 2.37027 |

**Table S23.** Atomic coordinates of optimized **2FCl-ZnI$_2$**(**III**) monomer using MP2 theoretical calculations (H-C-D).

| Atom | X | Y | Z |
|---|---|---|---|
| I | 0.00298 | 1.23535 | -2.36961 |
| Zn | 4E-6 | 0.09862 | 4.3E-5 |
| Cl | -5.7489 | 1.54646 | 0.05534 |
| F | -5.11299 | -1.02267 | 0.42827 |
| F | -4.47458 | -0.14182 | -1.57499 |
| O | -2.1961 | -0.56906 | 0.11878 |
| N | 1.86E-4 | -1.99533 | -4.71E-4 |
| C | -1.13495 | -4.09214 | 0.4729 |
| H | -2.0217 | -4.61706 | 0.83634 |
| C | -1.10051 | -2.67222 | 0.46882 |
| C | -4.55372 | 0.15692 | -0.1896 |
| C | 4.36E-4 | -4.8087 | -9.75E-4 |
| H | 5.31E-4 | -5.90126 | -0.00117 |
| C | -3.18043 | 0.4903 | 0.42011 |
| H | -3.28574 | 0.64013 | 1.50711 |
| H | -2.78668 | 1.3989 | -0.05534 |
| C | -2.23731 | -1.79375 | 0.98483 |
| H | -2.0682 | -1.48413 | 2.03373 |
| H | -3.20514 | -2.30329 | 0.87698 |
| Cl | 5.74918 | 1.54686 | -0.0548 |
| F | 5.11333 | -1.0221 | -0.42898 |
| F | 4.47507 | -0.1423 | 1.57478 |
| O | 2.19647 | -0.56878 | -0.11902 |
| C | 1.13569 | -4.09176 | -0.4746 |
| H | 2.02253 | -4.61639 | -0.83825 |
| C | 1.10101 | -2.67185 | -0.47002 |
| C | 4.55407 | 0.15715 | 0.18953 |
| C | 3.18071 | 0.49079 | -0.41988 |
| H | 3.28592 | 0.6412 | -1.50682 |
| H | 2.78697 | 1.39912 | 0.05607 |
| C | 2.23764 | -1.793 | -0.98576 |
| H | 2.06833 | -1.48282 | -2.03446 |
| H | 3.20553 | -2.3025 | -0.87833 |
| I | -0.00364 | 1.23414 | 2.37027 |

## Other related experimental data

### (A) Synthesis of deuterated 4FH-$ZnCl_2$ and related compounds

[Note: Both non-deuterated and deuterated syntheses are the same, and the non-deuterated syntheses are shown in the main content.]

**Synthesis of deuterated 2,6-py-$(CD_2OD)_2$**

To a stirring solution of the 2,6-pincer-dimethyl ester (0.877 g, 4.5 mmol) in 60 mL anhydrous ethanol under an atmosphere of $N_2$, $NaBD_4$ (1.0 g, 24.05 mmol) and $CaCl_2$ (3.0 g, 27 mmol) was added slowly in portions at at 0°C. The evolution of $D_2$ was allowed to cease before each further addition. The reaction mixture was then stirred at 0°C. for 6-7h. When the reaction is complete the solvent was removed under vacuum leaving a white powder. Then add 100 mL distilled water and add conc. HCl to neutralize the reaction in an ice bath environment. To the resultant mixture, add ethyl acetate and kept in sonication for 40 min. Then collect the clean organic layer using separatory funnel, repeat this procedure for three times and concentrated under reduced pressure to give the deuterated 2,6-py-$(CD_2OD)_2$ as white solid.

Data for deuterated 2,6-py-$(CD_2OD)_2$: Yield: 77.7%; m.p. 118-120 °C; $^1$H NMR (400 MHz, DNSO-$d_6$, ppm): δ= 7.79 (1H, t, $^3J_{H,H}$= 7.8 Hz, H-4), 7.32 (2H, d, $^3J_{H,H}$= 7.8 Hz, $H_{3/5}$), 3.93 (2H, br., $CD_2O\underline{H}$); FT-IR, (ATR, $cm^{-1}$) υ= 3300 (O-H), 3065 (C-H; −py-H), 2230, 2192 (py-$C\underline{D}_2$), 1593 (C=C; −py), 1577 (C=N; −py).

**Synthesis of deuterated 2,6-py-$(CD_2Br)_2$**

Deuterated 2,6-pyridinedimethanol (6.91 mmol, 0.99 g) was dissolved in 5.3 mL dimethylformamide. Then phosphorus tribromide (16.25 mmol, 1.54 mL) was added drop by drop and stirred in an ice bath for 4 hours and left at room temperature overnight. After the reaction was completed, the reaction was quenched with large amount of water. The resulting solution was extracted three times with diethyl ether and the extractive was concentrated under vacuum to give the deuterated 2,6-py-$(CD_2Br)_2$ as white solid.

Data for deuterated 2,6-py-$(CD_2Br)_2$: Yield: 40%; m.p. 108-110 °C; $^1$H NMR (400 MHz, $CDCl_3$, ppm): δ= 7.70 (1H, t, $^3J_{H,H}$= 7.7 Hz, H-4), 7.36 (2H, d, $^3J_{H,H}$= 7.7 Hz, $H_{3/5}$).

**Synthesis of deuterated 2,6-($HCF_2CF_2CH_2OCD_2$)$_2$-py**

A magnetic stirrer and vacuum system were equipped in a 250 mL double-necked round-bottom flask. The air and moisture were pumped out of the flask using the vacuum system. $HCF_2CF_2CH_2OH$ (2.5 mmol, 0.32 g) and $CH_3ONa/CH_3OH$ (1.9 mmol, 0.35 g) were mixed in a two-necked flask and continuously stirred at 60 $^0$C under nitrogen for 4 hours. The methanol was removed using a vacuum system to move the reaction to the product side. The obtained sodium fluorinated alkoxide was dissolved in dry THF, and then deuterated 2,6-py-($CD_2Br$)$_2$ (0.7 mmol, 0.20 g) was added, and the mixture was continuously stirred for 4 hours under a nitrogen atmosphere at room temperature. After confirming that the ligand was formed by using GC/MS analysis, the reaction was quenched by adding water. The resulting solution was extracted with dichloromethane and water. The organic phase was then dried over $Na_2SO_4$ and concentrated under reduced pressure to give a 2,6-($HCF_2CF_2CH_2OCD_2$)$_2$-py as a colorless liquid.

Data for deuterated 2,6-($HCF_2CF_2CH_2OCD_2$)$_2$-py: Yield: 83.8%; $^1$H NMR (400 MHz, $CDCl_3$, ppm): δ= 7.75 (1H, t, $^3J_{H,H}$= 7.7 Hz, H-4), 7.33 (2H, d, $^3J_{H,H}$= 7.7 Hz, $H_{3/5}$), 3.93 (4H, t, $^3J_{H,F}$= 12.5 Hz, O$C\underline{H}_2$), 5.96 (2H, tt, $^3J_{F,H}$= 53.2 Hz, $C_2F_4\underline{H}$), 5.96 (2H, tt, $^4J_{H,F}$= 5 Hz, $C_2F_4\underline{H}$).

**Synthesis of deuterated 4FH-ZnCl$_2$(I), [2,6-($HCF_2CF_2CH_2OCD_2$)$_2$-py-ZnCl$_2$], complex**

A 25 mL single-necked round-bottomed flask was equipped with a magnetic stirrer and then filled sequentially with 2,6-($HCF_2CF_2CH_2OCD_2$)$_2$-py (0.27 mmol, 0.10 g), $ZnCl_2$ (0.27 mmol, 0.37 g) and 5 mL dichloromethane. Under $N_2$ atmosphere, this mixture was then stirred overnight with the round-bottomed flask immersed in an oil bath at room temperature. At the end of the reaction, the solvent was removed by vacuum system to leave an white powder as a crude product.The solid product was collected dried in an oven at 60 °C to give deuterated **4FH-ZnCl$_2$(I)** complex.

Data for deuterated **4FH-ZnCl$_2$(I)** complex: Yield: 83.8% ; m.p. 109-112 °C; $^1$H NMR (400 MHz, $CDCl_3$, ppm): δ= 8.04 (1H, t, $^3J_{H,H}$= 8 Hz, H-4), 7.40 (2H, d, $^3J_{H,H}$= 8 Hz, $H_{3/5}$), 3.93 (4H, t, $^3J_{H,F}$= 12.5 Hz, O$C\underline{H}_2$), 6.16 (2H, tt, $^3J_{F,H}$= 52.6 Hz, $C_2F_4\underline{H}$), 6.16 (2H, tt, $^4J_{H,F}$= 4.9 Hz, $C_2F_4\underline{H}$); FT-IR, (ATR, cm$^{-1}$) υ= 3098, 3048 (C-H; −py-H), 3009 (C-H; −$CF_2H$), 2948, 2923, 2851 (C-H; −$\underline{C}H_2$−$CF_2$ & −py−$\underline{C}H_2$), 2220, 1970 ($CD_2$; local mode), 1612 (C=C; −py), 1583 (C=N; −py), 1113, 1090, 1074, 1028 (C−F).

## VI. NMR ($^1$H, $^{13}$C and $^{19}$F) spectra

### 1. **4FH-ZnCl$_2$(I)**

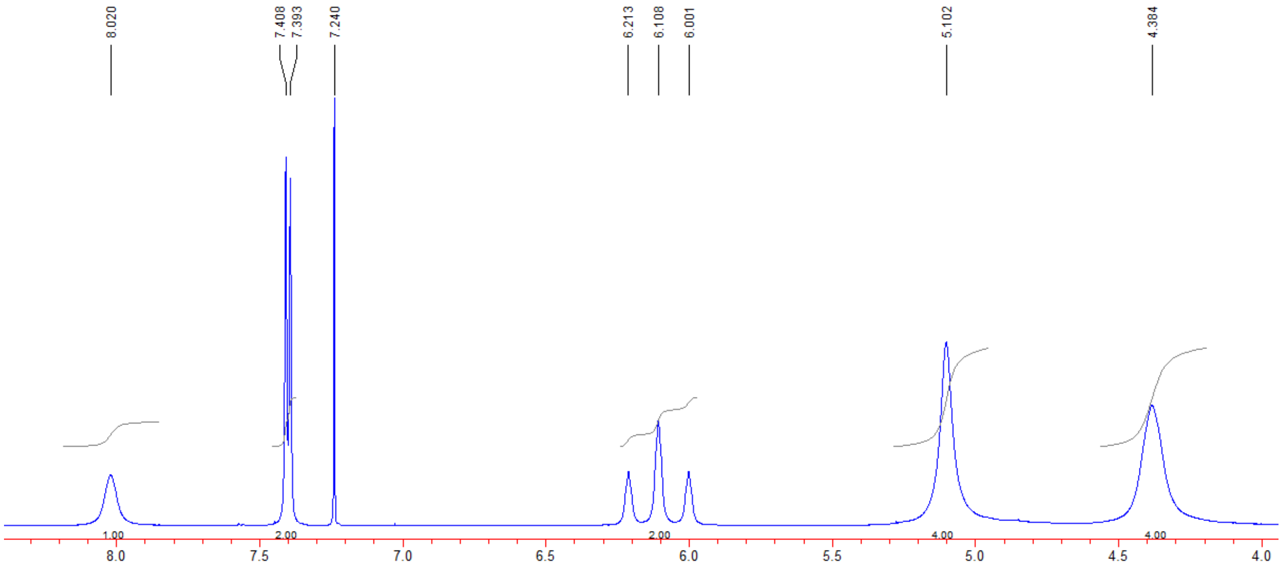


**Figure S6.** $^1$H NMR spectrum of **4FH-ZnlCl$_2$(I)** complex.

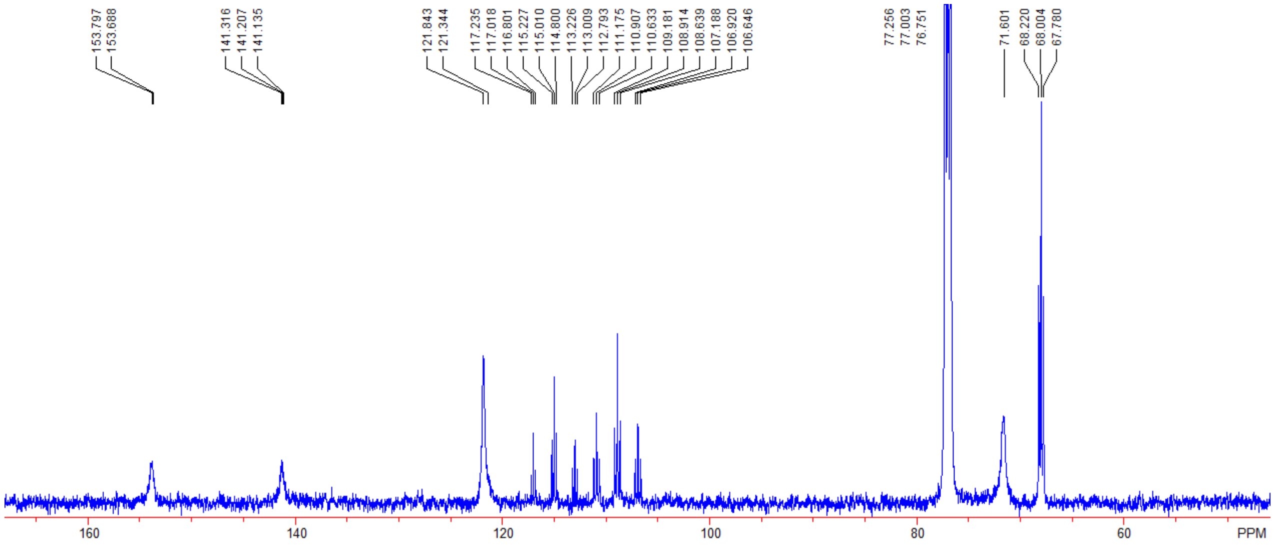


**Figure S7.** $^{13}$C NMR spectrum of **4FH-ZnlCl$_2$(I)** complex.

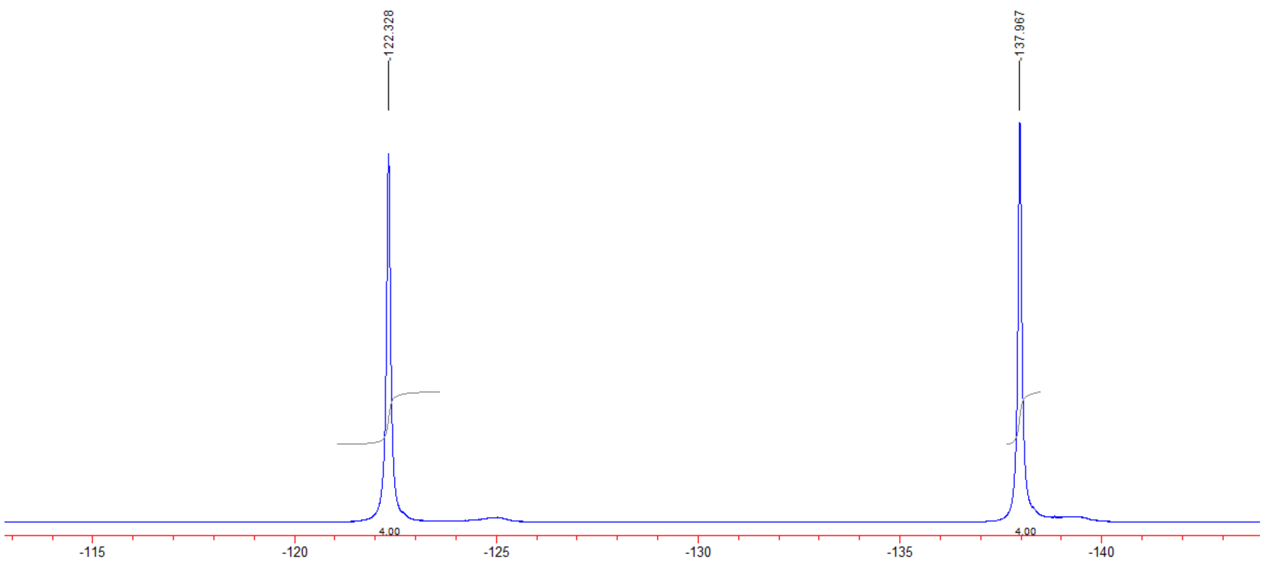


**Figure S8.** $^{19}$F NMR spectrum of **4FH-ZnlCl$_2$(I)** complex.

## 2. 4FCl-ZnI$_2$(II)

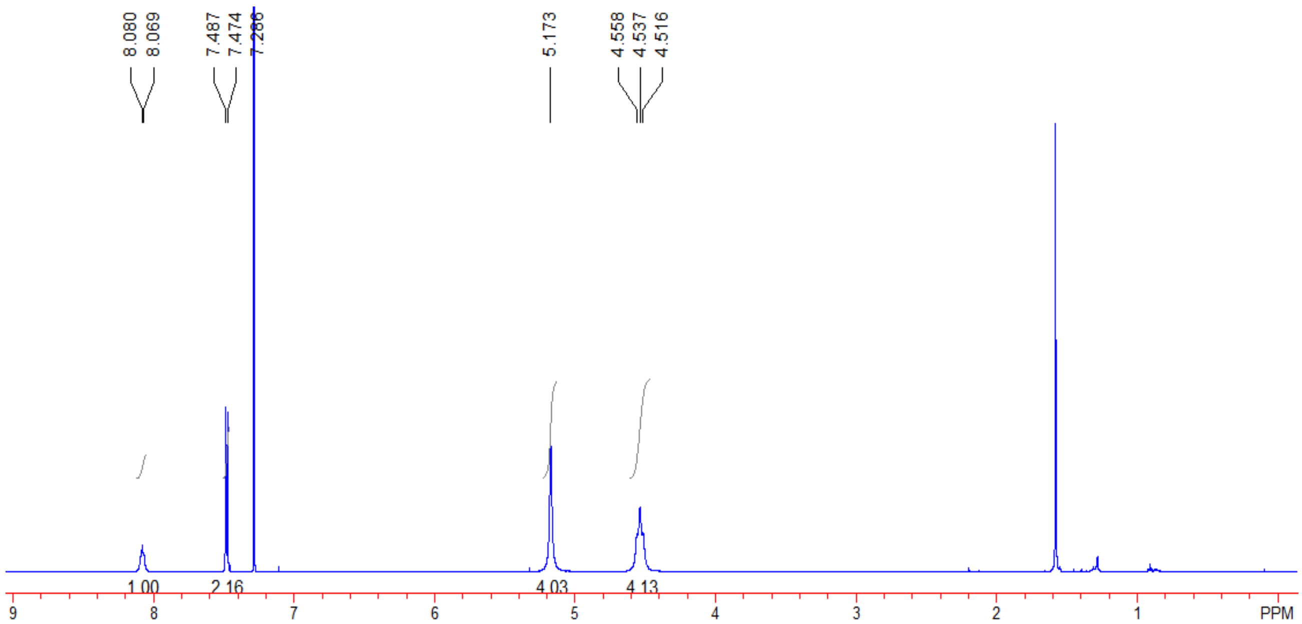


**Figure S9.** $^{1}$H NMR spectrum of **4FCl-Znl$_2$(II)** complex.

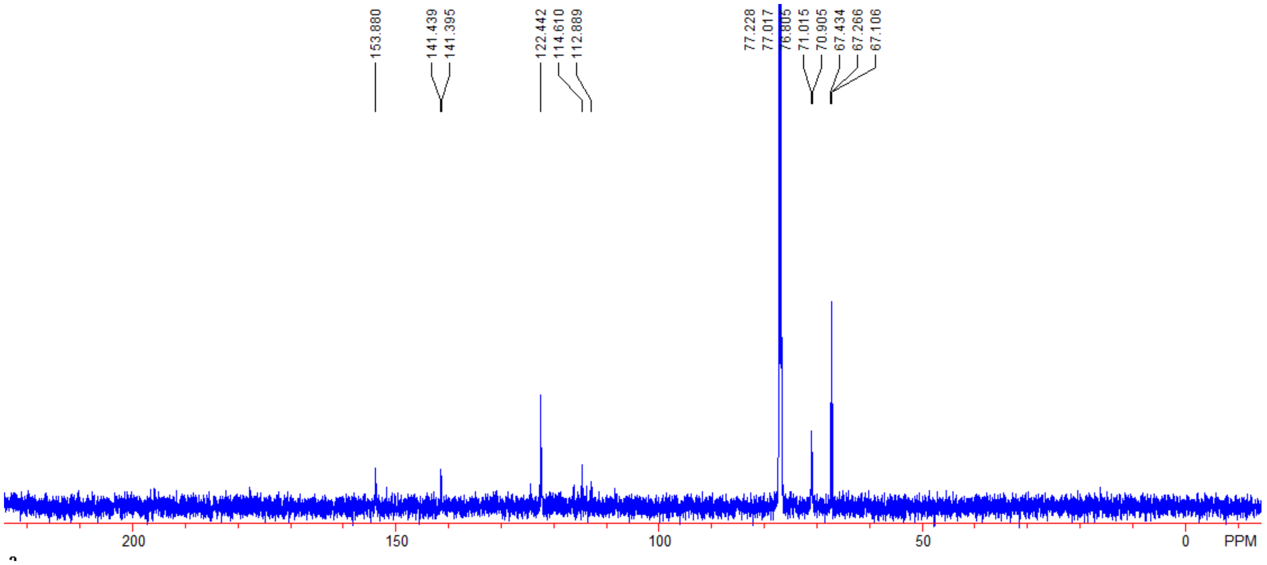


**Figure S10.** $^{13}C$ NMR spectrum of **4FCl-ZnI$_2$(II)** complex

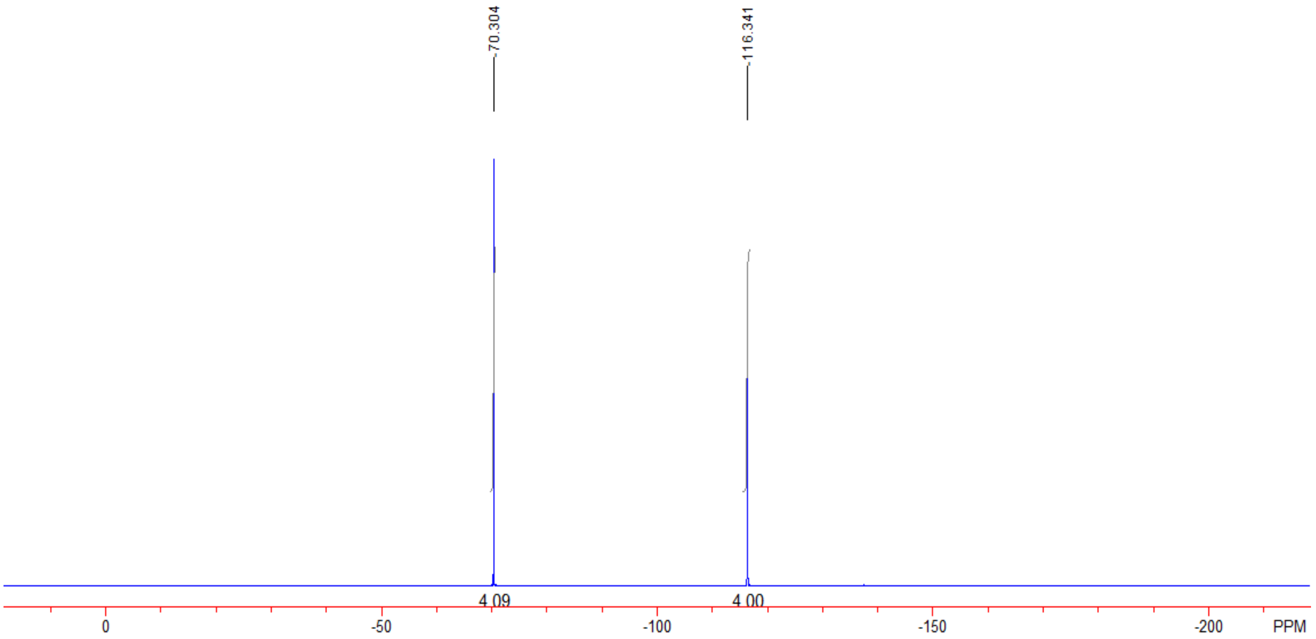


**Figure S11.** $^{19}F$ NMR spectrum of **4FCl-ZnI$_2$(II)** complex.

### 3. 2FCl-ZnI$_2$(III)

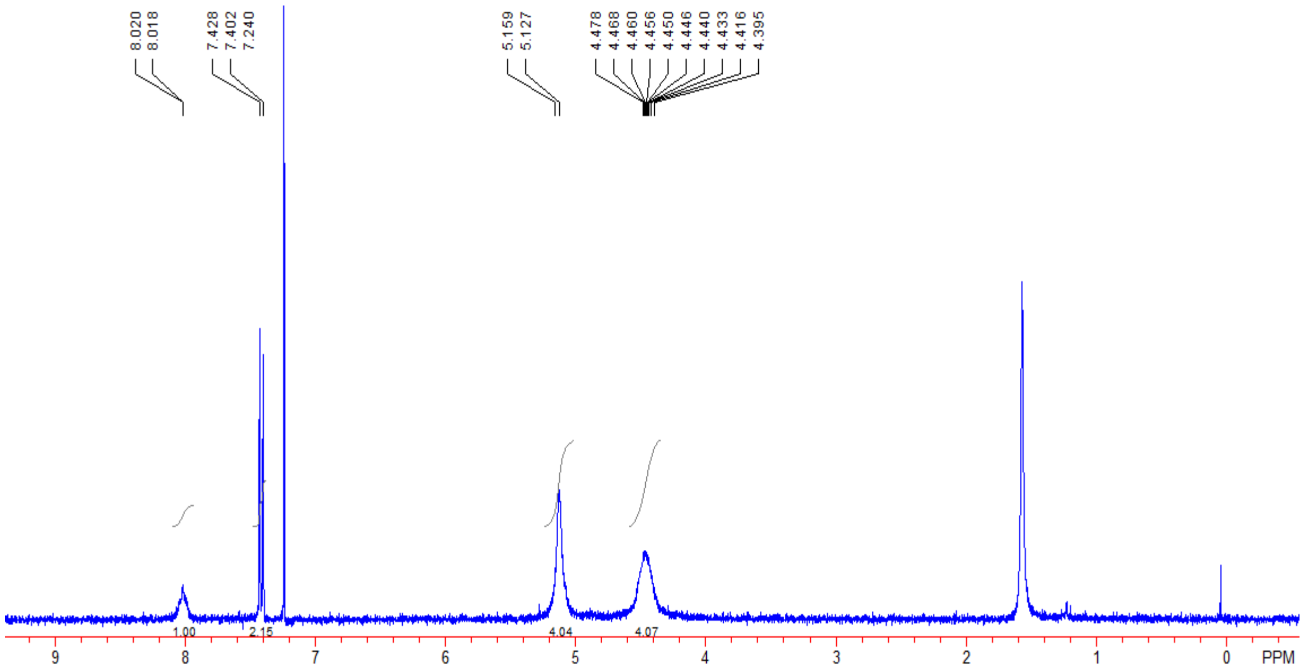


**Figure S12.** $^1$H NMR spectrum of **2FCl-ZnI$_2$(III)** complex.

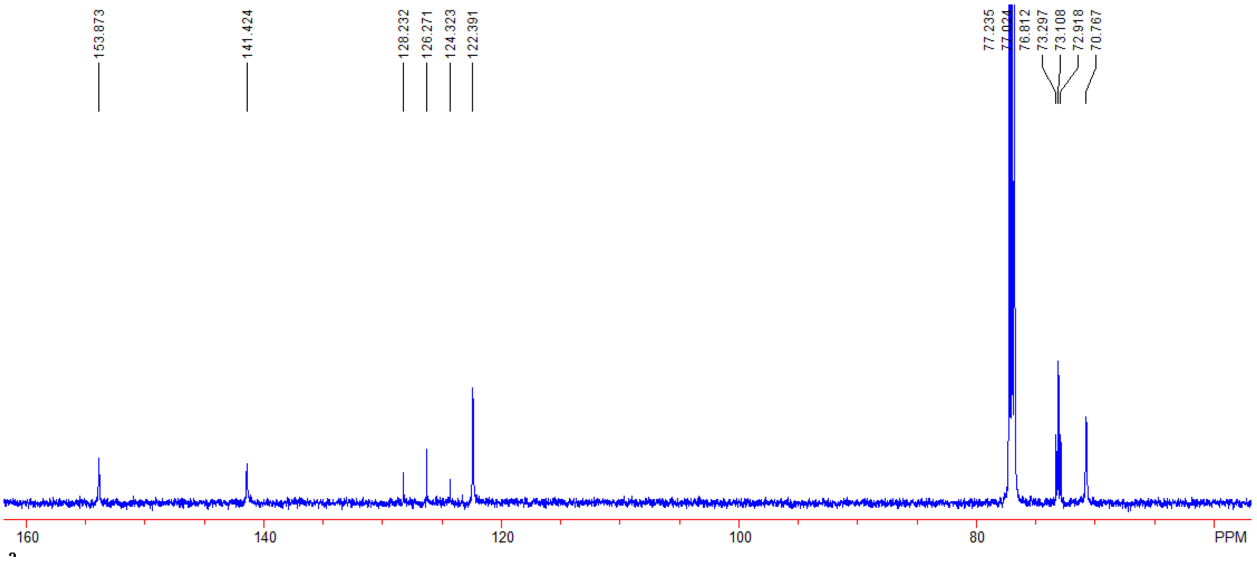


**Figure S13.** $^{13}$C NMR spectrum of **2FCl-ZnI$_2$(III)** complex.

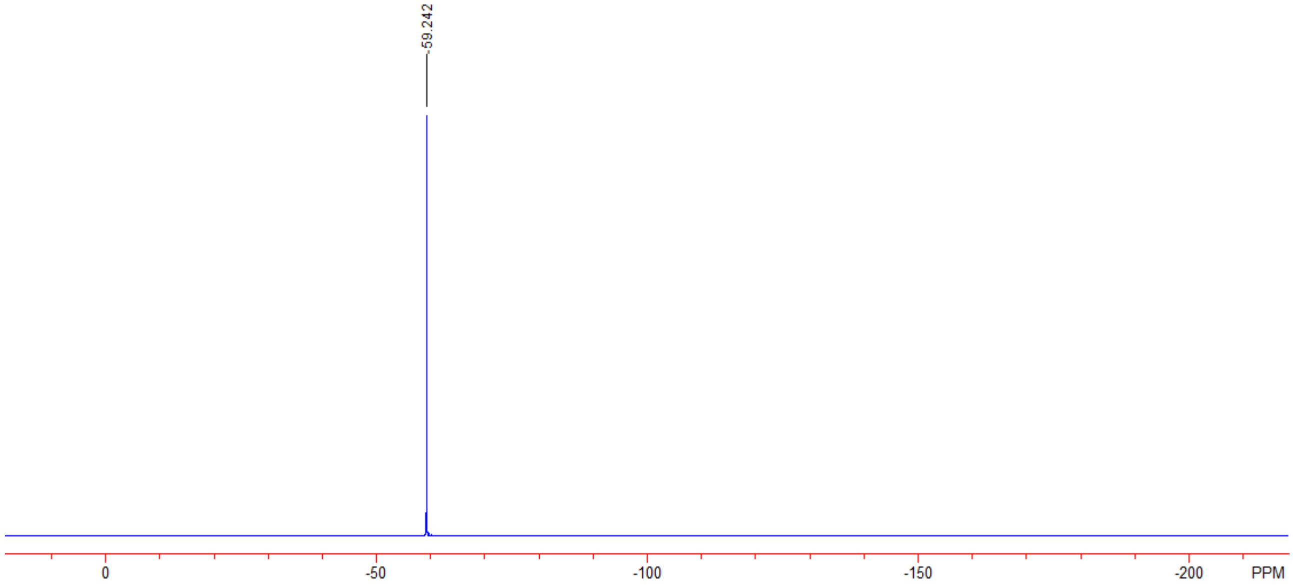


**Figure S14.** $^{19}$F NMR spectrum of **2FCl-ZnI$_2$(III)** complex.

## VI. References


1. Elakkat, V.; Tessema, E.; Lin, C.H.; Wang, X.; Chang, H.C.; Zheng, Y.N.; Huang, Y.C.; Gurumallappa; Zhang, Z.Y.; Long Chan, K.; Rahayu, H.A.; Francisco, J. S.; Lu, N. Unusual Changes of C– H Bond Lengths in Chiral Zinc Complexes Induced by Noncovalent Interactions. *Angew. Chemie Int. Ed.* **2023**, *62*, e202215438.

2. Coates, L.; Cao, H.B.; Chakoumakos, B.C.; Frontzek, M.D.; Hoffmann, C.; Kovalevsky, A.Y.; Liu, Y.; Meilleur, F.; dos Santos, A.M.; Myles, D.A.; Wang, X.P.; Ye, F. A suite-level review of the neutron single-crystal diffraction instruments at Oak Ridge National Laboratory. *Rev. Sci. Instrum.* **2018**, *89*, 092802.

3. Schultz, A.J.; Jørgensen, M.R.V.; Wang, X.; Mikkelson, R.L.; Mikkelson, D.J.; Lynch, V.E.; Peterson, P.F.; Green, M.L.; Hoffmann, C.M. Integration of neutron time-of-flight single-crystal Bragg peaks in reciprocal space. *J. Appl. Crystallogr.* **2014**, *47*, 915–921.

4. Zikovsky, J.; Peterson, P.F.; Wang, X.P.; Frost, M.; Hoffmann, C. CrystalPlan: an experiment-planning tool for crystallography. *J. Appl. Crystallogr.* **2011**, 44, 418–423.

5. Schultz, A.J.; Srinivasan, K.; Teller, R.G.; Williams, J.M.; Lukehart, C.M. Single-crystal, time-of-flight, neutron-diffraction structure of hydrogen cis-diacetyltetracarbonylrhenate,[cis-(OC) 4Re $(CH_3CO)_2$]H: a metallaacetylacetone molecule. *J. Am. Chem. Soc.,* **1984**, 106, 999-1003.

6. Sheldrick, G. M. *Acta Crystallogr. Sect. A Struct. Chem.* **2015**, 71, 3–8.

7. Hübschle, C.B.; Sheldrick, G.M.; Dittrich, B. ShelXle: a Qt graphical user interface for SHELXL. *J. Appl. Crystallogr.* **2011**, 44, 1281–1284.

8. Ohhara, T.; Kiyanagi, R.; Oikawa, K.; Kaneko, K.; Kawasaki, T.; Tamura, I.; Nakao, A.; Hanashima, T.; Munakata, K.; Moyoshi, T.; Kuroda, T. SENJU: a new time-of-flight single-crystal neutron diffractometer at J-PARC. *J. Appl. Crystallogr.* **2016,** *49,* 120-127.

9. Ohhara, T.; Kusaka, K.; Hosoya, T.; Kurihara, K.; Tomoyori, K.; Niimura, N.; Tanaka, I.; Suzuki, J.; Nakatani, T.; Otomo, T.; Matsuoka, S.; Tomita, K.; Nishimaki, Y.; Ajima, T.; Ryufuku, S. Development of data processing software for a new TOF single crystal neutron diffractometer at J-PARC. *Nucl. Instr. Meth. Phys. Res. Sect. A* **2009,** *600,* 195-197.

10. Rodríguez-Carvajal, J. Recent advances in magnetic structure determination by neutron powder diffraction. Phys. B: Condens. Matter **1993**,192, 55.

11. Dunitz, J. D.; Maverick, E. F.; Trueblood, K. N. Atomic motions in molecular crystals from diffraction measurements. *Angew. Chemie Int. Ed.* **1988**, 27, 880–895.

12. Schomaker, V.; Trueblood, K. N. Correlation of internal torsional motion with overall molecular motion in crystals. *Acta Crystallogr. Sect. B Struct. Sci.* **1998**, 54, 507–514.

13. Spek, A. L. Platon/squeeze, *Acta Crystallogr. Sect. D Biol. Crystallogr.* **2009**, 65, 148–155.

14. Farrugia, L. J. WinGX and ORTEP for Windows: an update. *J. Appl. Crystallogr.* ***2012***, 45, 849–854.

15. Frisch, M.J.; Head-Gordon, M.; Pople, J.A. A direct MP2 gradient method. *Chem. Phys. Lett.* **1990**, 166, 275-280.

16. Frisch, M. J.; Trucks, G. W.; Schlegel, H. B.; Scuseria, G. E.; Robb, M. A.; Cheeseman, J. R.; Scalmani, G.; Barone, V.; Petersson, G. A.; Nakatsuji, H.; Li, X.; Caricato, M.; Marenich, A. V.; Bloino, J.; Janesko, B.G.; Gomperts, R.; Mennucci, B.; Hratchian, H. P.; Ortiz, J. V.; Izmaylov, A. F.; Sonnenberg, J. L.; Williams-Young, D.; Ding, F.; Lipparini, F.; Egidi, F.; Goings, J.; Peng, B.; Petrone, A.; Henderson, T.; Ranasinghe, D.; Zakrzewski, V. G.; Gao, J.; Rega, N.; Zheng, G.; Liang, W.; Hada, M.; Ehara, M.; Toyota, K.; Fukuda, R.; Hasegawa, J.; Ishida, M.; Nakajima, T.; Honda, Y.; Kitao, O.; Nakai, H.; Vreven, T.; Throssell, K.; Montgomery, J. A., Jr.; Peralta, J. E.; Ogliaro, F.; Bearpark, M. J.; Heyd, J. J.; Brothers, E. N.; Kudin, K. N.; Staroverov, V. N.; Keith, T. A.; Kobayashi, R.; Normand, J.; Raghavachari, K.; Rendell, A. P.; Burant, J. C.; Iyengar, S. S.; Tomasi, J.; Cossi, M.; Millam, J. M.; Klene, M.; Adamo, C.; Cammi, R.; Ochterski, J.W.; Martin, R. L.; Morokuma, K.; Farkas, O.; Foresman, J. B.; Fox, D. J. Gaussian, Inc.: Wallingford, CT, **2016**.

17. Dennington, R.; Keith T.; Millam, J. GaussView, Version 6.1.1, Semichem Inc., Shawnee Mission, KS, **2019**.

18. Hay, P. J.; Wadt, W. R. Ab initio effective core potentials for molecular calculations. Potentials for K to Au including the outermost core orbitals. *J. Chem. Phys.* **1985**., 82, 299-310.

19. Krishnan, R.B.J.S.; Binkley, J.S.; Seeger, R.; Pople, J.A. Self-consistent molecular orbital methods. XX. A basis set for correlated wave functions. *J. Chem. Phys.* **1980**,72, 650-654.
