# Supplementary material for: Correction to “Neutron Diffraction and Spectroscopic Studies of Intramolecular Tetrel Bonds in Three Fluorinated Zinc Complexes: Significant Redshift in the sp3 C–H Stretch Confirmed by Experiments and Theory”
Source: J Am Chem Soc. 2026 Jan 14;148(3):3909. doi: 10.1021/jacs.5c22553 (PMC12856883; doi:10.1021/jacs.5c22553)
Supplement: Supplementary file 1 [file ja5c22553_si_001.pdf]

Neutron Diffraction and Spectroscopic Studies of Intramolecular Tetrel Bonds in Three Fluorinated Zinc Complexes: Significant Red-shifting in the sp^3 C−H Stretch Affirmed by Experiment and Theory

Norman Lu,[1,2]* Gurumallappa Gurumallappa,[1,2] Pin-Yu Liu,[1] Ka-Long Chan,[1] Yu-Cheng Huang,[1] Yu-Ching Lin,[1] Yun-Ting Hsieh,[1] Pin-Xiang Zeng,[1] Yashwanth Gowda,[1] Meng-Hsun Tsai, Eskedar Tessema,[1] Huan-Cheng Chang,[3] Joseph S. Francisco[4]*

1. Institute of Organic and Polymeric Materials, National Taipei University of Technology, Taipei 106, Taiwan (ROC). E-mail: normanlu@mail.ntut.edu.tw
2. Graduate Institute of Energy and Optoelectronic Materials, National Taipei University of Technology, Taipei 106, Taiwan (ROC).
3. Institute of Atomic and Molecular Sciences, Academia Sinica, Taipei 106, Taiwan (ROC).
4. Department of Earth and Environmental Science and Department of Chemistry, University of Pennsylvania, Philadelphia, Pennsylvania 19104-6316, United States. E-mail: frjoseph@sas.upenn.edu

Table of Contents

I. Experimental procedures

Single crystal X-ray diffraction studies .... SI 4
Single crystal neutron diffraction studies .... SI 4
Computational methods .... SI 5

II. Supporting scheme

Scheme S1**.** Schematic representation of tetrel bond and C-H···F improper H-bond .... SI 7
Scheme S2**.** The image of a cyclohexane-like chair form .... SI 8
Scheme S3**.** The Schematic representation of Hooke's Law .... SI 8

III. Supporting figures of (A) neutron structures and (B) the vibrational spectra of two methylene C-H bonds under local mode

Figure S1. ORTEP diagram of Neutron structure of complex **4FH-ZnCl$_2$(I)** .... SI 9
Figure S2. ORTEP diagram of Neutron structure of complex **4FCl-ZnI$_2$(II)** .... SI 10
Figure S3. ORTEP diagram of Neutron structure of complex **2FCl-ZnI$_2$(III)** .... SI 11
Figure S4. Raman spectrum and FT-IR spectrum of the deuterated **2FCl-ZnI$_2$(III)** .... SI 12
Figure S5. Theoretical linear plot of for complexes **I-III** .... SI 13
Figure S6A. Linear plot of calculated sp^3 C-H bond force constant vs its bond distance (based on the local mode vibrational theory by using PyMOL software) .... SI 14
Figure S6B. The diagram with blue and red lines for complex **I-III** .... SI 14
Figure S7. Characterization of normal mode (CNM) plots for complex **I-III** .... SI 15
Figure S8. The calculated vibrational mode vector for complex **I-III** .... SI 16

IV. Structural table section

Table S1. Crystallographic data refinement parameters table for **4FH-ZnCl$_2$(I)** .... SI 17
Table S2. Weak interactions (w/o libration correction) in **4FH-ZnCl$_2$(I)** .... SI 18
Table S3. Weak interactions (with libration correction) in **4FH-ZnCl$_2$(I)** .... SI 18
Table S4. Selected bond lengths and angles (w/o libration correction) for **4FH-ZnCl$_2$(I)** .... SI 19
Table S5. Selected bond lengths and angles (with libration correction) for **4FH-ZnCl$_2$(I)** .... SI 20
Table S6. Crystallographic data refinement parameters table for **4FCl-ZnI$_2$(II)** .... SI 21
Table S7. Weak interactions of tetrel bond and improper HB in **4FCl-ZnI$_2$(II)** .... SI 22
Table S8. Selected bond lengths and angles for **4FCl-ZnI$_2$(II)** .... SI 23

Table S9. Crystallographic data refinement parameters table for **2FCl-ZnI2(III)** SI 24
Table S10. Weak interactions (w/o libration correction) in **2FCl-ZnI2(III)** SI 25
Table S11. Weak interactions of (with libration correction) in **2FCl-ZnI2(III)** SI 25
Table S12. Selected bond lengths and angles (w/o libration correction) for **2FCl-ZnI2(III)** SI 26
Table S13. Selected bond lengths and angles (with libration correction) for **2FCl-ZnI2(III)** SI 26
Table S14. Experimental data of the wavenumber vs. neutron C–H bond length SI 27
Table S15. Deuterated experimental data of the wavenumber vs. neutron C–H bond length SI 27
Table S16. Calculated C–H bond length and ist force constant using by using PyMOL SI 27

V. Atomic coordinates

Table S16-24. Atomic coordinates of complexes **I-III** using MP2 method SI 28
Table S25-27. Atomic coordinates of complexes **I-III** using DFT method SI 37

VI. Synthesis of deuterated compounds

Synthesis of deuterated compounds SI 40

VII. NMR spectra of complexes I-III SI 43

VIII. NMR spectra of deuterated complexes I-III SI 48

IX. References SI 51

I. Experimental procedures

Single Crystal X-ray Diffraction Studies

Data were collected using a Rigaku XtaLAB Synergy DW single crystal diffractometer equipped with a HyPix-Arc 150° curved Hybrid Photon Counting X-ray detector and MicroMax-007 HF microfocus rotating anode with dual wavelength (Cu and Mo). Data processing was carried out using the Bruker software package, and structure solution and refinement were carried out with the SHELXS routines. All H atoms were calculated and treated with a riding model. The H atom isotropic displacement parameters were defined as 1.2 Ueq of the adjacent atom.

Single Crystal Neutron Diffraction Studies of Complex (I), (III).

Single crystal neutron diffraction data for fluorinated [(**4FH-ZnCl$_2$(I**) and **2FCl-ZnI$_2$(III**) metal complexes were determined on the TOPAZ single−crystal time−of−flight (TOF) Laue diffractometer at the Spallation Neutron Source, Oak Ridge National Laboratory (Tennessee, USA).[1-3] The diffractometer had 18 detectors installed, each with an active area of 15 cm x 15 cm, and they were arranged on a near−spherical detector array tank. The initial moderator−to−sample flight path was 18 m, and the sample−to−detector distances varied in the range 39−46 cm. The total path length of 18.4 m and the SNS pulse rate of 60 Hz provided a wavelength bandwidth of 3.6 Å. Each crystal of complexes (**I, III**), with the dimension, [2.40×1.80×1.60 (**I**), 2.25 × 1.55 × 1.50 (**III**) specified in its neutron cif, was mounted on the tip of a MiTeGen loop using Super Glue and cooled to 100 K for data collection. Total of 40 crystal orientations optimized with CrystalPlan[4] software were used to ensure better than 95% coverage of a hemisphere of reciprocal space. Each orientation was measured for 6 − 7 coulombs of proton−charge for approximately 1.5 h with the SNS operated at 1.4 MW. The integrated raw Bragg intensities were obtained using 3−D ellipsoidal Q−space integration in accordance with previously reported methods.[5] Data reduction, including the neutron TOF spectrum, Lorentz and detector efficiency corrections, was carried out with the ANVRED3 program.[6] A Gaussian numerical absorption correction was applied with $\mu = 0.11327 + 0.07311\lambda$ mm^{-1}. The reduced data were saved in SHELX HKLF2 format, where the wavelength was recorded separately for each individual reflection and not merged. Non-hydrogen atom positions from the X−ray structure were used as the starting model for the refinement of the neutron structure. Refinement was performed using the SHELXL−2018/3 program interfaced with ShelXle.[6,7] Hydrogen atoms were located from the difference Fourier map and refined anisotropically to convergence.

Single Crystal Neutron Diffraction Studies of Complex (II).

Single crystal neutron diffraction data for fluorinated **4FCl-ZnI$_2$(II**) metal complex was taken using SENJU single−crystal time−of−flight (TOF) Laue neutron diffractometer[8] installed at BL18 at the Materials and Life Science Experimental Facility (MLF) of Japan Proton Accelerator Research Complex (J-PARC) Japan. The wavelength range of incident neutrons was 0.4 to 4.4 Å. A block−shaped crystal of complex (**II**), with dimensions of 2.50 mm × 2.50 mm × 1.00 mm, was mounted on the top of an aluminum pin using an epoxy glue and attached to a fixed-χ type two-axes (ω, φ) goniometer. Then, the diffraction data was acquired at 40 K under vacuum conditions.

Intensities of Bragg peaks were collected using 41 two-dimensional scintillation detectors with 24 crystal orientations. The exposure time for one crystal orientation was 3 hours. The accelerator power was 800 kW. Data reduction was performed using the STARGazer[9,10] to obtain hkl indexes and the corresponding integrated intensities of reflections corrected for the detector efficiency, Lorentz factor, and scaling factor for each crystal orientation. A spherical absorption correction was applied with $\mu = 0.198 + 0.00874\lambda$ mm^{-1}. The reduced data were saved in SHELX HKLF2 format, where the wavelength was recorded separately for each individual reflection. Non-hydrogen atom positions from the X–ray structure were used as the starting model for the refinement of the neutron structure. Refinement was performed using the SHELXL–2018/3 program interfaced with ShelXle.[6,7] Hydrogen atoms were located from the difference Fourier map and refined anisotropically to convergence. The effects of libration on the molecular geometry of the complexes (**I**) and (**III**) were corrected using the THMA14c program[11-13] in WinGX.[14] The neutron diffraction data obtained at 40 K for complex (**II**) has been directly for further analysis.

Notes

Deposition numbers 2351846 [4FH-$ZnCl_2$(**I**)], 2351847 [4FCl-ZnI_2(**II**)] and 2351848 [2FCl-ZnI_2(**III**)] contain the supplementary crystallographic x-ray data for this paper. The neutron diffraction data (without libration correction) for complexes (**I**), (**II**) and (**III**), are provided as 2352173, 2352174 and 2352175, respectively. The neutron diffraction data (with libration correction) for complexes (**I**) and (**III**) are provided as 2352217 and 2352218, respectively. These data are provided free of charge by the joint Cambridge Crystallographic Data Center.

Computation Methods

In Section B1

All quantum-chemical calculations were performed using the unrestricted second-order Møller-Plesset perturbation (MP 2)[15] method by the Gaussian 16[16] program and output files are visualized by its GaussView 6.0.16 program.[17] Basis sets of LANL2DZ[18] was utilized to describe the all elements (such as H, C, N, O, F, Cl, I, and Zn atoms). MP2 method was found to calculate the C-H bond length better than DFT method.

Note 1. The optimized atomic coordinates of complexes (**I–III**) are given in Table S16-24.

Note 2. All local mode calculations for complexes (**I–III**) were verified by analyzing their vibrational spectra using Gaussian 16 to validate the computational model. The simulated IR spectra align with experimental data; and all optimized structures exhibit only positive vibrational frequencies. Additionally, the calculated IR frequencies match the experimental values.

In Section B2

All quantum-chemical calculations were performed by using the Gaussian 16 program.[16]

Local vibrational mode analyses of complexes **I–III** were performed using LmodeAnano[22], plugin in PyMOL software[23] which implements the LModeA approach for generating local mode parameters from vibrational frequency calculations.[23,24] By using PyMOL software, we obtained bond-specific local vibrational frequencies and force constants, which could provide the reliable measurement of the intrinsic bond strength.

For CNM (composition of normal modes)[25] approach, the stretching frequency calculations are performed based on both MP2[15] and DFT (ωB97X-D)[20] methods; and LANL2DZ[18] and aug-cc-pVDZ[21] were used as the basis sets, respectively.

Note a. Both MP2 and DFT methods have been used here. Both MP2 and DFT methods do not use the frozen electron in the treatment. The MP2 method is the unrestricted second-order Møller-Plesset perturbation method, so it is unrestricted MP2 method.

Note b. The optimized atomic coordinates of complexes (**I–III**) using ωB97X-D/aug-cc-pVDZ are given in Table S25-27.

In Section B3

Mainly is based on **Cremer-Kraka criterion**, so the nature of blue-shifting HB and TB, which were calculated using PyMOL software, were then analyzed according to Cremer-Kraka criterion.

In Section B4

All quantum-chemical calculations for NCI and NBO analyses were performed using DFT method at the B3LYP level[25] by the Gaussian 16 program[16] with D3BJ dispersion correction.[26] And Gaussview 6.0.16[17] were used to visualize the output files.

The noncovalent interactions (**NCI**) calculations of **I-III** were analyzed under B3LYP/aug-cc-pVTZ level of theory with D3BJ dispersion corrections and validated within (QTAIM) with the help of Multiwfn 3.8.[29] The outputs were visualized by the Visual Molecular Dynamics (VMD) program.[30]

The **NBO** plot with D3BJ dispersion corrections on **I** was optimized using B3LYP/6-31G(d,p)[25] or higher level of theory.

The **NBO** plots with D3BJ dispersion corrections on **II** and **III** were optimized using B3LYP/GenECP, where the 6-311G(d,p) basis set was applied for normal main-group elements and effective core potentials (ECPs) together with other corresponding basis set being used for I (heavy element).

II. Supporting scheme

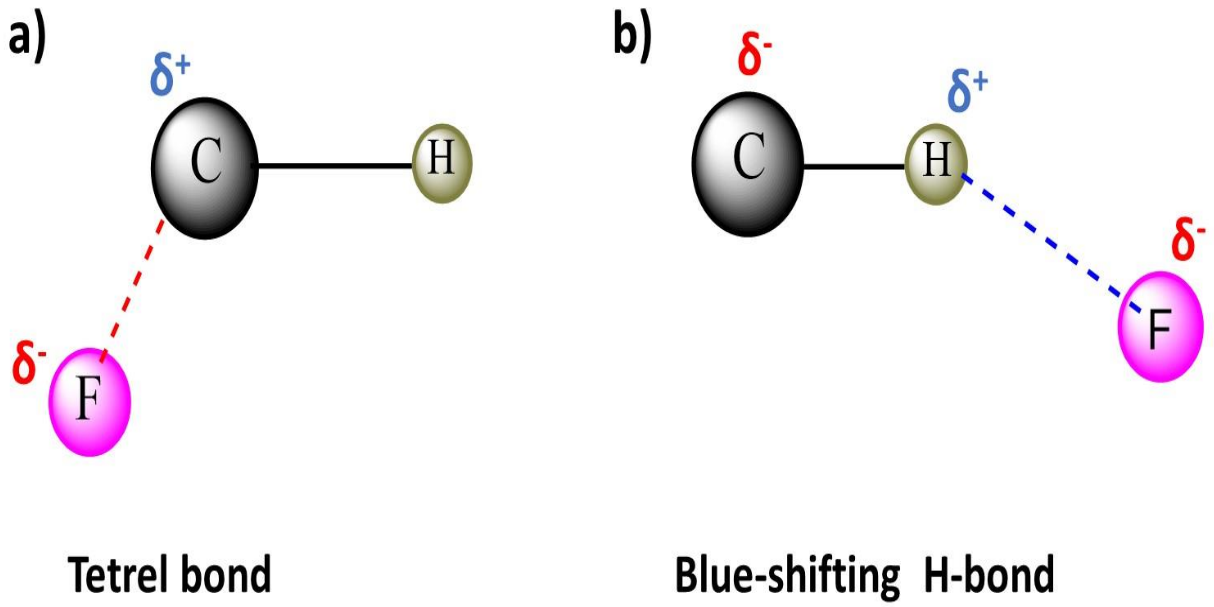

Scheme S1. Schematic representation of a) tetrel bond (TB) and b) C-H···F improper H-bond (HB).

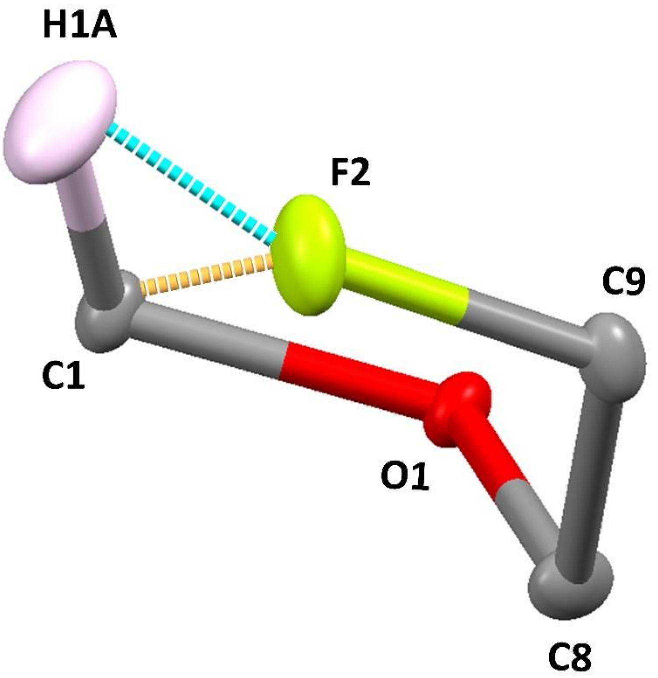

Scheme S2. The image of a cyclohexane-like chair form.

[**Note.** The intramolecular (C1···F2) TB, within a 6-membered chair form.]

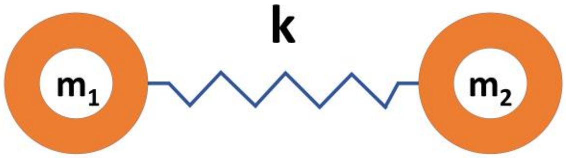

$$\overline{\nu} = \frac{1}{2\pi c}\sqrt{\frac{k}{\mu}} \quad \text{..... eq (s1)}$$

$$\mu = \frac{m_1 \cdot m_1}{m_1 + m_1} \quad \text{..... eq (s2)}$$

Scheme S3. The Schematic representation of Hooke's Law.

(Note. The wavenumber equation and reduced mass expression are shown.)

III. (A) Supporting figures of neutron structure

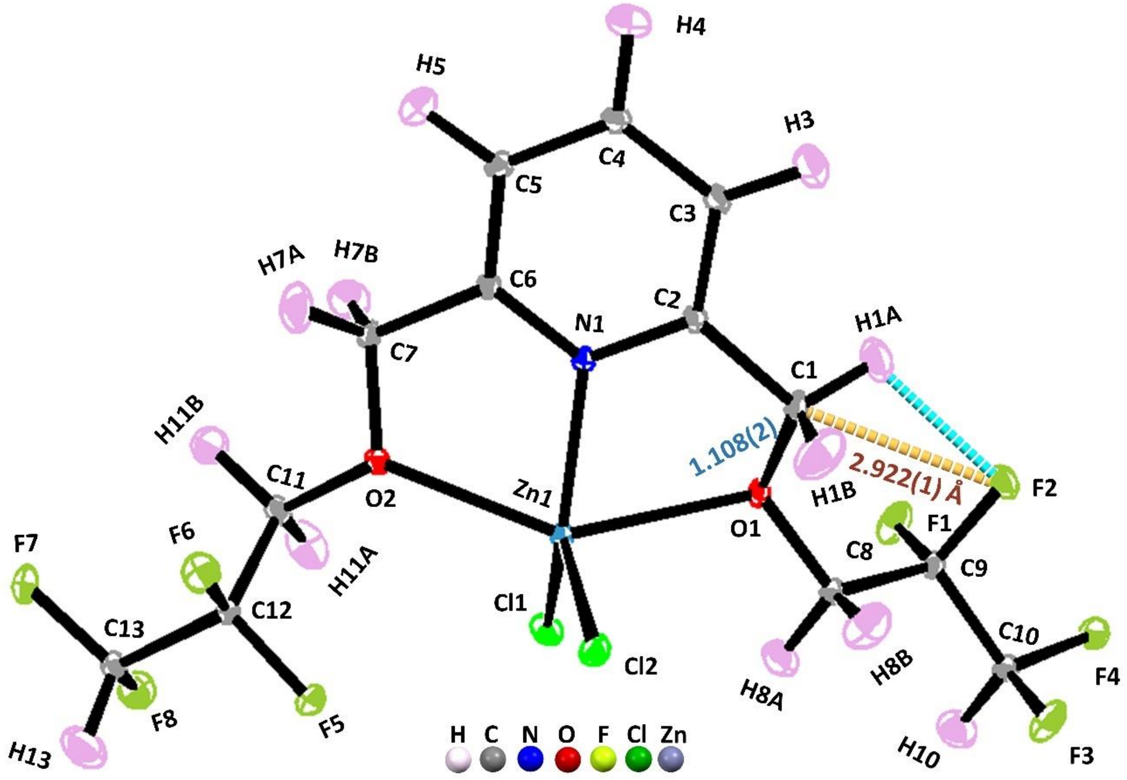

Figure S1. ORTEP of **4FH-ZnCl$_2$(I)** neutron structure and its drawing shows the intramolecular TB. [d(C···F)=2.922(1) Å, angle:150.5°; C1-H1B=1.108(2) and C1-H1A=1.092(1) Å].

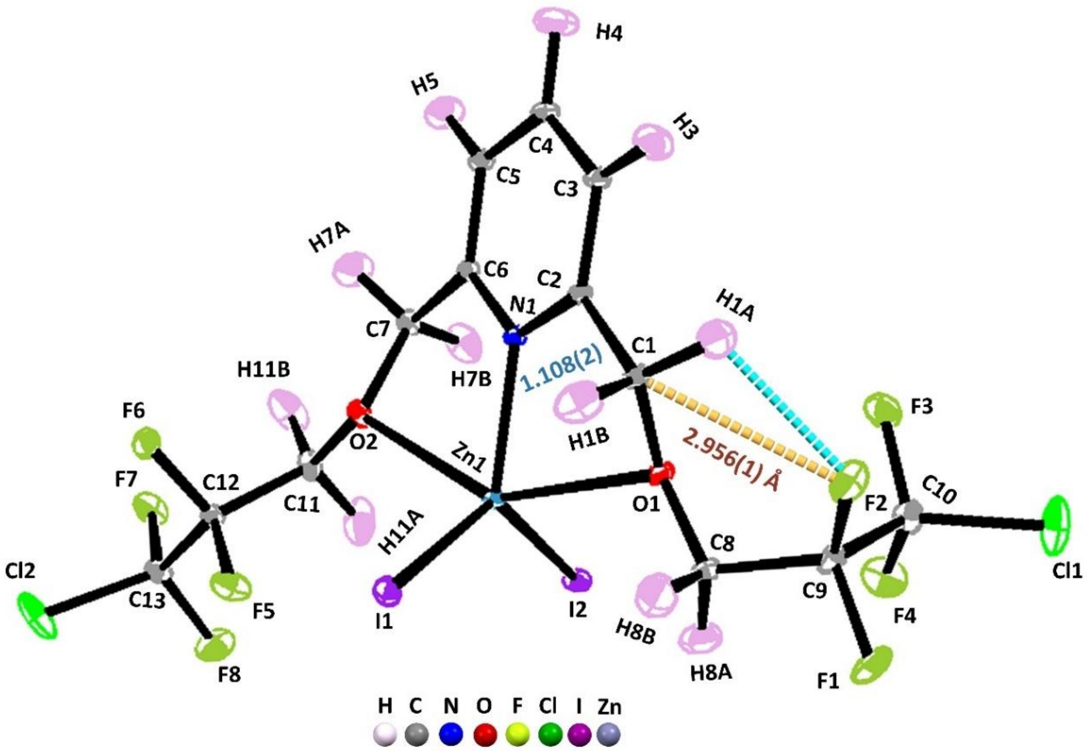

Figure S2. ORTEP of **4FCl-ZnI$_2$(II)** neutron structure and its drawing shows the intramolecular TB. [d(C⋯F)=2.956(1) Å, angle:153.2°; C1-H1B=1.108(2) and C1-H1A=1.095(4) Å]. [Note. This structure was measured in J-PARC at 40 K in Japan.]

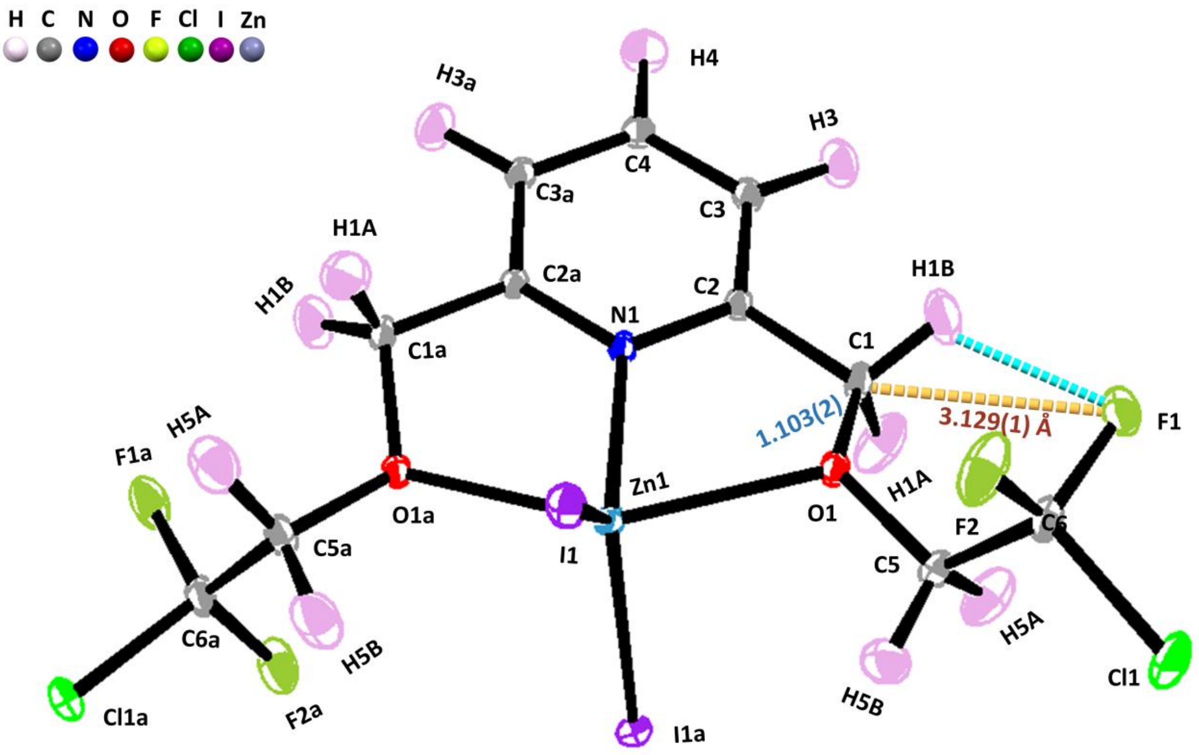

Figure S3. ORTEP of **2FCl-ZnI$_2$(III)** neutron structure with the intramolecular TB indicated. Note. The drawing of **2FCl-ZnI$_2$(III)** shows the intramolecular TB, [d(C⋯F)=3.129(1) Å, angle:143.8°; C1-H1A=1.103(2) and C1-H1B=1.093(1) Å].

(B) Supporting vibrational spectrum

Vibrational spectra of 2FCl-ZnI2(III) and its deuterated analogue

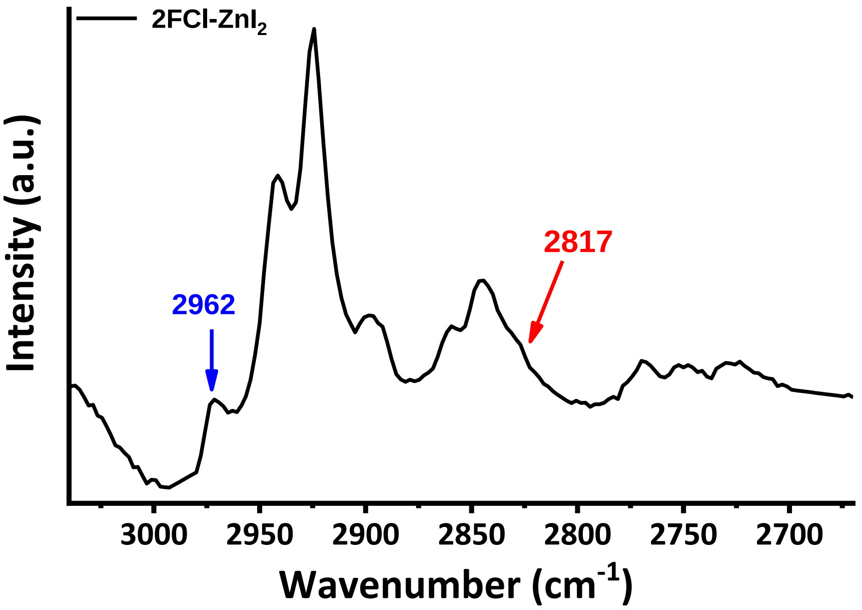

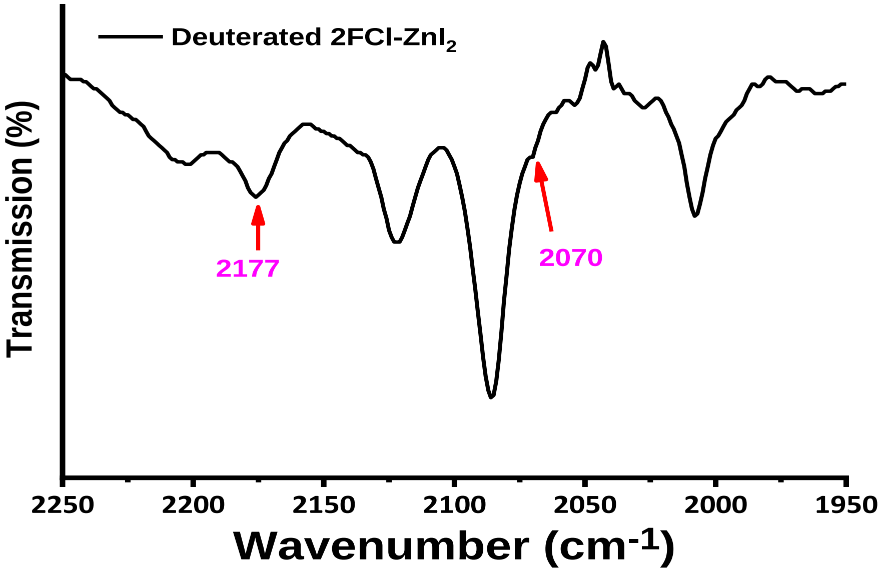

Figure S4. Vibrational spectra of 2FCl-ZnI2(**III**) and its deuterated analogue. a) Raman spectrum and b) FT-IR spectrum of the deuterated complex **III** species. [Note: k1 (2962/2177) = 1.36 & k2 (2817/2070) = 1.36; so **k1=k2=1.36**].

Theoretical linear plot

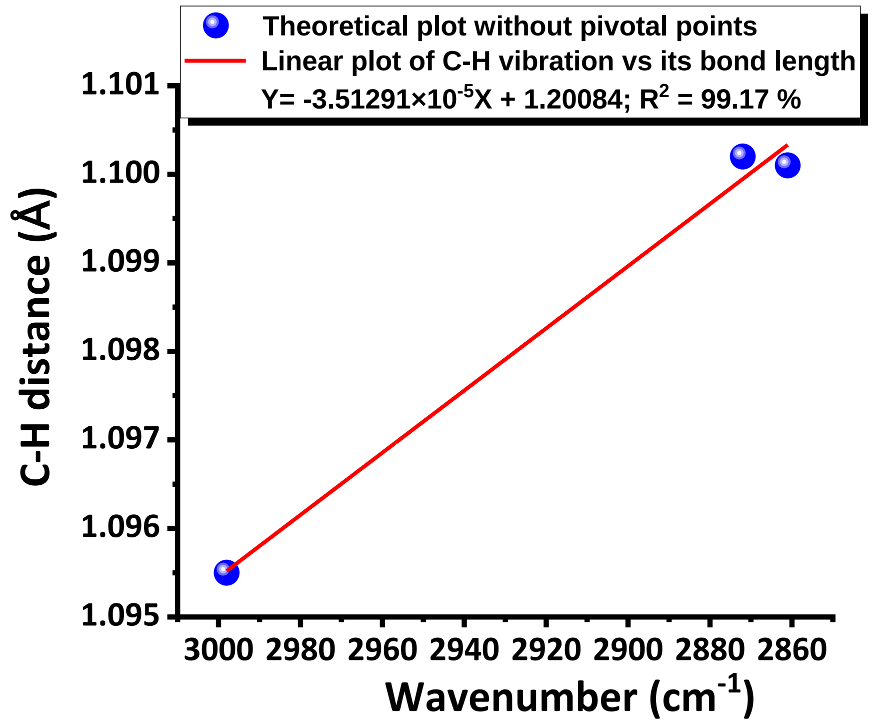

Figure S5. The theoretical linear plot of C-H vibration vs its bond length without pivotal points for complexes **I-III**.

Figure S6 Plot

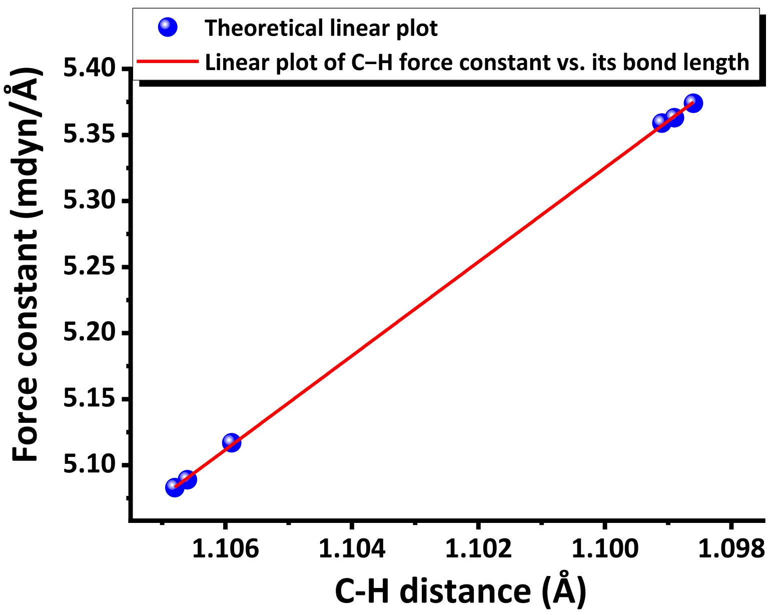

Figure S6A. Linear plot of calculated sp^3 C-H bond force constant vs its bond distance based on the local mode vibrational theory. (By using PyMOL software; Y=−35.5578·X + 44.43865; R^2= 99.99%.

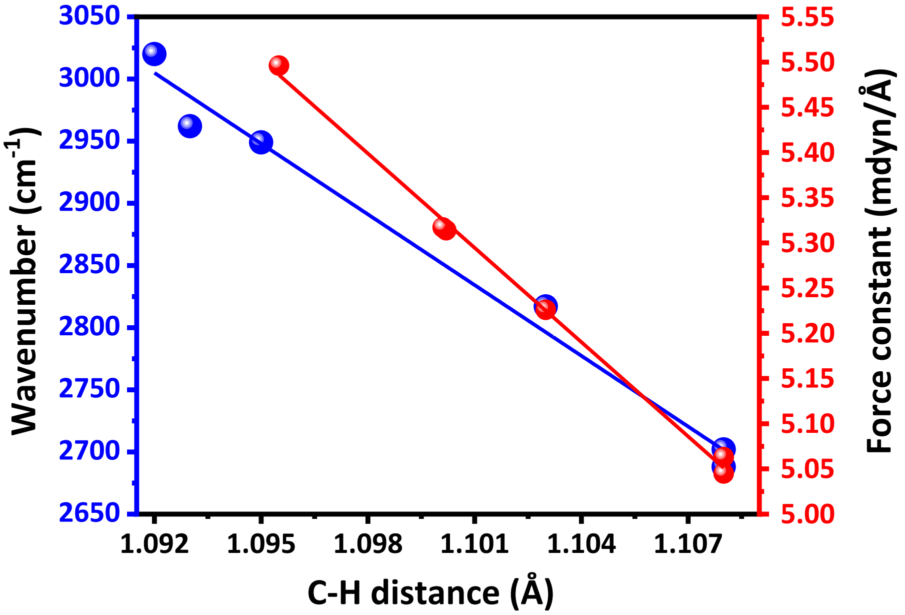

Figure S6B. The diagram with blue and red lines for complex **I-III;** which has one red line plot is the force constant vs C-H distance and the other blue line plot (i.e., experimental plot of Fig. 7) are then plotted together.

Note: Figure 7, which has the C-H distance in x-axis herein, is almost the same as Figure 11 (from the previous submission) except the x and y axes being swapped.

Composition of normal mode (CNM) plots for complex I-III

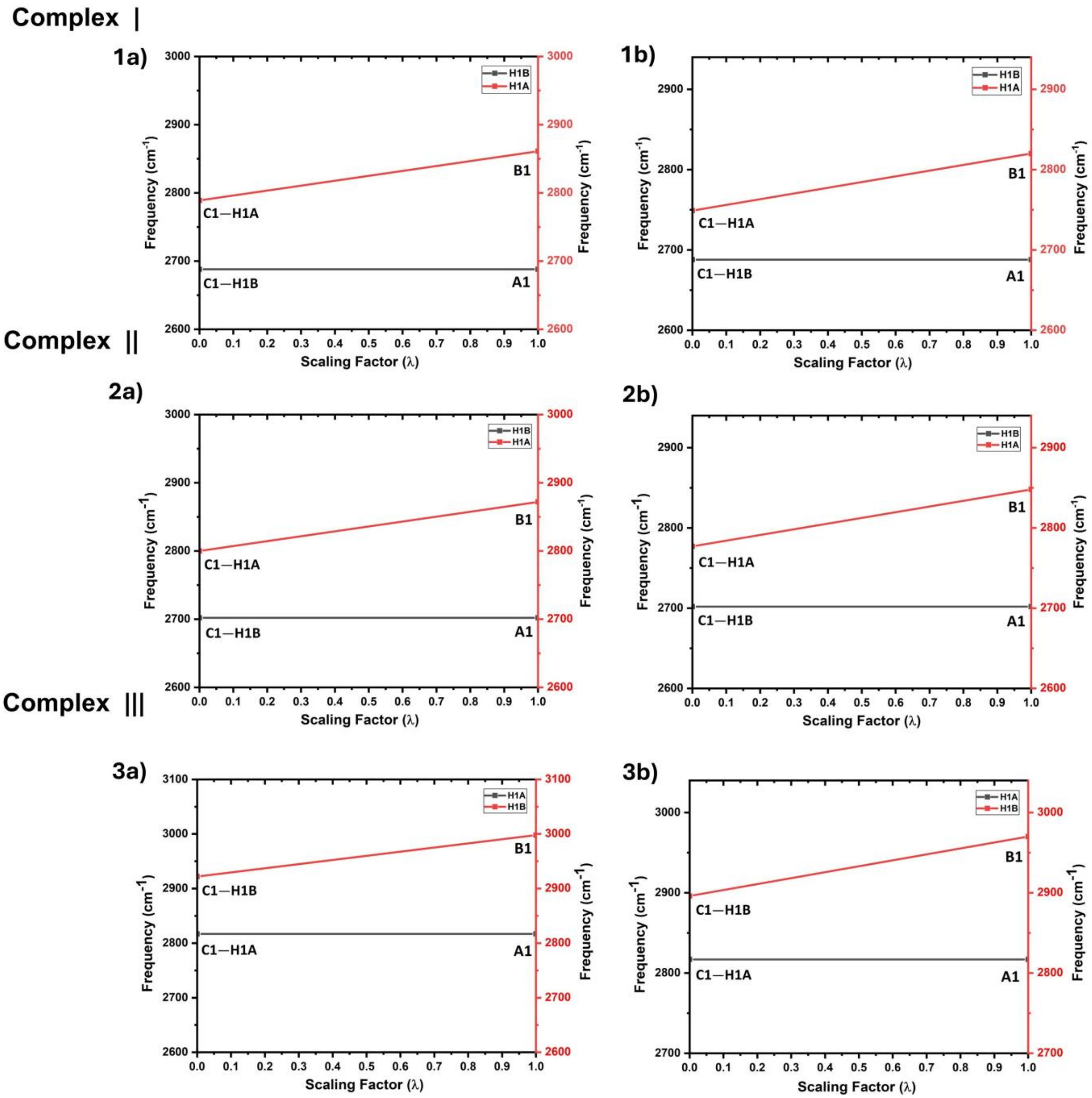

Figure S7. Using the CNM approach (by MP2 and DFT methods), the comparison plots of the local mode vs the normal mode of complexes **I-III**. 1a-3a are by MP2 method of complexes **I-III**, respectively; and 1b-3b are by DFT (ωB97X-D/aug-cc-pvDz) method of complexes **I-III**, respectively.

As shown in Figure S7, the comparisons of the stretching frequencies of the local mode vs the normal mode of complexes **I-III** are plotted. The left and right columns are by using MP2 and DFT (ωB97X-D methods, respectively. The 1a-3a are by MP2 method for complexes **I-III**, respectively; and 1b-3b are by DFT (ωB97X-D/aug-cc-pvDz) method for complexes **I-III**, respectively. Take for example 1a & 1b of complex **I**, whose H-C-H moiety in the theoretically deuterated **4FH-ZnI$_2$(I)** has the C_{2v} point group, two methylene C-H stretching frequencies in 1a,b are so far apart that both C1-

H1A and C1-H1B stretching peaks does not couple with each other and has no effect from mass factor coupling at the local mode side (left). Furthermore, these two methylene C-H stretching frequencies have slightly larger difference in wavenumbers due to anharmonic vibration at the normal side (right).

In addition, for the top two plots of complex **I**, on the normal mode, the higher and lower stretching frequencies have the symmetry code of B1 and A1, respectively. Both 1a (by MP2 method) and 1b (by DFT method) plots look very similar. Besides complex **I**, the comparing C-H stretching plots (2a,b & 3a,b) of complexes **II, III** also show the same trends and similar results. Therefore, by using the CNM approach, the local mode of two methylene C-H stretching frequencies of complexes **I-III** has also been verified by using MP2 or DFT (ωB97X-D) calculations shown in Figure S7.

Calculated vibrational mode vector for complex I-III.

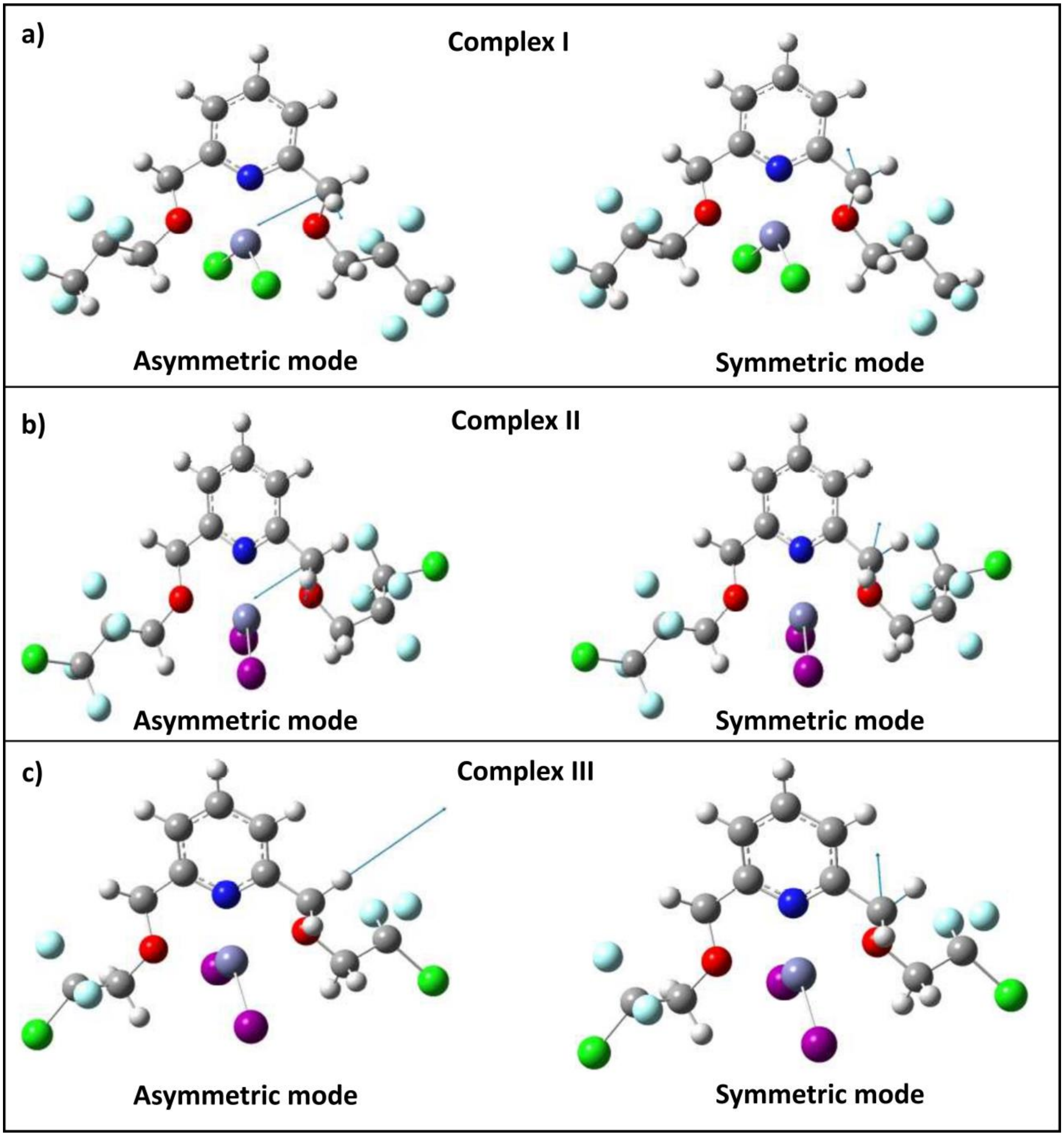

Figure S8. The calculated vibrational mode vector for a) complex **I**, b) complex **II** and c) complex **III**.

IV. Structural table section

Table S1. Crystallographic data and refinement parameters for **4FH-ZnCl₂(I)** complex.

	**X-ray diffraction CCDC ID: 2351846**	**Neutron diffraction CCDC ID: 235221**
Crystal data		
Chemical formula	$C_{13}H_{13}Cl_2F_8NO_2Zn$	$C_{13}H_{13}Cl_2F_8NO_2Zn$
M_r	503.51	503.51
Crystal system, space group	Triclinic, $P\bar{1}$	Triclinic, $P\bar{1}$
Temperature (K)	150	100
a, b, c (Å)	8.1118 (5), 11.1532 (6), 11.7262 (7)	8.0922 (3), 11.1333 (3), 11.6641 (3)
α, β, γ (°)	113.180 (1), 107.810 (2), 98.124 (2)	113.041 (2), 107.686 (3), 98.282 (3)
V (Å^3)	886.03 (9)	878.90 (5)
Z	2	2
Radiation type	Mo Ka	Neutron, l = 0.800 Å
m (mm^{-1})	1.78	0.1622 + 0.07163λ
Crystal size (mm)	0.75 × 0.63 × 0.51	2.40 × 1.80 × 1.60
Data collection		
Diffractometer	XtaLAB Synergy R, DW system, HyPix-Arc 150	TOPAZ
Absorption correction	Multi-scan[a]	Gaussian[b]
T_{min}, T_{max}	0.349, 0.464	0.505, 0.771
No. of measured, independent and observed [I > 2s(I)] reflections	24880, 3164, 3036	34267, 8113, 7369
R_{int}	0.042	0.076
(sin q/l)$_{max}$ (Å^{-1})	0.601	1.225
Refinement		
$R[F^2 > 2s(F^2)]$, $wR(F^2)$, S	0.024, 0.061, 1.08	0.029, 0.065, 1.01
No. of reflections	1769	8113
No. of parameters	107	362
H-atom treatment	H-atom parameters constrained	All H-atom parameters refined
$\Delta\rho_{max}$, $\Delta\rho_{min}$	0.64, -0.81(e Å^{-3})	0.90, -0.91(fm Å^{-3})

a) Bruker SADABS, 1996. (b) P. Coppens, L. Leiserowitz, D. Rabinovich, Acta Cryst. 18, 1035-1038 (1965).

Table S2. Weak interactions of tetrel bond and improper HB in **4FH-ZnCl$_2$(I)** complex [Å and °] (without libration correction).

Item no	a) Tetrel bond (O–C···F–C) in 4FH-ZnCl$_2$(I)				
	**H/C—C···F—C**	**H/C—C**	**C···F**	**H/C···F or C···C**	**∠CFC**
1	C2–C1···F2–C9	C2–C1= 1.506(1)<br>F2–C9 = 1.354(1)	C1···F2=2.922(1)	C2···F2= 4.297(1)<br>C1···C9= 3.135(1)	∠C2C1F2= 150.5(0)<br>∠C9F2C1= 86.1(0)
	**b) Improper hydrogen bonds (C–H···*A*)**				
	D*—H···*A	***D*—H**	**H···*A***	***D*···*A***	**∠*D*H*A***
2	C1—H1A···F2	1.091(1)	2.323(2)	2.922(1)	112.6(1)

Table S3. Weak interactions of tetrel bond and improper HB in libration-corrected **4FH-ZnCl$_2$(I)** complex [Å and °].

Item no	a) Tetrel bond (O–C···F–C) in 4FH-ZnCl$_2$(I)				
	**H/C—C···F—C**	**H/C—C**	**C···F**	**H/C···F or C···C**	**∠CFC**
1	C2–C1···F2–C9	C2–C1= 1.507(1)<br>F2–C9 = 1.355(1)	C1···F2=2.922(1)	C2···F2= 4.298(1)<br>C1···C9= 3.135(1)	∠C2C1F2= 150.5(0)<br>∠C9F2C1= 86.1(0)
	**b) Improper hydrogen bonds (C–H···*A*)**				
	D*—H···*A	***D*—H**	**H···*A***	***D*···*A***	**∠*D*H*A***
2	C1—H1A···F2	1.092(1)	2.323(2)	2.922(1)	112.6(1)

Table S4. Selected bond lengths (Å) and bond angles (°) for monomeric **4FH-ZnCl$_2$(I)** complex (without libration correction).

**Bond length (Å)**			
**Bond**	**Bond length (Å)**	**Bond**	**Bond length (Å)**
F5—C12	1.363(1)	C8—H8A	1.098(1)
F6—C12	1.354(1)	C3—H3	1.085(1)
F4—C10	1.346(1)	C7—H7A	1.093(1)
F8—C13	1.351(1)	C7—H7B	1.095(1)
F2—C9	1.354(1)	C1—H1A	1.091(1)
F7—C13	1.356(1)	C1—H1B	1.106(1)
F1—C9	1.356(1)	C10—H10	1.094(1)
F3—C10	1.347(1)	C11—H11A	1.095(1)
C4—H4	1.089(1)	C11—H11B	1.098(1)
C5—H5	1.090(1)	C13—H13	1.092(1)
C8—H8B	1.095(1)		
**Bond angle (°)**			
**Bond**	**Bond angle (°)**	**Bond**	**Bond angle (°)**
H3—C3—C2	120.1(1)	H8A—C8—O1	107.0(1)
H3—C3—C4	121.2(1)	H8B—C8—C9	108.8(1)
H7A—C7—H7B	108.4(1)	H8A—C8—C9	108.4(1)
H7A—C7—O2	110.3(1)	H1A—C1—C2	109.7(1)
H7B—C7—O2	109.6(1)	H1B—C1—C2	109.8(1)
H7A—C7—C6	110.2(1)	H10—C10—F4	109.9(1)
H7B—C7—C6	109.6(1)	H10—C10—F3	109.0(1)
H1A—C1—H1B	108.6(1)	H10—C10—C9	111.9(1)
H1A—C1—O1	111.4(1)	H11A—C11—H11B	109.8(1)
H1B—C1—O1	109.2(1)	H11A—C11—O2	109.6(1)
H4—C4—C5	119.8(1)	H11B—C11—O2	110.8(1)
H4—C4—C3	120.8(1)	H11A—C11—C12	109.9(1)
H5—C5—C4	121.4(1)	H11B—C11—C12	108.6(1)
H5—C5—C6	120.0(1)	H13—C13—F8	110.2(1)
H8B—C8—H8A	109.6(1)	H13—C13—F7	109.6(1)
H8B—C8—O1	111.2(1)	H13—C13—C12	112.5(1)

Table S5. Selected bond lengths (Å) and bond angles (°) for libration corrected monomeric **4FH-$ZnCl_2$(I)** complex.

**Bond length (Å)**			
**Bond**	**Bond length (Å)**	**Bond**	**Bond length (Å)**
F1—C9	1.357(1)	C4—H4	1.090(1)
F2—C9	1.355(1)	C5—H5	1.090(1)
F3—C10	1.348(1)	C7—H7A	1.094(1)
F4—C10	1.347(1)	C7—H7B	1.096(1)
F5—C12	1.364(1)	C8—H8A	1.099(1)
F6—C12	1.354(1)	C8—H8B	1.095(2)
F7—C13	1.358(1)	C10—H10	1.095(2)
F8—C13	1.352(1)	C11—H11A	1.095(2)
C1—H1A	1.092(1)	C11—H11B	1.098(1)
C1—H1B	1.108(2)	C13—H13	1.092(2)
C3—H3	1.085(1)		

**Bond angle (°)**			
**Bond**	**Bond angle (°)**	**Bond**	**Bond angle (°)**
O1—C1—H1A	111.4(1)	O1—C8—H8A	107.0(1)
O1—C1—H1B	109.2(2)	O1—C8—H8B	111.3(1)
C2—C1—H1A	109.8(1)	C9—C8—H8A	108.4(1)
C2—C1—H1B	109.8(1)	C9—C8—H8B	108.8(1)
H1A—C1—H1B	108.6(1)	H8A—C8—H8B	109.6(1)
C2—C3—H3	120.1(1)	F3—C10—H10	109.0(1)
C4—C3—H3	121.2(1)	F4—C10—H10	109.9(1)
C3—C4—H4	120.9(1)	C9—C10—H10	111.9(1)
C5—C4—H4	119.8(1)	O2—C11—H11A	109.6(1)
C4—C5—H5	121.4(1)	O2—C11—H11B	110.8(1)
C6—C5—H5	120.0(1)	C12—C11—H11A	109.9(1)
O2—C7—H7A	110.3(1)	C12—C11—H11B	108.6(1)
O2—C7—H7B	109.6(1)	H11A—C11—H11B	109.8(1)
C6—C7—H7A	110.1(1)	F7—C13—H13	109.6(1)
C6—C7—H7B	109.6(1)	F8—C13—H13	110.2(1)
H7A—C7—H7B	108.4(1)	C12—C13—H13	112.5(1)

Table S6. Crystallographic data and refinement parameters for **4FCl-ZnI$_2$(II)** complex.

	**X-ray diffraction CCDC ID: 2351847**	**Neutron diffraction CCDC ID: 2352174**
Crystal data		
Chemical formula	$C_{13}H_{11}Cl_2F_8I_2NO_2Zn$	$C_{13}H_{11}Cl_2F_8I_2NO_2Zn$
M_r	755.30	755.30
Crystal system, space group	Monoclinic, $C2/c$	Monoclinic, $C2/c$
Temperature (K)	100	40
a, b, c (Å)	22.8928 (3), 13.9616 (2), 17.3746 (2)	22.9805 (1), 13.9724 (1), 17.4000 (1)
α, β, γ (°)	129.863 (1)	90, 129.9252(3), 90
V (Å^3)	4262.57 (11)	4284.58 (5)
Z	8	8
Radiation type	Cu *K*a	Neutrons, l = 1.000 Å
m (mm^{-1})	27.42	0.21
Crystal size (mm)	0.21 × 0.17 × 0.09	2.50 × 2.50 × 1.0
Data collection		
Diffractometer	XtaLAB Synergy R, DW system, HyPix-Arc 150	Single-crystal diffractometer SENJU at the BL18 in the MLF of the J-PARC
Absorption correction	Multi-scan[a]	For a sphere[b]
T_{min}, T_{max}	0.574, 1.000	0.704, 0.748
No. of measured, independent and observed [I > 2s(I)] reflections	16390, 4107, 3916	73070, 13313, 9208
R_{int}	0.039	0.214
(sin q/l)$_{max}$ (Å^{-1})	0.622	0.995
Refinement		
$R[F^2 > 2s(F^2)]$, $wR(F^2)$, S	0.028, 0.071, 1.06	0.072, 0.185, 1.03
No. of reflections	4107	13313
No. of parameters	263	362
H-atom treatment	H-atom parameters constrained	All H-atom parameters refined
$\Delta\rho_{max}$, $\Delta\rho_{min}$	1.16, -0.76(e Å^{-3})	1.91, -2.76(fm Å^{-3})

(a) Bruker SADABS, 1996. (b) Tibballs, J. E., Acta Cryst. A38, 161-163, 1982.

Table S7. Weak interactions of tetrel bond and improper HB in **4FCl-ZnI₂(II)** complex [Å and °].

Item no	**a) Tetrel bond (O−C···F−C) in 4FCl-ZnI$_2$(II)**				
	**H/C—C···F—C**	**H/C—C**	**C···F**	**H/C···F or C···C**	**∠CFC**
1	C2−C1···F2−C9	C2−C1= 1.502(1)<br>F2−C9 = 1.352(3)	C1···F2=2.956(1)	C2···F2= 4.350(1)<br>C1···C9= 3.098(1)	∠C2C1F2= 153.2(1)<br>∠C9F2C1= 83.1(1)
	**b) Improper hydrogen bonds (C−H···*A*)**				
	D*—H···*A	***D*—H**	**H···*A***	***D*···*A***	**∠*D*H*A***
2	C1—H1A···F2	1.095(4)	2.351(3)	2.956(1)	113.0(2)

Note: neutron structure of **4FCl-ZnI$_2$** was colloected at 40K, so the libration correction was not necessary.

Table S8. Selected bond lengths (Å) and bond angles (°) for monomeric **4FCl-ZnI$_2$(II)** complex (at 40K).

**Bond length (Å)**			
**Bond**	**Bond length (Å)**	**Bond**	**Bond length (Å)**
F1—C9	1.348(2)	C1—H1B	1.108(2)
F2—C9	1.352(2)	C3—H3	1.082(2)
F3—C10	1.345(2)	C4—H4	1.094(2)
F4—C10	1.333(2)	C5—H5	1.091(2)
F5—C12	1.342(2)	C7—H7A	1.098(3)
F6—C12	1.350(2)	C7—H7B	1.101(3)
F7—C13	1.337(2)	C8—H8A	1.098(3)
F8—C13	1.329(2)	C8—H8B	1.100(3)
C1—H1A	1.095(4)	C11—H11A	1.093(3)
		C11—H11B	1.101(3)

**Bond angle (°)**			
**Bond**	**Bond angle (°)**	**Bond**	**Bond angle (°)**
H1A—C1—H1B	108.5(2)	H7B—C7—O2	109.4(2)
H1A—C1—O1	111.5(2)	H7A—C7—C6	110.3(2)
H1B—C1—O1	109.4(2)	H7B—C7—C6	108.8(2)
H1A—C1—C2	109.9(2)	H8A—C8—H8B	108.8(3)
H1B—C1—C2	110.7(2)	H8A—C8—O1	107.2(2)
H3—C3—C4	121.6(2)	H8B—C8—O1	111.7(2)
H3—C3—C2	119.7(2)	H8A—C8—C9	107.8(2)
H4—C4—C5	120.2(2)	H8B—C8—C9	107.3(2)
H4—C4—C3	120.5(2)	H11A—C11—H11B	110.1(3)
H5—C5—C4	120.5(2)	H11A—C11—O2	110.6(2)
H5—C5—C6	120.7(2)	H11B—C11—O2	110.2(2)
H7A—C7—H7B	108.8(3)	H11A—C11—C12	109.9(2)
H7A—C7—O2	110.5(2)	H11B—C11—C12	107.5(2)

Note: neutron structure of **4FCl-ZnI$_2$** was colloected at 40K, so the libration correction was not necessary.

Table S9. Crystallographic data and refinement parameters for **2FCl-ZnI₂(III)** complex.

	X-ray diffraction CCDC ID: 2351848	Neutron diffraction CCDC ID: 2352218
Crystal data		
Chemical formula	$C_{11}H_{11}Cl_2F_4I_2NO_2Zn$	$C_{11}H_{11}Cl_2F_4I_2NO_2Zn$
M_r	655.28	655.28
Crystal system, space group	Monoclinic, $C2/c$	Monoclinic, $C2/c$
Temperature (K)	100	100
a, b, c (Å)	8.2500 (1), 17.5030 (3), 12.8686 (2)	8.2682 (3), 17.5189 (6), 12.9122 (5)
α, β, γ (°)	90, 91.650 (2), 90	90, 91.699 (3), 90
V (Å^3)	1857.45 (5)	1869.51 (12)
Z	4	4
Radiation type	Cu Ka	Neutrons, l = 0.700 Å
m (mm^{-1})	31.00	0.0674 + 0.07163λ
Crystal size (mm)	0.29 × 0.18 × 0.09	2.25 × 1.55 × 1.50
Data collection		
Diffractometer	XtaLAB Synergy R, DW system, HyPix-Arc 150	TOPAZ
Absorption correction	Multi-scan[a]	Gaussian[b]
T_{min}, T_{max}	0.574, 1.000	0.807, 0.927
No. of measured, independent and observed [I > 2s(I)] reflections	4868, 1769, 1701	24644, 4535, 3820
R_{int}	0.029	0.095
(sin q/l)$_{max}$ (Å^{-1})	0.627	1.399
Refinement		
$R[F^2 > 2s(F^2)]$, $wR(F^2)$, S	0.022, 0.053, 1.06	0.034, 0.079, 1.19
No. of reflections	3164	4535
No. of parameters	245	157
H-atom treatment	H-atom parameters constrained	All H-atom parameters refined
$\Delta\rho_{max}$, $\Delta\rho_{min}$	0.30, -0.28(e Å^{-3})	0.71, -0.68 (fm Å^{-3})

(a) Bruker SADABS, 1996. (b) P. Coppens, L. Leiserowitz, D. Rabinovich, Acta Cryst. 18, 1035-1038 (1965).

Table S10. Weak interactions of tetrel bond and improper HB in **2FCl-ZnI$_2$(III)** complex [Å and °].(without libration correction).

Item no	a) Tetrel bond (O–C···F–C) in 2FCl-ZnI$_2$(III)				
	**H/C—C···F—C**	**H/C—C**	**C···F**	**H/C···F or C···C**	**∠CFC**
1	C2–C1···F1–C6	C2–C1= 1.506(1)<br>F1–C6 = 1.347(1)	C1···F1=3.127(1)	C2···F1= 4.432(1)<br>C1···C6= 3.235(1)	∠C2C1F1= 143.8(0)<br>∠C6F1C1= 86.3(0)
	**b) Improper hydrogen bonds (C–H···*A*)**				
	D*—H···*A	***D*—H**	**H···*A***	***D*···*A***	**∠*D*H*A***
2	C1—H1B···F1	1.093(1)	2.534(2)	2.235(1)	112.9(1)

Table S11. Weak interactions of tetrel bond and improper HB in libration-corrected **2FCl-ZnI$_2$(III)** complex [Å and °].

Item no	a) Tetrel bond (O–C···F–C) in 2FCl-ZnI$_2$(II)				
	**H/C—C···F—C**	**H/C—C**	**C···F**	**H/C···F or C···C**	**∠CFC**
1	C2–C1···F1–C6	C2–C1= 1.508(1)<br>F1–C6 = 1.348(1)	C1···F1=3.129(1)	C2···F1= 4.435(1)<br>C1···C6= 3.237(1)	∠C2C1F1= 143.8(0)<br>∠C6F1C1= 82.3(0)
	**b) Improper hydrogen bonds (C–H···*A*)**				
	D*—H···*A	***D*—H**	**H···*A***	***D*···*A***	**∠*D*H*A***
2	C1—H1B···F1	1.093(1)	2.536(2)	3.129(1)	112.9(1)

Table S12. Selected bond lengths (Å) and bond angles (°) for monomeric **2FCl-ZnI$_2$(III)** complex (without libration correction).

**Bond length (Å)**			
**Bond**	**Bond length (Å)**	**Bond**	**Bond length (Å)**
F1—C6	1.347(1)	C5—H5B	1.091(1)
F2—C6	1.332(1)	C5—H5A	1.099(1)
C3—H3	1.086(1)	C1—H1B	1.093(1)
C4—H4	1.087(2)	C1—H1A	1.102(1)
**Bond angle (°)**			
**Bond**	**Bond angle (°)**	**Bond**	**Bond angle (°)**
H3—C3—C2	120.3(1)	H5B—C5—C6	108.1(1)
H3—C3—C4	121.1(1)	H5A—C5—C6	109.0(1)
H4—C4—C3	120.2(0)	H1B—C1—H1A	109.1(1)
H4—C4—C3^{i}	120.2(0)	H1B—C1—O1	110.8(1)
H5B—C5—H5A	109.0(1)	H1A—C1—O1	109.2(1)
H5B—C5—O1	107.7(1)	H1B—C1—C2	109.8(1)
H5A—C5—O1	112.5(1)	H1A—C1—C2	109.5(1)

Symmetry code: (i) -*x*+1, *y*, -*z*+1/2.

Table S13. Selected bond lengths (Å) and bond angles (°) for libration corrected monomeric **2FCl-ZnI$_2$(III)** complex.

**Bond length (Å)**			
**Bond**	**Bond length (Å)**	**Bond**	**Bond length (Å)**
F1—C6	1.348(1)	C3—H3	1.086(1)
F2—C6	1.333(1)	C4—H4	1.088(2)
C1—H1A	1.103(1)	C5—H5A	1.100(1)
C1—H1B	1.093(1)	C5—H5B	1.092(1)
**Bond angle (°)**			
**Bond**	**Bond angle (°)**	**Bond**	**Bond angle (°)**
O1—C1—H1A	109.2(1)	C3—C4—H4	120.2(0)
O1—C1—H1B	110.8(1)	C3^{i}—C4—H4	120.2(0)
C2—C1—H1A	109.5(1)	O1—C5—H5A	112.5(1)
C2—C1—H1B	109.8(1)	O1—C5—H5B	107.7(1)
H1A—C1—H1B	109.1(1)	C6—C5—H5A	109.0(1)
C2—C3—H3	120.3(1)	C6—C5—H5B	108.1(1)
C4—C3—H3	121.1(1)	H5A—C5—H5B	109.0(1)

Symmetry code: (i) -*x*+1, *y*, -*z*+1/2

Table S14. Experimental data of the wavenumber vs. neutron C–H bond length

**Item**	**C–H vibration (cm^{-1})**	**C–H Bond length (Å)**	**Comments**
1	2688	1.108 (2)	Complex **I**
2	3020	1.092 (1)	
3	2702	1.108(2)	Complex **II**
4	2949	1.095 (4)	
5	2817	1.103(2)	Complex **III**
6	2962	1.093(1)	

Table S15. Deuterated experimental data of the wavenumber vs. neutron C–H bond length

**Item**	**C–D vibration (cm^{-1})**	**C–H Bond length (Å)**	**Comments**
1	1972	1.108 (2)	Complex **I**
2	2216	1.092 (1)	
3	1989	1.108(2)	Complex **II**
4	2165	1.095 (4)	
5	2070	1.103(2)	Complex **III**
6	2177	1.093(1)	

Table S16. Calculated C–H bond length and its force constant using by using PyMOL software.

**Item**	**Calc. C–H Bond length (Å)**	**Calc. force constant (mdyn/Å)**	**Comments**
1	1.1068	5.083	Complex **I**
2	1.0989	5.363	
3	1.1059	5.117	Complex **II**
4	1.0986	5.374	
5	1.1066	5.089	Complex **III**
6	1.0991	5.359	

V. (A) Atomic coordinates of complexes (I-III) using MP2 level of theory

Table S16. Atomic coordinates of optimized **4FH-$ZnCl_2$(I)** monomer using MP2 theoretical calculations (H-C-H).

Atom	X	Y	Z
Zn	-0.06024	-0.304039	0.046664
Cl	0.17514	-1.25299	2.117144
Cl	-0.249878	-1.366677	-1.977095
F	-4.500601	-0.176172	1.672965
F	-5.143052	0.785103	-0.312822
F	6.738303	-1.365324	-0.603712
O	2.135304	0.302726	-0.011834
O	-2.240572	0.374304	0.011081
F	-5.158507	-2.753018	0.611936
F	5.119426	0.458305	0.592501
F	-5.834133	-1.730878	-1.347183
F	4.418135	0.101824	-1.56908
F	5.313326	-2.502623	0.822221
N	-0.014775	1.78493	0.015354
C	-1.134743	2.477104	-0.375842
C	1.127939	2.437723	0.408819
C	0.027075	4.592787	0.034429
H	0.042866	5.685257	0.043763
C	-1.14927	3.897303	-0.367281
H	-2.049589	4.438774	-0.66756
C	3.060007	-0.811196	0.273614
H	2.623664	-1.672074	-0.254262
H	3.11051	-1.019731	1.353829
C	4.47226	-0.510184	-0.266869
C	1.183525	3.857246	0.421019
H	2.101041	4.367381	0.724091
C	-4.576092	-0.427623	0.261882
C	-2.309548	1.61347	-0.832677
H	-3.264513	2.131349	-0.667396
H	-2.203045	1.316212	-1.893458
C	2.288391	1.534279	0.826923
H	3.253771	2.018075	0.618626
H	2.218532	1.243708	1.892145
C	5.382388	-1.757572	-0.409309
H	5.083258	-2.413593	-1.235203
C	-3.188272	-0.707193	-0.337996
H	-2.772619	-1.617856	0.114311
H	-3.261821	-0.828781	-1.430641
C	-5.652963	-1.524981	0.062923
H	-6.619013	-1.285902	0.520302

Table S17. Atomic coordinates of optimized **4FH-$ZnCl_2$(I)** monomer using MP2 theoretical calculations (D-C-H).

Atom	X	Y	Z
Zn	-0.06024	-0.304039	0.046664
Cl	0.17514	-1.25299	2.117144
Cl	-0.249878	-1.366677	-1.977095
F	-4.500601	-0.176172	1.672965
F	-5.143052	0.785103	-0.312822
F	6.738303	-1.365324	-0.603712
O	2.135304	0.302726	-0.011834
O	-2.240572	0.374304	0.011081
F	-5.158507	-2.753018	0.611936
F	5.119426	0.458305	0.592501
F	-5.834133	-1.730878	-1.347183
F	4.418135	0.101824	-1.56908
F	5.313326	-2.502623	0.822221
N	-0.014775	1.78493	0.015354
C	-1.134743	2.477104	-0.375842
C	1.127939	2.437723	0.408819
C	0.027075	4.592787	0.034429
H	0.042866	5.685257	0.043763
C	-1.14927	3.897303	-0.367281
H	-2.049589	4.438774	-0.66756
C	3.060007	-0.811196	0.273614
H	2.623664	-1.672074	-0.254262
H	3.11051	-1.019731	1.353829
C	4.47226	-0.510184	-0.266869
C	1.183525	3.857246	0.421019
H	2.101041	4.367381	0.724091
C	-4.576092	-0.427623	0.261882
C	-2.309548	1.61347	-0.832677
H	-3.264513	2.131349	-0.667396
H	-2.203045	1.316212	-1.893458
C	2.288391	1.534279	0.826923
H	3.253771	2.018075	0.618626
H	2.218532	1.243708	1.892145
C	5.382388	-1.757572	-0.409309
H	5.083258	-2.413593	-1.235203
C	-3.188272	-0.707193	-0.337996
H	-2.772619	-1.617856	0.114311
H	-3.261821	-0.828781	-1.430641
C	-5.652963	-1.524981	0.062923
H	-6.619013	-1.285902	0.520302

Table S18. Atomic coordinates of optimized **4FH-ZnCl$_2$(I)** monomer using MP2 theoretical calculations (H-C-D).

Atom	X	Y	Z
Zn	-0.06024	-0.304039	0.046664
Cl	0.17514	-1.25299	2.117144
Cl	-0.249878	-1.366677	-1.977095
F	-4.500601	-0.176172	1.672965
F	-5.143052	0.785103	-0.312822
F	6.738303	-1.365324	-0.603712
O	2.135304	0.302726	-0.011834
O	-2.240572	0.374304	0.011081
F	-5.158507	-2.753018	0.611936
F	5.119426	0.458305	0.592501
F	-5.834133	-1.730878	-1.347183
F	4.418135	0.101824	-1.56908
F	5.313326	-2.502623	0.822221
N	-0.014775	1.78493	0.015354
C	-1.134743	2.477104	-0.375842
C	1.127939	2.437723	0.408819
C	0.027075	4.592787	0.034429
H	0.042866	5.685257	0.043763
C	-1.14927	3.897303	-0.367281
H	-2.049589	4.438774	-0.66756
C	3.060007	-0.811196	0.273614
H	2.623664	-1.672074	-0.254262
H	3.11051	-1.019731	1.353829
C	4.47226	-0.510184	-0.266869
C	1.183525	3.857246	0.421019
H	2.101041	4.367381	0.724091
C	-4.576092	-0.427623	0.261882
C	-2.309548	1.61347	-0.832677
H	-3.264513	2.131349	-0.667396
H	-2.203045	1.316212	-1.893458
C	2.288391	1.534279	0.826923
H	3.253771	2.018075	0.618626
H	2.218532	1.243708	1.892145
C	5.382388	-1.757572	-0.409309
H	5.083258	-2.413593	-1.235203
C	-3.188272	-0.707193	-0.337996
H	-2.772619	-1.617856	0.114311
H	-3.261821	-0.828781	-1.430641
C	-5.652963	-1.524981	0.062923
H	-6.619013	-1.285902	0.520302

Table S19. Atomic coordinates of optimized **4FCl-ZnI$_2$(II)** monomer using MP2 theoretical calculations (H-C-H).

**Atom**	**X**	**Y**	**Z**
I	-0.1578	-1.05297	2.3422
I	0.34902	-1.51417	-2.34814
Zn	-0.0504	-0.17096	-0.12973
Cl	7.27278	0.02831	-0.15869
Cl	-6.81763	0.0028	1.09989
F	4.78422	1.69706	0.6776
F	4.43044	0.59391	-1.3024
F	-5.28389	-2.01209	-0.55094
F	-5.18348	0.00613	-1.64903
F	-4.32268	-0.56293	1.85198
F	-4.58953	1.37828	0.65804
F	5.70038	-0.70388	1.8673
F	5.36628	-1.83343	-0.09381
O	-2.29447	0.18349	-0.53215
O	2.01553	0.80376	0.13461
N	-0.31605	1.89342	-0.35571
C	0.62396	2.75631	0.15484
C	-1.43506	2.3655	-1.00056
C	4.4566	0.40304	0.11121
C	-1.6588	3.76016	-1.14486
H	-2.56083	4.12066	-1.645
C	-2.37315	1.29189	-1.54217
H	-3.40065	1.67388	-1.6148
H	-2.02758	0.89754	-2.5159
C	-4.61572	-0.74226	-0.54801
C	-3.11685	-1.01282	-0.79923
H	-2.76061	-1.77246	-0.09039
H	-2.99883	-1.36262	-1.83845
C	5.65147	-0.54647	0.44684
C	-0.6912	4.66821	-0.62839
H	-0.83757	5.7459	-0.73245
C	-5.01625	0.01838	0.76047
C	3.08818	-0.07407	0.64181
H	2.86405	-1.07148	0.23904
H	3.09703	-0.09799	1.74349
C	1.8022	2.09657	0.86663
H	2.7005	2.72559	0.79819
H	1.56142	1.86343	1.92122
C	0.46824	4.1623	0.02456
H	1.22969	4.83654	0.42406

Table S20. Atomic coordinates of optimized **4FCl-ZnI$_2$(II)** monomer using MP2 theoretical calculations (D-C-H).

Atom	X	Y	Z
I	-0.1578	-1.05297	2.3422
I	0.34902	-1.51417	-2.34814
Zn	-0.0504	-0.17096	-0.12973
Cl	7.27278	0.02831	-0.15869
Cl	-6.81763	0.0028	1.09989
F	4.78422	1.69706	0.6776
F	4.43044	0.59391	-1.3024
F	-5.28389	-2.01209	-0.55094
F	-5.18348	0.00613	-1.64903
F	-4.32268	-0.56293	1.85198
F	-4.58953	1.37828	0.65804
F	5.70038	-0.70388	1.8673
F	5.36628	-1.83343	-0.09381
O	-2.29447	0.18349	-0.53215
O	2.01553	0.80376	0.13461
N	-0.31605	1.89342	-0.35571
C	0.62396	2.75631	0.15484
C	-1.43506	2.3655	-1.00056
C	4.4566	0.40304	0.11121
C	-1.6588	3.76016	-1.14486
H	-2.56083	4.12066	-1.645
C	-2.37315	1.29189	-1.54217
H	-3.40065	1.67388	-1.6148
H	-2.02758	0.89754	-2.5159
C	-4.61572	-0.74226	-0.54801
C	-3.11685	-1.01282	-0.79923
H	-2.76061	-1.77246	-0.09039
H	-2.99883	-1.36262	-1.83845
C	5.65147	-0.54647	0.44684
C	-0.6912	4.66821	-0.62839
H	-0.83757	5.7459	-0.73245
C	-5.01625	0.01838	0.76047
C	3.08818	-0.07407	0.64181
H	2.86405	-1.07148	0.23904
H	3.09703	-0.09799	1.74349
C	1.8022	2.09657	0.86663
H	2.7005	2.72559	0.79819
H	1.56142	1.86343	1.92122
C	0.46824	4.1623	0.02456
H	1.22969	4.83654	0.42406

Table S21. Atomic coordinates of optimized **4FCl-ZnI$_2$(II)** monomer using MP2 theoretical calculations (H-C-D).

Atom	X	Y	Z
I	-0.1578	-1.05297	2.3422
I	0.34902	-1.51417	-2.34814
Zn	-0.0504	-0.17096	-0.12973
Cl	7.27278	0.02831	-0.15869
Cl	-6.81763	0.0028	1.09989
F	4.78422	1.69706	0.6776
F	4.43044	0.59391	-1.3024
F	-5.28389	-2.01209	-0.55094
F	-5.18348	0.00613	-1.64903
F	-4.32268	-0.56293	1.85198
F	-4.58953	1.37828	0.65804
F	5.70038	-0.70388	1.8673
F	5.36628	-1.83343	-0.09381
O	-2.29447	0.18349	-0.53215
O	2.01553	0.80376	0.13461
N	-0.31605	1.89342	-0.35571
C	0.62396	2.75631	0.15484
C	-1.43506	2.3655	-1.00056
C	4.4566	0.40304	0.11121
C	-1.6588	3.76016	-1.14486
H	-2.56083	4.12066	-1.645
C	-2.37315	1.29189	-1.54217
H	-3.40065	1.67388	-1.6148
H	-2.02758	0.89754	-2.5159
C	-4.61572	-0.74226	-0.54801
C	-3.11685	-1.01282	-0.79923
H	-2.76061	-1.77246	-0.09039
H	-2.99883	-1.36262	-1.83845
C	5.65147	-0.54647	0.44684
C	-0.6912	4.66821	-0.62839
H	-0.83757	5.7459	-0.73245
C	-5.01625	0.01838	0.76047
C	3.08818	-0.07407	0.64181
H	2.86405	-1.07148	0.23904
H	3.09703	-0.09799	1.74349
C	1.8022	2.09657	0.86663
H	2.7005	2.72559	0.79819
H	1.56142	1.86343	1.92122
C	0.46824	4.1623	0.02456
H	1.22969	4.83654	0.42406

Table S22. Atomic coordinates of optimized **2FCl-ZnI$_2$**(**III**) monomer using MP2 theoretical calculations (H-C-H).

Atom	X	Y	Z
I	0.00298	1.23535	-2.36961
Zn	4E-6	0.09862	4.3E-5
Cl	-5.7489	1.54646	0.05534
F	-5.11299	-1.02267	0.42827
F	-4.47458	-0.14182	-1.57499
O	-2.1961	-0.56906	0.11878
N	1.86E-4	-1.99533	-4.71E-4
C	-1.13495	-4.09214	0.4729
H	-2.0217	-4.61706	0.83634
C	-1.10051	-2.67222	0.46882
C	-4.55372	0.15692	-0.1896
C	4.36E-4	-4.8087	-9.75E-4
H	5.31E-4	-5.90126	-0.00117
C	-3.18043	0.4903	0.42011
H	-3.28574	0.64013	1.50711
H	-2.78668	1.3989	-0.05534
C	-2.23731	-1.79375	0.98483
H	-2.0682	-1.48413	2.03373
H	-3.20514	-2.30329	0.87698
Cl	5.74918	1.54686	-0.0548
F	5.11333	-1.0221	-0.42898
F	4.47507	-0.1423	1.57478
O	2.19647	-0.56878	-0.11902
C	1.13569	-4.09176	-0.4746
H	2.02253	-4.61639	-0.83825
C	1.10101	-2.67185	-0.47002
C	4.55407	0.15715	0.18953
C	3.18071	0.49079	-0.41988
H	3.28592	0.6412	-1.50682
H	2.78697	1.39912	0.05607
C	2.23764	-1.793	-0.98576
H	2.06833	-1.48282	-2.03446
H	3.20553	-2.3025	-0.87833
I	-0.00364	1.23414	2.37027

Table S23. Atomic coordinates of optimized **2FCl-ZnI$_2$(III)** monomer using MP2 theoretical calculations (D-C-H).

Atom	X	Y	Z
I	0.00298	1.23535	-2.36961
Zn	4E-6	0.09862	4.3E-5
Cl	-5.7489	1.54646	0.05534
F	-5.11299	-1.02267	0.42827
F	-4.47458	-0.14182	-1.57499
O	-2.1961	-0.56906	0.11878
N	1.86E-4	-1.99533	-4.71E-4
C	-1.13495	-4.09214	0.4729
H	-2.0217	-4.61706	0.83634
C	-1.10051	-2.67222	0.46882
C	-4.55372	0.15692	-0.1896
C	4.36E-4	-4.8087	-9.75E-4
H	5.31E-4	-5.90126	-0.00117
C	-3.18043	0.4903	0.42011
H	-3.28574	0.64013	1.50711
H	-2.78668	1.3989	-0.05534
C	-2.23731	-1.79375	0.98483
H	-2.0682	-1.48413	2.03373
H	-3.20514	-2.30329	0.87698
Cl	5.74918	1.54686	-0.0548
F	5.11333	-1.0221	-0.42898
F	4.47507	-0.1423	1.57478
O	2.19647	-0.56878	-0.11902
C	1.13569	-4.09176	-0.4746
H	2.02253	-4.61639	-0.83825
C	1.10101	-2.67185	-0.47002
C	4.55407	0.15715	0.18953
C	3.18071	0.49079	-0.41988
H	3.28592	0.6412	-1.50682
H	2.78697	1.39912	0.05607
C	2.23764	-1.793	-0.98576
H	2.06833	-1.48282	-2.03446
H	3.20553	-2.3025	-0.87833
I	-0.00364	1.23414	2.37027

Table S24. Atomic coordinates of optimized **2FCl-ZnI$_2$(III)** monomer using MP2 theoretical calculations (H-C-D).

Atom	X	Y	Z
I	0.00298	1.23535	-2.36961
Zn	4E-6	0.09862	4.3E-5
Cl	-5.7489	1.54646	0.05534
F	-5.11299	-1.02267	0.42827
F	-4.47458	-0.14182	-1.57499
O	-2.1961	-0.56906	0.11878
N	1.86E-4	-1.99533	-4.71E-4
C	-1.13495	-4.09214	0.4729
H	-2.0217	-4.61706	0.83634
C	-1.10051	-2.67222	0.46882
C	-4.55372	0.15692	-0.1896
C	4.36E-4	-4.8087	-9.75E-4
H	5.31E-4	-5.90126	-0.00117
C	-3.18043	0.4903	0.42011
H	-3.28574	0.64013	1.50711
H	-2.78668	1.3989	-0.05534
C	-2.23731	-1.79375	0.98483
H	-2.0682	-1.48413	2.03373
H	-3.20514	-2.30329	0.87698
Cl	5.74918	1.54686	-0.0548
F	5.11333	-1.0221	-0.42898
F	4.47507	-0.1423	1.57478
O	2.19647	-0.56878	-0.11902
C	1.13569	-4.09176	-0.4746
H	2.02253	-4.61639	-0.83825
C	1.10101	-2.67185	-0.47002
C	4.55407	0.15715	0.18953
C	3.18071	0.49079	-0.41988
H	3.28592	0.6412	-1.50682
H	2.78697	1.39912	0.05607
C	2.23764	-1.793	-0.98576
H	2.06833	-1.48282	-2.03446
H	3.20553	-2.3025	-0.87833
I	-0.00364	1.23414	2.37027

(B) Atomic coordinates of complexes (I-III) using DFT (ωB97X-D) level of theory

Table S25. Atomic coordinates of optimized **4FH-ZnCl$_2$(I)** monomer using DFT theoretical calculations (H-C-H).

Atom	X	Y	Z
Zn	0.05423	-0.402149	-0.000931
Cl	-0.22376	-1.311559	-1.979281
Cl	0.32	-1.252459	2.007119
F	4.76237	0.237221	-1.382341
F	5.16806	0.639861	0.732659
F	-6.73101	-1.313669	0.487309
O	-2.22624	0.312541	0.167559
O	2.32041	0.361131	-0.172461
F	5.25716	-2.429119	-0.985281
F	-5.1088	0.462151	-0.654521
F	5.58171	-2.053689	1.157009
F	-4.60316	0.182321	1.460299
F	-5.33953	-2.348429	-0.866371
N	0.02542	1.682331	-0.035161
C	1.14208	2.364941	0.238429
C	-1.10711	2.325041	-0.339161
C	-0.00629	4.436001	-0.102101
H	-0.01874	5.525061	-0.129011
C	1.16707	3.754881	0.207619
H	2.09078	4.290211	0.419459
C	-3.11546	-0.736339	-0.147191
H	-2.77285	-1.598259	0.438209
H	-3.07028	-0.996519	-1.213861
C	-4.55584	-0.415649	0.237559
C	-1.16377	3.714041	-0.376281
H	-2.09978	4.217021	-0.612031
C	4.67152	-0.302719	-0.137921
C	2.35396	1.546741	0.607419
H	3.26642	2.123161	0.412219
H	2.31323	1.290051	1.679849
C	-2.30201	1.462811	-0.660521
H	-3.22736	2.022451	-0.477091
H	-2.26679	1.162331	-1.721621
C	-5.43919	-1.670309	0.309609
H	-5.13188	-2.330059	1.132479
C	3.22939	-0.652759	0.202839
H	2.9419	-1.539929	-0.371891
H	3.15253	-0.882759	1.274619
C	5.63863	-1.494139	-0.080071
H	6.66948	-1.180699	-0.291541

Note: The calculations of **4FH-ZnCl$_2$(I)** have been done by using ωB97X-D/aug-cc-pVDZ level of theory.

Table S26. Atomic coordinates of optimized **4FCl-ZnI$_2$(II)** monomer using MP2 theoretical calculations (H-C-H).

Atom	X	Y	Z
I	0.12132	-1.21521	-2.11821
I	-0.22935	-1.22436	2.49673
Zn	0.046279	-0.19262	0.19925
Cl	-7.258591	-0.082472	-0.18034
Cl	6.789289	-0.201458	-1.17302
F	-4.842591	1.387179	-1.14277
F	-4.661721	0.979959	1.00088
F	5.28261	-1.745528	0.83241
F	5.207009	0.369392	1.40672
F	4.41806	-0.952849	-1.74888
F	4.611739	1.117261	-1.10593
F	-5.47589	-1.280841	-1.56343
F	-5.38225	-1.634361	0.57992
O	2.384429	0.327911	0.40868
O	-2.146801	0.80003	0.00379
N	0.257929	1.89066	0.20975
C	-0.728181	2.66402	-0.25937
C	1.384649	2.442401	0.67624
C	-4.543041	0.420329	-0.22357
C	1.570878	3.819951	0.68235
H	2.502498	4.243121	1.0532
C	2.427089	1.496251	1.21387
H	3.414559	1.970611	1.17496
H	2.191189	1.235131	2.25999
C	4.675329	-0.563778	0.56468
C	3.1827	-0.753639	0.83086
H	2.84299	-1.626449	0.26313
H	3.06067	-0.955099	1.90566
C	-5.60957	-0.698621	-0.35657
C	0.549928	4.634	0.20227
H	0.665398	5.71722	0.19861
C	5.039809	-0.139458	-0.88482
C	-3.126261	-0.109021	-0.44529
H	-2.98792	-1.01629	0.15302
H	-2.992431	-0.35457	-1.50808
C	-1.947991	1.95739	-0.79336
H	-2.815121	2.62674	-0.74386
H	-1.778321	1.66325	-1.84364
C	-0.619672	4.05028	-0.27327
H	-1.441352	4.65823	-0.64722

Note: The calculations of **4FCl-ZnI$_2$(II)** have been done by using ωB97X-D/aug-cc-pVDZ level of theory.

Table S27. Atomic coordinates of optimized **2FCl-ZnI$_2$(III)** monomer using MP2 theoretical calculations (H-C-H).

Atom	X	Y	Z
I	-0.094251	1.208167	-2.310505
Zn	0.000027	0.175131	0.000133
Cl	5.710264	1.486675	0.08739
F	5.15107	-0.987572	0.402285
F	4.622541	-0.169213	-1.5341
O	2.300049	-0.590002	-0.054105
N	0.000007	-1.916986	-0.000719
C	1.146315	-3.973272	0.354612
H	2.062657	-4.492137	0.629731
C	1.106886	-2.583388	0.349061
C	4.626269	0.101645	-0.219139
C	-0.000059	-4.676083	-0.001554
H	-0.000086	-5.765465	-0.00188
C	3.225727	0.416525	0.293742
H	3.264667	0.577875	1.380256
H	2.883945	1.335345	-0.194998
C	2.296085	-1.753289	0.760049
H	2.196699	-1.464417	1.820911
H	3.217004	-2.33359	0.630024
Cl	-5.710287	1.486771	-0.086895
F	-5.151061	-0.987247	-0.403517
F	-4.622879	-0.170331	1.53357
O	-2.300122	-0.590063	0.053627
C	-1.146397	-3.973	-0.357311
H	-2.062758	-4.491656	-0.63276
C	-1.106902	-2.583125	-0.350924
C	-4.626361	0.101504	0.218809
C	-3.225722	0.416753	-0.29358
H	-3.264474	0.578958	-1.379975
H	-2.884007	1.335184	0.195938
C	-2.296025	-1.752695	-0.761462
H	-2.19643	-1.462954	-1.822068
H	-3.216976	-2.333092	-0.632088
I	0.094325	1.206035	2.311739

Note: The calculations of 2**FCl-ZnI$_2$(III)** have been done by using ωB97X-D/aug-cc-pVDZ level of theory.

VI. Synthesis of deuterated ligands, their meta complexes (I-III) and related compounds

Synthesis of deuterated alcohols 2,6-$(CD_2OD)_2$-py

To a stirring solution of the 2,6-pincer-dimethyl ester (0.877 g, 4.5 mmol) in 60 mL anhydrous ethanol under an atmosphere of N_2, $NaBD_4$ (1.0 g, 24.05 mmol) and $CaCl_2$ (3.0 g, 27 mmol) was added slowly in portions at at 0°C. The evolution of D_2 was allowed to cease before each further addition. The reaction mixture was then stirred at 0°C. for 6-7h. When the reaction is complete the solvent was removed under vacuum leaving a white powder. Then add 100 mL distilled water and add conc. HCl to neutralize the reaction in an ice bath environment. To the resultant mixture, add ethyl acetate and kept in sonication for 40 min. Then collect the clean organic layer using separatory funnel, repeat this procedure for three times and concentrated under reduced pressure to give the deuterated 2,6-$(CD_2OD)_2$-py as white solid.

Analytical data for 2,6-$(CD_2OD)_2$-py: ^1H NMR (400 MHz, DNSO-d_6, ppm): δ= 7.79 (1H, t, $^3J_{H,H}$= 7.8 Hz, H-4), 7.32 (2H, d, $^3J_{H,H}$= 7.8 Hz, $H_{3/5}$), 3.93 (2H, br., $CD_2O\underline{H}$; Yield: 77.7%; m.p. 118-120 °C.

Synthesis of deuterated 2,6-$(CD_2Br)_2$-py

Deuterated 2,6-pyridinedimethanol (6.91 mmol, 0.99 g) was dissolved in 5.3 mL dimethylformamide. Then phosphorus tribromide (16.25 mmol, 1.54 mL) was added drop by drop and stirred in an ice bath for 4 hours and left at room temperature overnight. After the reaction was completed, the reaction was quenched with large amount of water. The resulting solution was extracted three times with diethyl ether and the extractive was concentrated under vacuum to give the deuterated 2,6-$(CD_2Br)_2$-py as white solid.

Analytical data for deuterated 2,6-$(CD_2Br)_2$-py: ^1H NMR (400 MHz, $CDCl_3$, ppm): δ= 7.70 (1H, t, $^3J_{H,H}$= 7.7 Hz, H4), 7.36 (2H, d, $^3J_{H,H}$= 7.7 Hz, $H_{3/5}$). Yield: 40%; m.p. 108-110 °C.

Synthesis of deuterated 2,6-(R_f-CH_2OCD_2)$_2$-py ligands

[Note: R_f (short fluorinated chain) = HCF_2CF_2-, $ClCF_2CF_2$-, $ClCF_2$-]

For example, synthesis of deuterated 2,6-($HCF_2CF_2CH_2OCD_2$)$_2$-py ligand

A magnetic stirrer and vacuum system were equipped in a 250 mL double-necked round-bottom flask. The air and moisture were pumped out of the flask using the vacuum system. $HCF_2CF_2CH_2OH$ (2.5 mmol, 0.32 g) and CH_3ONa/CH_3OH (1.9 mmol, 0.35 g) were mixed in a two-necked flask and continuously stirred at 60 ^{0}C under nitrogen for 4 hours. The methanol was removed using a vacuum system to move the reaction to the product side. The obtained sodium fluorinated alkoxide was dissolved in dry THF, and then deuterated 2,6-py-(CD_2Br)$_2$ (0.7 mmol, 0.20 g) was added, and the mixture was continuously stirred for 4 hours under a nitrogen atmosphere at room temperature. After confirming that the ligand was formed by using GC/MS analysis, the reaction was quenched by adding water. The resulting solution was extracted with dichloromethane and water. The organic phase was then dried over Na_2SO_4 and concentrated under reduced pressure to give a 2,6-($HCF_2CF_2CH_2O$**CD_2**)$_2$-py as a colorless liquid.

Note: Synthesis of deuterated 2,6-($ClCF_2CF_2CH_2O$**CD_2**)$_2$-py and deuterated 2,6-($ClCF_2CH_2O$**CD_2**)$_2$-py ligand: The synthetic procedure was almost identical with that used for the deuterated 2,6-($HCF_2CF_2CH_2O$**CD_2**)$_2$-py ligand.

Analytical data for deuterated 2,6-($HCF_2CF_2CH_2OCD_2$)$_2$-py ligand: **^{1}H NMR** (400 MHz, $CDCl_3$, ppm): δ= 7.75 (1H, t, $^3J_{HH}$= 7.7 Hz, H4), 7.33 (2H, d, $^3J_{HH}$= 7.7 Hz, H3/5), 5.96 (2H, tt, $^3J_{HF}$= 53.2 Hz; $^4J_{HF}$= 5 Hz, $C_2F_4\underline{H}$), 3.93 (4H, t, $^3J_{HF}$= 12.5 Hz, $OC\underline{H}_2$). Yield = 85.0%.

Analytical data for deuterated 2,6-($ClCF_2CF_2CH_2OCD_2$)$_2$-py ligand: **^{1}H NMR** (400 MHz, $CDCl_3$, ppm): δ= 7.75 (1H, t, $^3J_{HH}$= 7.7 Hz, H4), 7.37 (2H, d, $^3J_{HH}$= 7.7 Hz, H3/5), 4.03 (4H, t, $^3J_{HF}$ = 14.2 Hz, $OCH_2CF_2CF_2Cl$). Yield = 85.0%.

Analytical data for deuterated 2,6-($ClCF_2CH_2OCD_2$)$_2$-py ligand: **^{1}H NMR** (400 MHz, $CDCl_3$, ppm): δ= 7.75 (1H, t, $^3J_{HH}$= 7.7 Hz, H4), 7.39 (2H, d, $^3J_{HH}$= 7.7 Hz, H3/5), 4.23 (4H, t, $^3J_{HF}$ = 11.9 Hz, OCH_2CF_2Cl). Yield = 85.0%.

Synthesis of deuterated 4FH-$ZnCl_2$(I) complex, [2,6-($HCF_2CF_2CH_2OCD_2$)$_2$-py-$ZnCl_2$],
A 25 mL single-necked round-bottomed flask was equipped with a magnetic stirrer and then filled sequentially with 2,6-($HCF_2CF_2CH_2OCD_2$)$_2$-py (0.27 mmol, 0.10 g), $ZnCl_2$ (0.27 mmol, 0.37 g) and 5 mL dichloromethane. Under N_2 atmosphere, this mixture was then stirred overnight with the round-bottomed flask immersed in an oil bath at room temperature. At the end of the reaction, the solvent was removed by vacuum system to leave an white powder as a crude product. The solid product was collected dried in an oven at 60 °C to give deuterated **4FH-$ZnCl_2$(I)** complex.

Note: Synthesis of **deuterated 4FCl-ZnI_2(II) complex**, [2,6-($ClCF_2CF_2CH_2O$**CD_2**)$_2$-py-ZnI_2], and **deuterated 2FCl-ZnI_2(III) complex**, [2,6-($ClCF_2CH_2O$**CD_2**)$_2$-py-ZnI_2]. The synthetic procedure was almost identical with that used for the **4FH-$ZnCl_2$(I)** complex.

Analytical data for deuterated 4FH-$ZnCl_2$(I) complex: **^{1}H NMR** (400 MHz, $CDCl_3$, ppm): δ= 8.04 (1H, t, $^3J_{HH}$= 8 Hz, H4), 7.40 (2H, d, $^3J_{HH}$= 8 Hz, H3/5), 6.16 (2H, tt, $^3J_{HF}$ = 52.6 Hz; $^4J_{HF}$= 4.9 Hz, $C_2F_4\underline{H}$), 3.93 (4H, t, $^3J_{HF}$= 12.5 Hz, $OC\underline{H}_2$). **FT-IR**, (ATR, cm^{-1}) ν = 3104, 3098 (sp^2 C–H), 3009 (C-H; –CF_2H), 2948, 2923, 2851 (sp^3 C–H), 2220, 1970 (CD_2; local mode), 1612 (C=C; –py), 1583 (C=N; –py), 1113, 1090, 1074, 1028 (C–F). Yield = 83.8%. m.p. = 109-112 °C.

Analytical data for deuterated 4FCl-ZnI_2(II) complex: **^{1}H NMR** (400 MHz, DMSO-d_6, ppm) δ= 7.89 (1H, t, $^3J_{HH}$= 7.7 Hz, H4), 7.37 (2H, d, $^3J_{HH}$= 7.7 Hz, H3/5), 4.30 (4H, t, $^3J_{HF}$ = 14.2 Hz, $OCH_2CF_2CF_2Cl$). **FT-IR**, (ATR, cm^{-1}) ν = 3093, 3074 (sp^2 C–H), 2958, 2926, 2853 (sp^3 C–H), 2165, 2050 (CD_2; local mode), 1610 (C=C; –py), 1582 (C=N; –py), 1147, 1123, 1096 (C–F). Yield = 80%. m.p. = 127 °C.

Analytical data for deuterated 2FCl-ZnI_2(III) complex: **^{1}H NMR** (400 MHz, DMSO-d_6, ppm) δ= 7.89 (t, $^3J_{HH}$= 7.7 Hz, 1H; H-4), 7.39 (d, $^3J_{HH}$= 7.7 Hz, 2H; H3/5), 4.28 (t, $^3J_{HF}$= 11.9 Hz, 4H; OCH_2CF_2Cl); **FT-IR**, (ATR, cm^{-1}) ν = 3117, 3047, 3019 (sp^2 C–H), 2953, 2925, 2870 (sp^3 C–H), 2194, 2074(CD_2; local mode), 1611 (C=C; –py), 1581 (C=N; –py), 119, 1112, 1094 (C–F); Yield= 80%. m.p. = 157 °C.

VII. NMR (^{1}H, ^{13}C and ^{19}F) spectra

1. **4FH-ZnCl$_2$(I)**

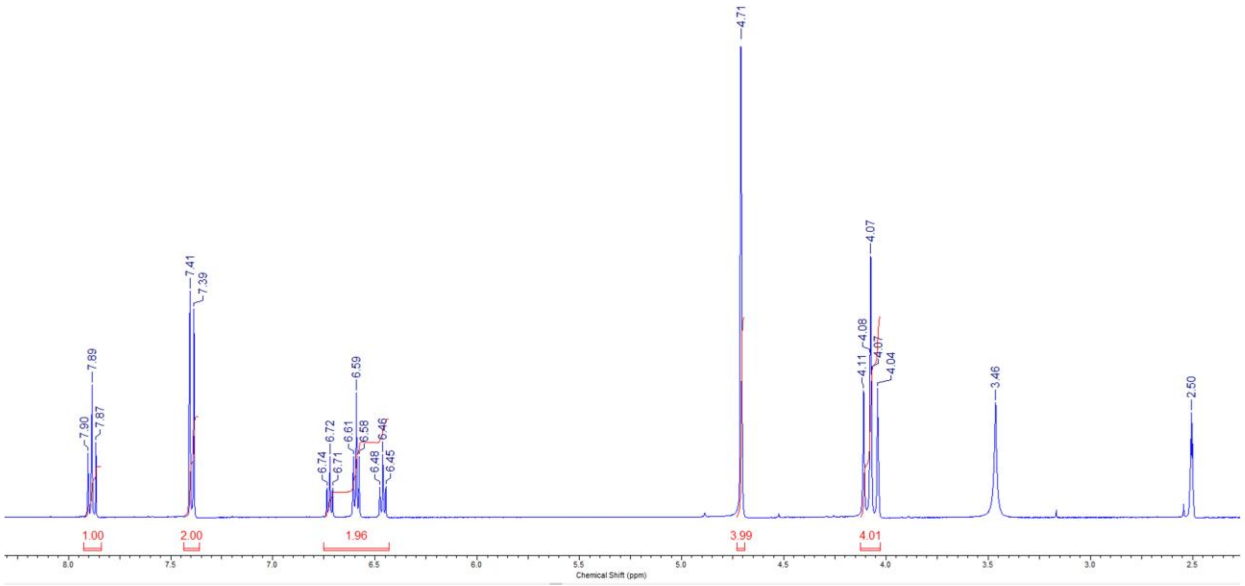

Figure S9. ^{1}H NMR spectrum of **4FH-ZnlCl$_2$(I)** complex.

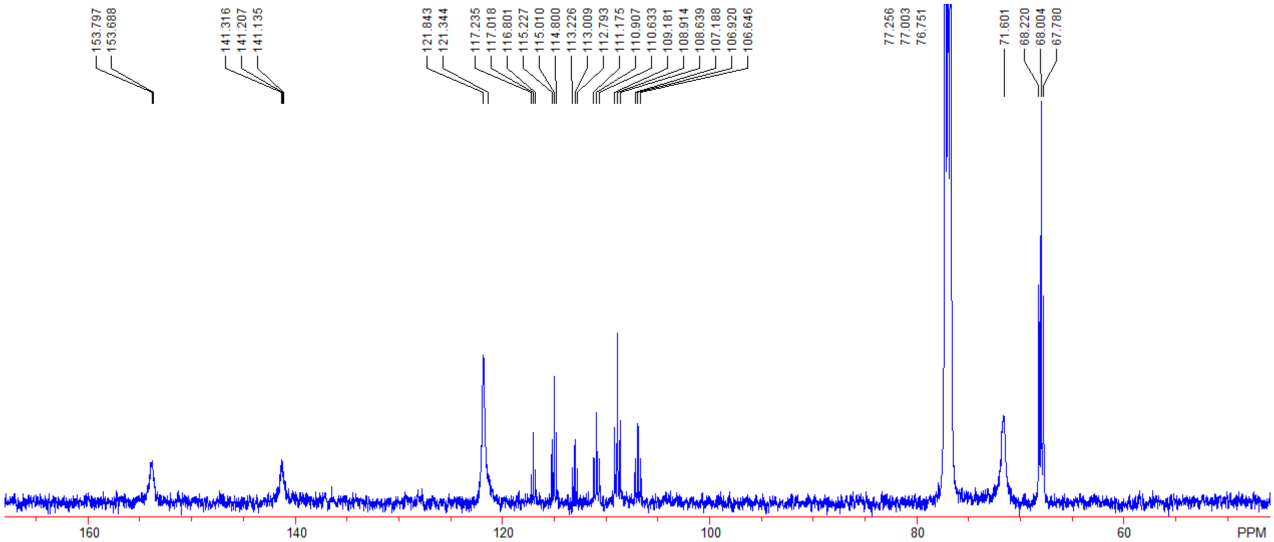

Figure S10. ^{13}C NMR spectrum of **4FH-ZnlCl$_2$(I)** complex.

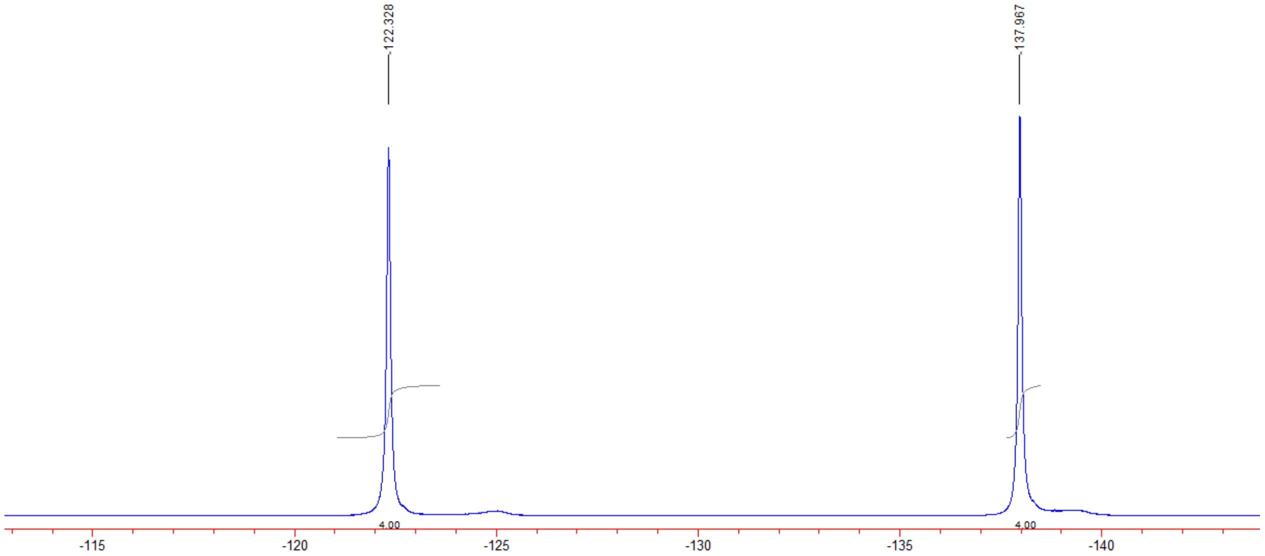

Figure S11. ^{19}F NMR spectrum of **4FH-ZnlCl$_2$**(**I**) complex.

2. 4FCl-ZnI$_2$(II)

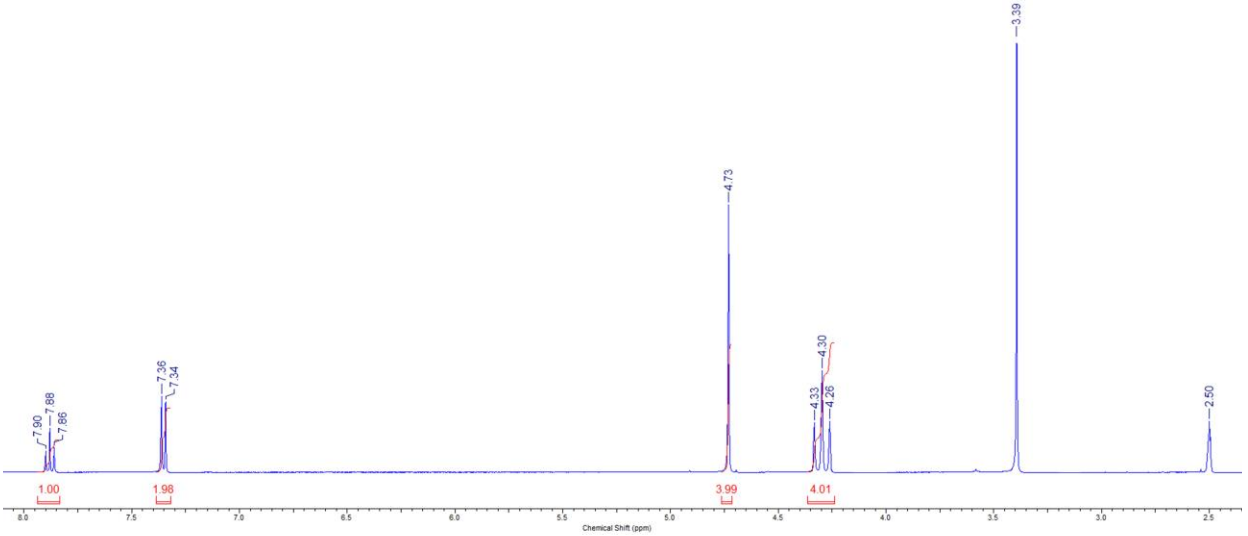

Figure S12. ^{1}H NMR spectrum of **4FCl-Znl$_2$**(**II**) complex.

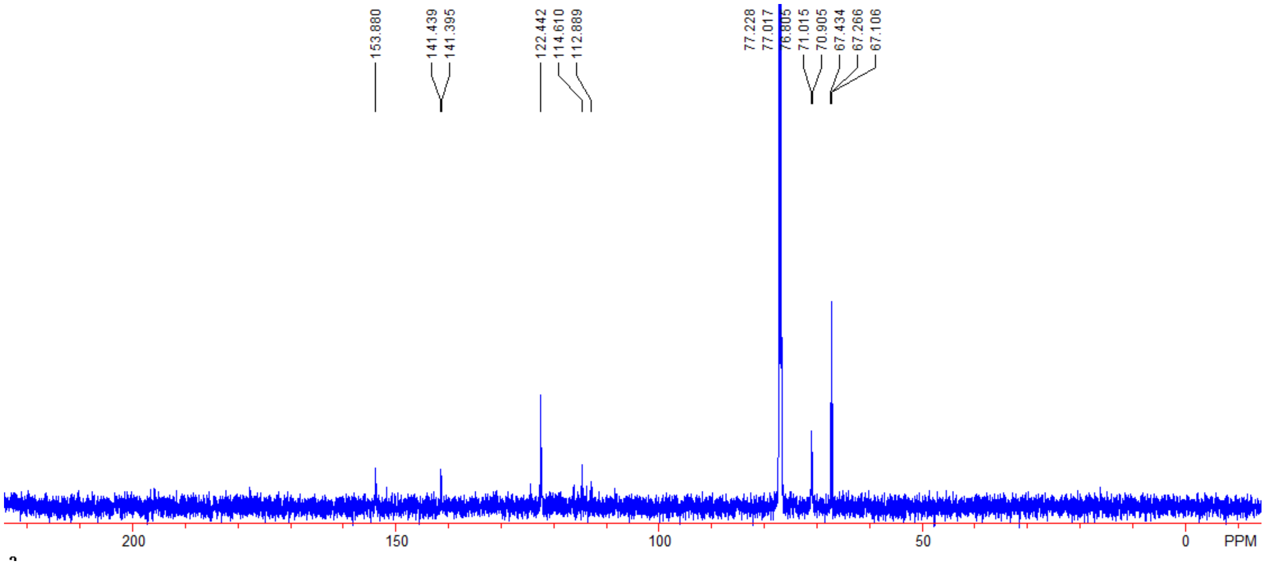

Figure S13. ^{13}C NMR spectrum of **4FCl-ZnI$_2$(II)** complex

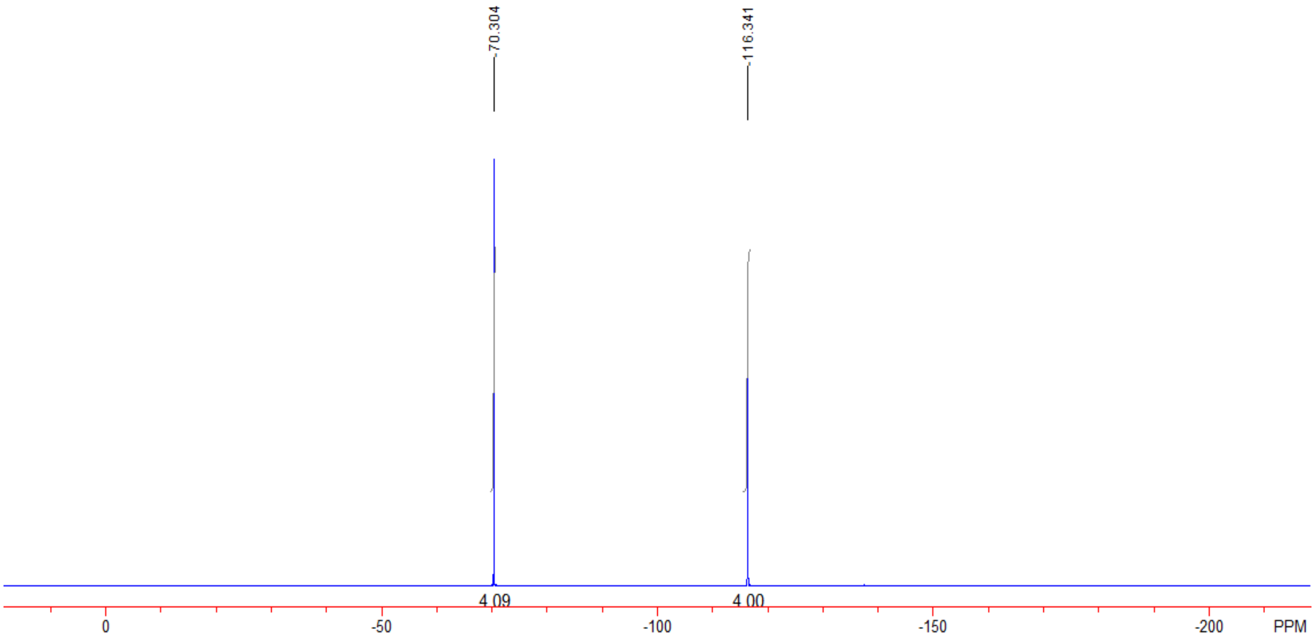

Figure S14. ^{19}F NMR spectrum of **4FCl-ZnI$_2$(II)** complex.

3. 2FCl-ZnI$_2$(III)

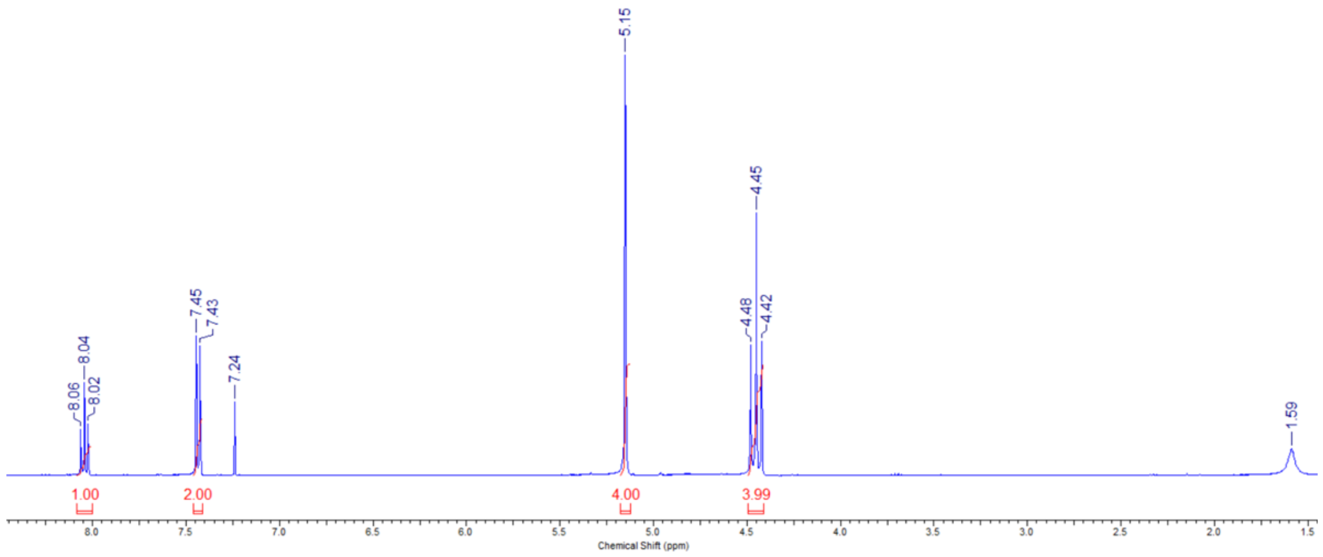

Figure S15. ^{1}H NMR spectrum of **2FCl-ZnI$_2$(III)** complex.

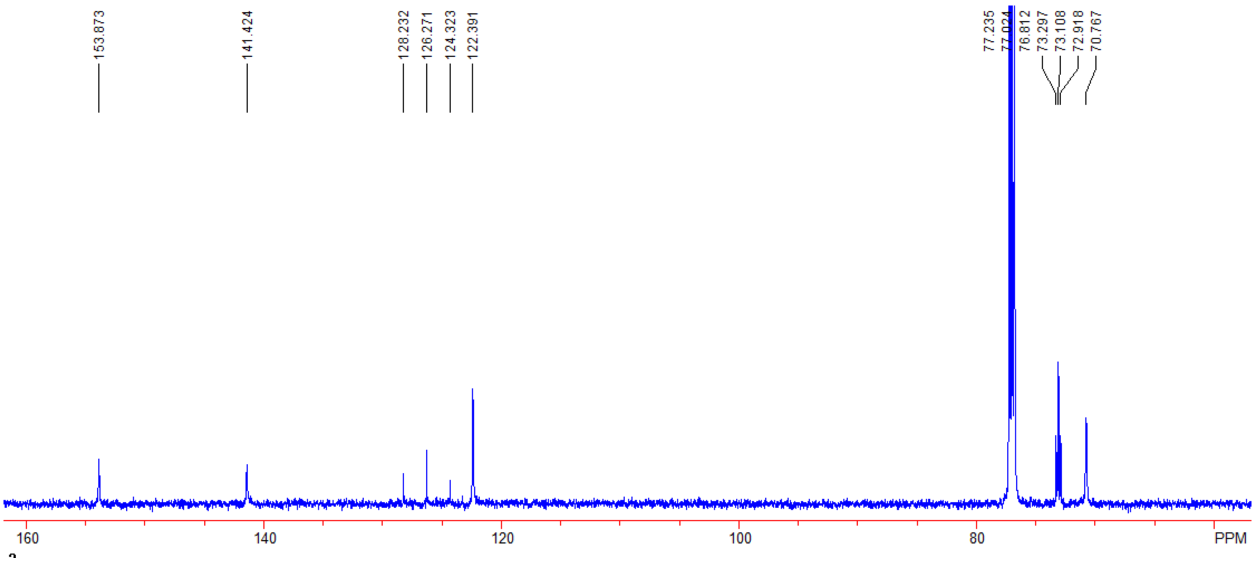

Figure S16. ^{13}C NMR spectrum of **2FCl-ZnI$_2$(III)** complex.

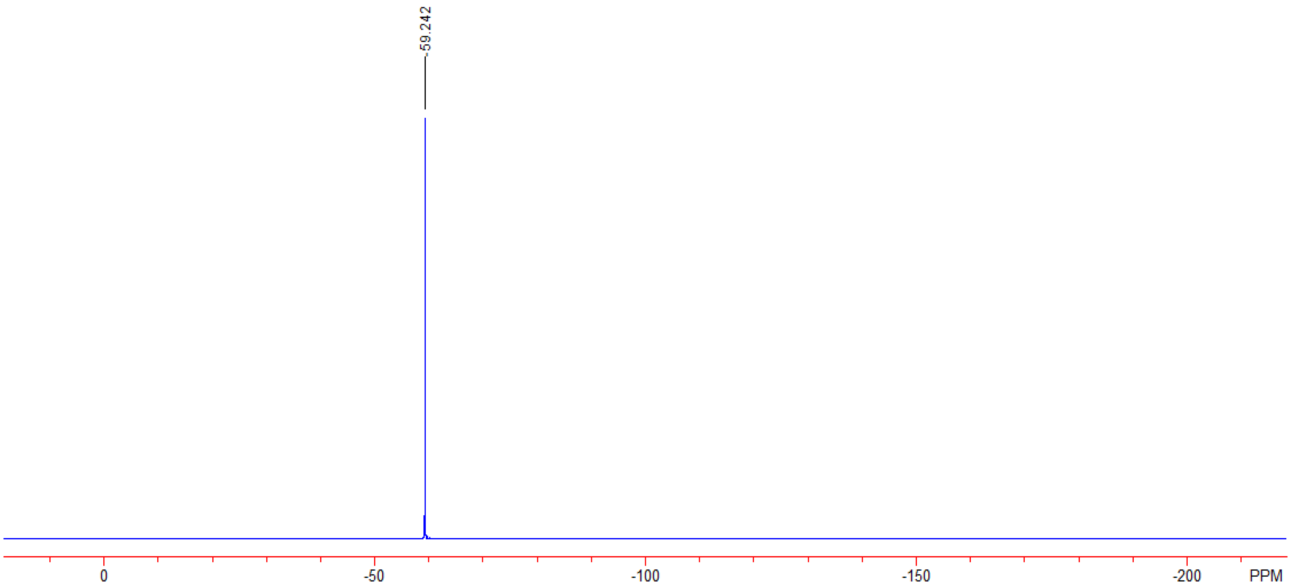

Figure S17. ^{19}F NMR spectrum of **2FCl-ZnI$_2$(III)** complex.

VIII. ^{1}H NMR spectra of deuterated ligands & complexes (I-III)

a. deuterated 2,6-($HCF_2CF_2CH_2OCD_2$)$_2$-py ligand & 4FH-$ZnCl_2$(I) complex

a.1. deuterated ligand

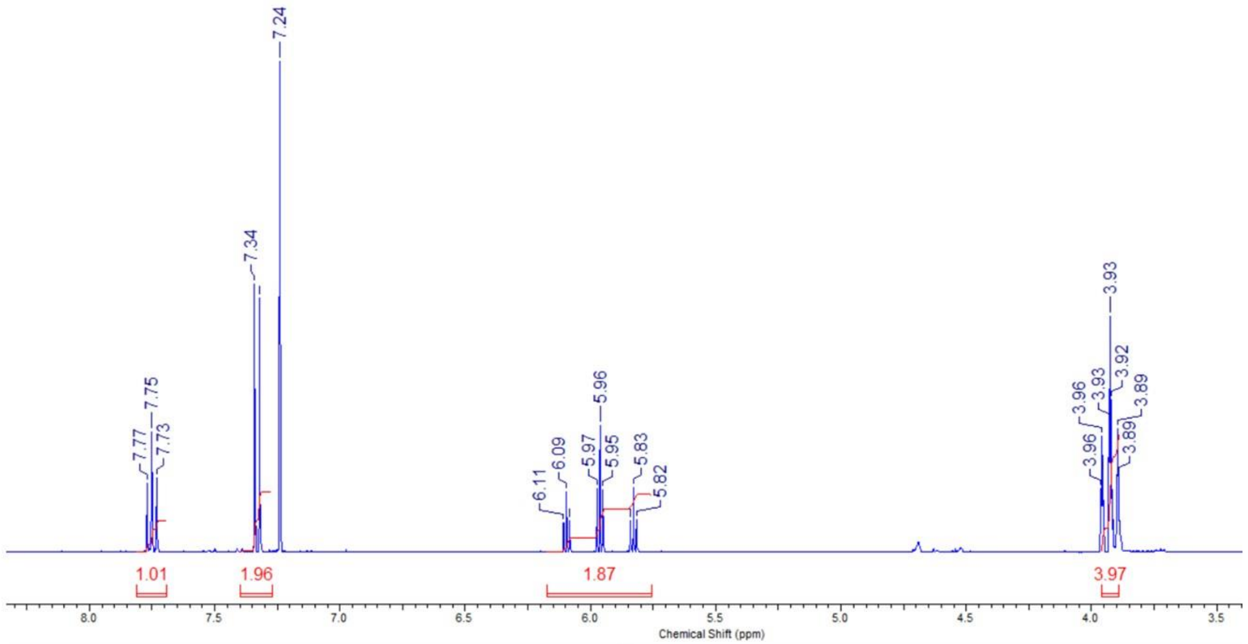

Figure S18. ^{1}H NMR spectrum of **deuterated 2,6-($HCF_2CF_2CH_2OCD_2$)$_2$-py** ligand.

a.2. deuterated 4FH-$ZnCl_2$(I) complex

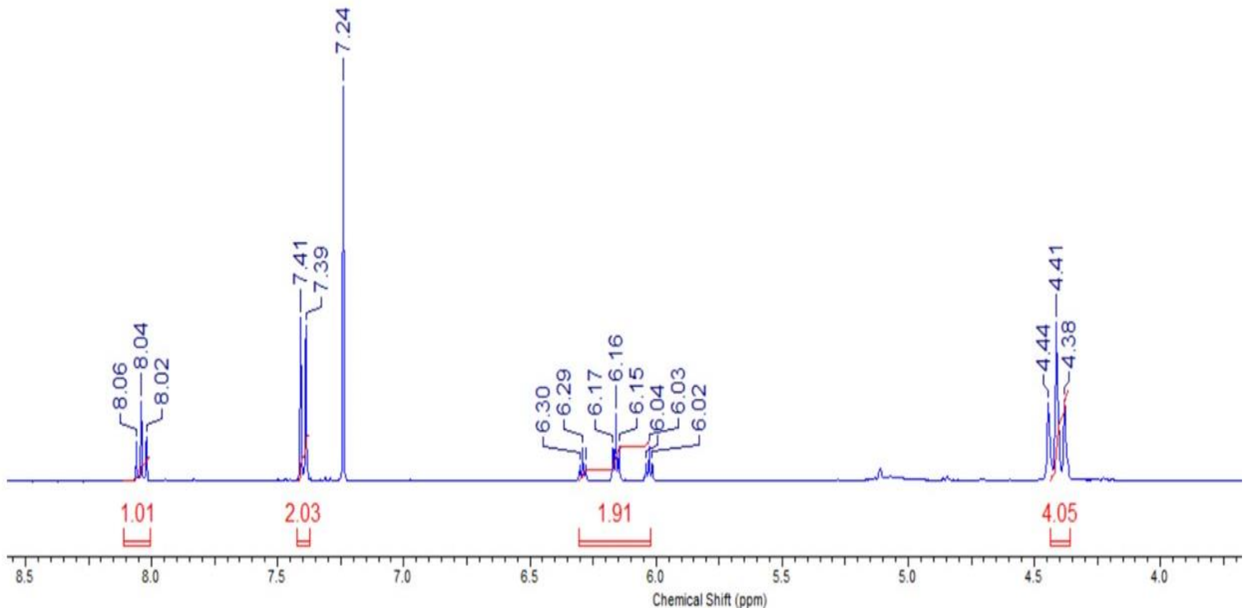

Figure S19. ^{1}H NMR spectrum of **deuterated 4FH-ZnlCl$_2$**(**I**) complex.

b. deuterated 2,6-($ClCF_2CF_2CH_2OCD_2$)$_2$-py ligand & 4FCl-ZnI$_2$(II) complex

b.1. deuterated ligand

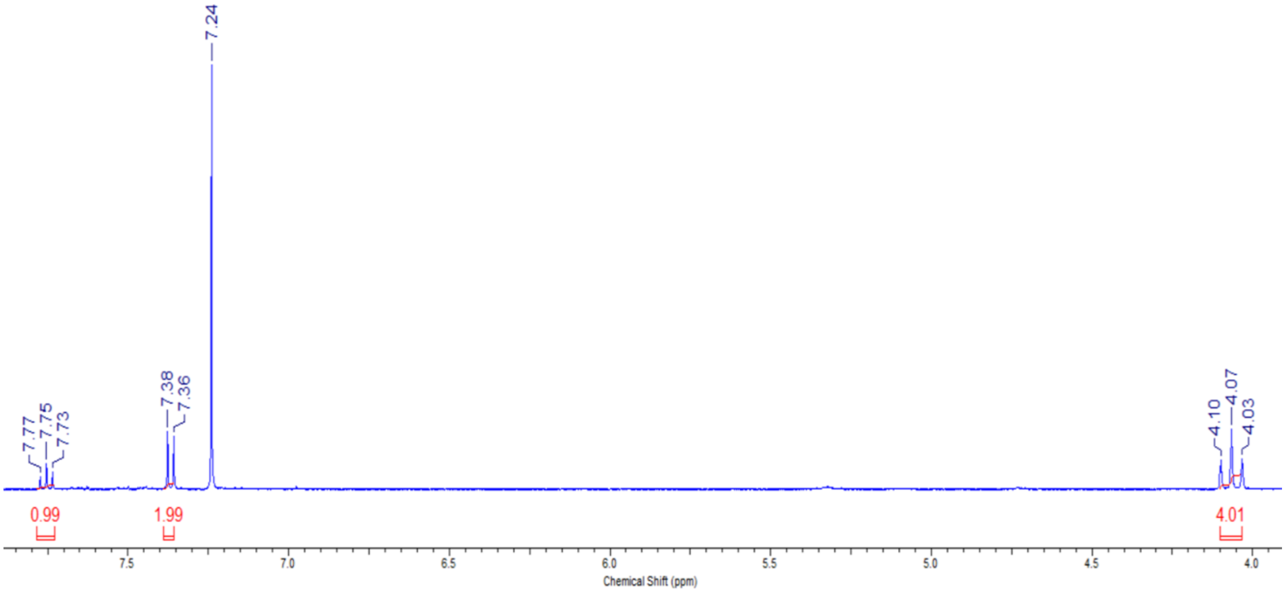

Figure S20. ^{1}H NMR spectrum of **deuterated 2,6-($ClCF_2CF_2CH_2OCD_2$)$_2$-py** ligand.

b.2. deuterated 4FCl-ZnI$_2$(II) complex

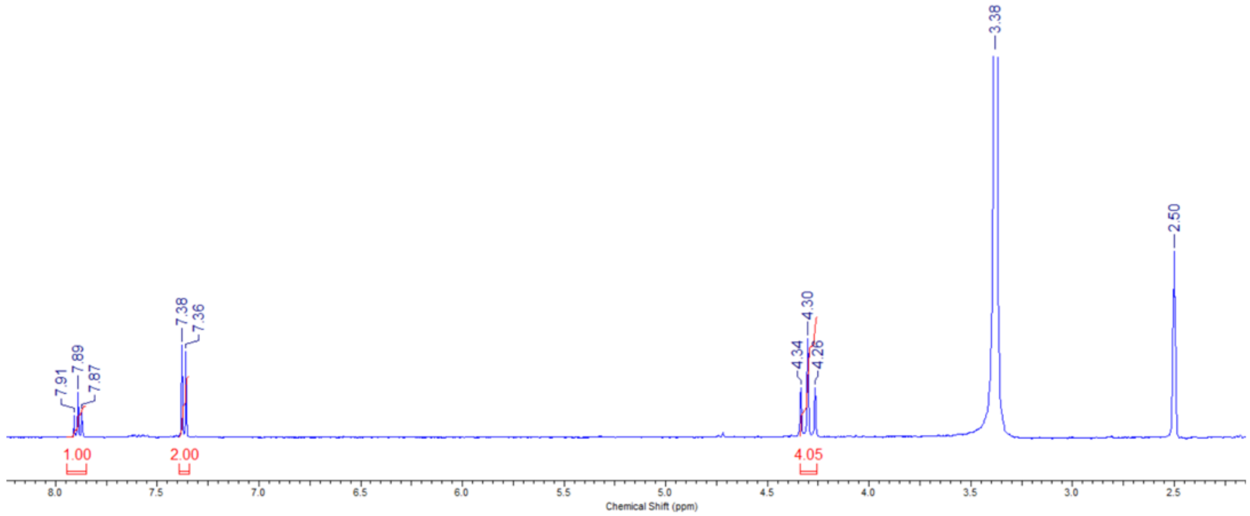

Figure S21. ^{1}H NMR spectrum of **deuterated 4FCl-ZnI$_2$(II)** complex.

c. deuterated 2,6-$(ClCF_2CH_2OCD_2)_2$-py ligand & 2FCl-ZnI_2(III) complex

c.1. deuterated ligand

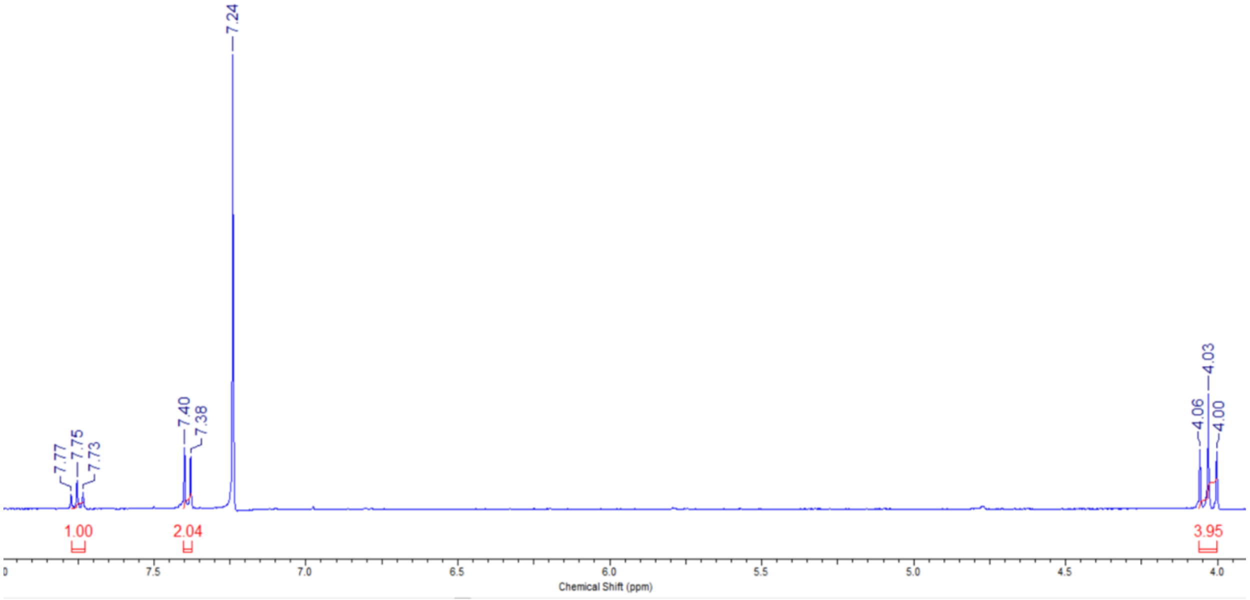

Figure S22. ^{1}H NMR spectrum of **deuterated 2,6-$(ClCF_2CH_2OCD_2)_2$-py** ligand.

c.2. deuterated 2FCl-ZnI_2(III) complex

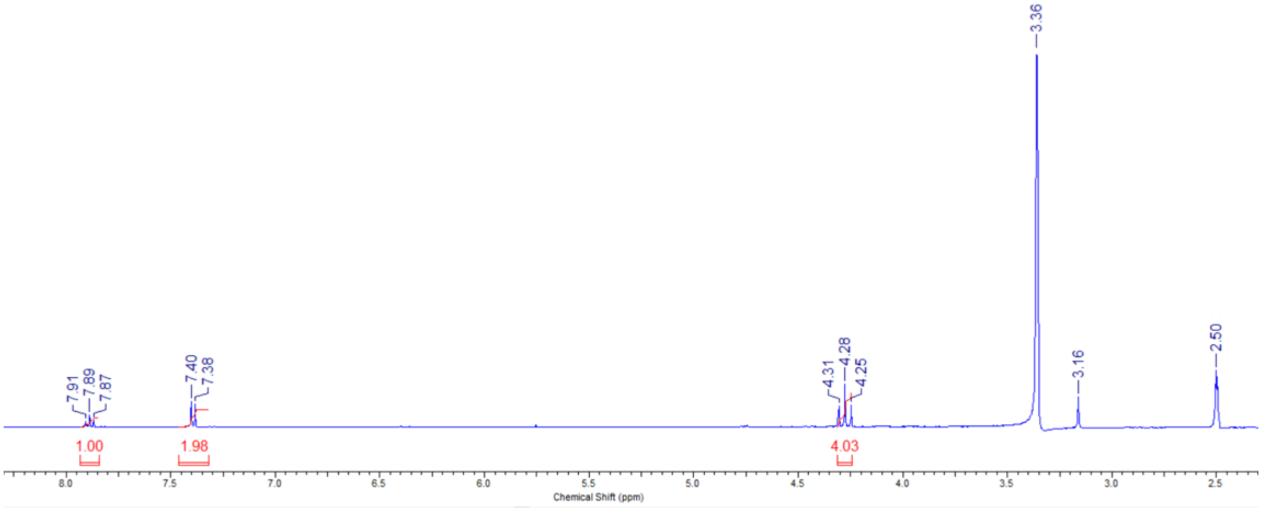

Figure S23. ^{1}H NMR spectrum of **deuterated 2FCl-ZnI_2(III)** complex.

IX. References

1. Elakkat, V.; Tessema, E.; Lin, C.H.; Wang, X.; Chang, H.C.; Zheng, Y.N.; Huang, Y.C.; Gurumallappa; Zhang, Z.Y.; Long Chan, K.; Rahayu, H.A.; Francisco, J. S.; Lu, N. Unusual Changes of C– H Bond Lengths in Chiral Zinc Complexes Induced by Noncovalent Interactions. *Angew. Chem. Int. Ed.* **2023**, *62*, e202215438.

2. Coates, L.; Cao, H.B.; Chakoumakos, B.C.; Frontzek, M.D.; Hoffmann, C.; Kovalevsky, A.Y.; Liu, Y.; Meilleur, F.; dos Santos, A.M.; Myles, D.A.; Wang, X.P.; Ye, F. A suite-level review of the neutron single-crystal diffraction instruments at Oak Ridge National Laboratory. *Rev. Sci. Instrum.* **2018**, *89*, 092802.

3. Schultz, A.J.; Jørgensen, M.R.V.; Wang, X.; Mikkelson, R.L.; Mikkelson, D.J.; Lynch, V.E.; Peterson, P.F.; Green, M.L.; Hoffmann, C.M. Integration of neutron time-of-flight single-crystal Bragg peaks in reciprocal space. *J. Appl. Crystallogr.* **2014**, *47*, 915–921.

4. Zikovsky, J.; Peterson, P.F.; Wang, X.P.; Frost, M.; Hoffmann, C. CrystalPlan: an experiment-planning tool for crystallography. *J. Appl. Crystallogr.* **2011**, 44, 418–423.

5. Schultz, A.J.; Srinivasan, K.; Teller, R.G.; Williams, J.M.; Lukehart, C.M. Single-crystal, time-of-flight, neutron-diffraction structure of hydrogen cis-diacetyltetracarbonylrhenate,[cis-(OC) 4Re $(CH_3CO)_2$]H: a metallaacetylacetone molecule. *J. Am. Chem. Soc.,* **1984**, 106, 999-1003.

6. Sheldrick, G. M. *Acta Crystallogr. Sect. A Struct. Chem.* **2015**, 71, 3–8.

7. Hübschle, C.B.; Sheldrick, G.M.; Dittrich, B. ShelXle: a Qt graphical user interface for SHELXL. *J. Appl. Crystallogr.* **2011**, 44, 1281–1284.

8. Ohhara, T.; Kiyanagi, R.; Oikawa, K.; Kaneko, K.; Kawasaki, T.; Tamura, I.; Nakao, A.; Hanashima, T.; Munakata, K.; Moyoshi, T.; Kuroda, T. SENJU: a new time-of-flight single-crystal neutron diffractometer at J-PARC. *J. Appl. Crystallogr.* **2016,** *49,* 120-127.

9. Ohhara, T.; Kusaka, K.; Hosoya, T.; Kurihara, K.; Tomoyori, K.; Niimura, N.; Tanaka, I.; Suzuki, J.; Nakatani, T.; Otomo, T.; Matsuoka, S.; Tomita, K.; Nishimaki, Y.; Ajima, T.; Ryufuku, S. Development of data processing software for a new TOF single crystal neutron diffractometer at J-PARC. *Nucl. Instr. Meth. Phys. Res. Sect. A* **2009,** *600,* 195-197.

10. Rodríguez-Carvajal, J. Recent advances in magnetic structure determination by neutron powder diffraction. Phys. B: Condens. Matter **1993**,192, 55.

11. Dunitz, J. D.; Maverick, E. F.; Trueblood, K. N. Atomic motions in molecular crystals from diffraction measurements. *Angew. Chem. Int. Ed.* **1988**, 27, 880–895.

12. Schomaker, V.; Trueblood, K. N. Correlation of internal torsional motion with overall molecular motion in crystals. *Acta Crystallogr. Sect. B Struct. Sci.* **1998**, 54, 507–514.

13. Spek, A. L. Platon/squeeze, *Acta Crystallogr. Sect. D Biol. Crystallogr.* **2009**, 65, 148–155.

14. Farrugia, L. J. WinGX and ORTEP for Windows: an update. *J. Appl. Crystallogr.* ***2012***, 45, 849–854.

15. Frisch, M.J.; Head-Gordon, M.; Pople, J.A. A direct MP2 gradient method. *Chem. Phys. Lett.* **1990**, 166, 275-280.

16. Frisch, M. J.; Trucks, G. W.; Schlegel, H. B.; Scuseria, G. E.; Robb, M. A.; Cheeseman, J. R.; Scalmani, G.; Barone, V.; Petersson, G. A.; Nakatsuji, H.; Li, X.; Caricato, M.; Marenich, A. V.; Bloino, J.; Janesko, B.G.; Gomperts, R.; Mennucci, B.; Hratchian, H. P.; Ortiz, J. V.; Izmaylov, A. F.; Sonnenberg, J. L.; Williams-Young, D.; Ding, F.; Lipparini, F.; Egidi, F.; Goings, J.; Peng, B.; Petrone, A.; Henderson, T.; Ranasinghe, D.; Zakrzewski, V. G.; Gao, J.; Rega, N.; Zheng, G.; Liang, W.; Hada, M.; Ehara, M.; Toyota, K.; Fukuda, R.; Hasegawa, J.; Ishida, M.; Nakajima, T.; Honda, Y.; Kitao, O.; Nakai, H.; Vreven, T.; Throssell, K.; Montgomery, J. A., Jr.; Peralta, J. E.; Ogliaro, F.; Bearpark, M. J.; Heyd, J. J.; Brothers, E. N.; Kudin, K. N.; Staroverov, V. N.; Keith, T. A.; Kobayashi, R.; Normand, J.; Raghavachari, K.; Rendell, A. P.; Burant, J. C.; Iyengar, S. S.; Tomasi, J.; Cossi, M.; Millam, J. M.; Klene, M.; Adamo, C.; Cammi, R.; Ochterski, J.W.; Martin, R. L.; Morokuma, K.; Farkas, O.; Foresman, J. B.; Fox, D. J. Gaussian, Inc.: Wallingford, CT, **2016**.

17. Dennington, R.; Keith T.; Millam, J. GaussView, Version 6.1.1, Semichem Inc., Shawnee Mission, KS, **2019**.

18. Hay, P. J.; Wadt, W. R. Ab initio effective core potentials for molecular calculations. Potentials for K to Au including the outermost core orbitals. *J. Chem. Phys.* **1985**, 82, 299-310.

19. Chai, J.-D.; Head-Gordon, M. Long-range corrected hybrid density functionals with damped atom–atom dispersion corrections. *Phys. Chem. Chem. Phys.* **2008**, 10, 6615−6620.

20. Kendall, R. A.; Dunning, T. H.; Harrison, R. J. Electron affinities of the first-row atoms revisited. Systematic basis sets and wave functions. *J. Chem. Phys.* **1992**, 96, 6796−6806.

21. Zou, W.; Tao, Y.; Freindorf, M.; Makoś, M. Z.; Verma, N.; Cremer, D.; Kraka, E.Local Vibrational Mode Analysis (LModeA. Computational and Theoretical Chemistry Group (CATCO)); Southern Methodist University: Dallas, TX, U.S.A., **2022**.

22. Tao, Y.; Zou, W.; Nanayakkara, S.; Kraka, E. LModeA-nano: A PyMOL Plugin for Calculating Bond Strength in Solids, Surfaces, and Molecules via Local Vibrational Mode Analysis. *J. Chem. Theory Comput.* **2022**, *18*, 1821–1837.

23. Moura Jr, R.T.; Quintano, M.; Antonio, J.J.; Freindorf, M.; Kraka, E., Automatic generation of local vibrational mode parameters: from small to large molecules and QM/MM systems. *J. Phys. Chem. A*, **2022**, 126, 9313-9331.

24. Kraka, E.; Freindorf, M.; Zou, W.; Konkoli, Z. Local Vibrational Mode Theory: An Alternative to Normal Mode Analysis for the Description of Vibrational Spectra. *J. Phys. Chem. A* **2022**, 126, 8781–8797.

25. Verma, N.; Tao, Y.; Zou, W.; Chen, X.; Chen, X.; Freindorf, M.; Kraka, E. A Critical Evaluation of Vibrational Stark Effect (VSE) Probes with the Local Vibrational Mode Theory. *Sensors* **2020**, 20, 2358.

26. a) Becke, A. D. Phys. Re V. A. *J. Chem. Phys.* **1992**, 96, 2155–2160; b) Thanthiriwatte, K.S.; Hohenstein, E.G.; Burns, L.A.; Sherrill, C.D.; Assessment of the performance of DFT and DFT-D methods for describing distance dependence of hydrogen-bonded interactions. *J. Chem. Theory Comput.* **2011**, 7, 88-96; c) Stephens, P.J.; Devlin, F.J.; Chabalowski, C.F.; Frisch, M.J. Ab initio calculation of vibrational absorption and circular dichroism spectra using density functional force fields. *J. Phys. Chem. A*, **1994**, 98, 11623-11627.

27. Grimme, S. Ehrlich, S. Goerigk, L. Effect of the damping function in dispersion corrected density functional theory. *J. Comput. Chem.* **2011**, 32, 1456–1465.

28. Krishnan, R.B.J.S.; Binkley, J.S.; Seeger, R.; Pople, J.A. Self-consistent molecular orbital methods. XX. A basis set for correlated wave functions. *J. Chem. Phys.* **1980**,72, 650-654.

29. Johnson, E.R.; Keinan, S.; Mori-Sánchez, P.; Contreras-García, J.; Cohen, A.J.; Yang, W. Revealing noncovalent interactions. *J. Am. Chem. Soc.* **2010**, 132, 6498–6506.

30. Humphrey, W.; Dalke, A.; Schulten, K., VMD: visual molecular dynamics. *J. Mol. Graph.* **1996**, 14, 33–38.

31. Kendall, R. A.; Dunning Jr, T. H.; Harrison, R. J. Electron affinities of the first-row atoms revisited. Systematic basis sets and wave functions. *J. Chem. Phys.***1992**. 96, 6796-6806.
